# *Salmonella* associated with agricultural animals exhibit diverse evolutionary rates and show evidence of recent clonal expansion

**DOI:** 10.1128/mbio.01913-24

**Published:** 2024-09-17

**Authors:** Ruixi Chen, Linghuan Yang, Magdalena S. Pajor, Martin Wiedmann, Renato H. Orsi

**Affiliations:** 1 Department of Food Science, Cornell University, Ithaca, New York, USA; University of Illinois Chicago, Chicago, Illinois, USA

**Keywords:** *Salmonella*, serovar, evolution, Enteritidis, Montevideo, Dublin, Infantis, Cerro, Reading, Kentucky, polyphyletic, BEAST, effective population size, whole-genome sequence, chicken, cattle, turkey, swine, pig, poultry, dairy

## Abstract

Most foodborne salmonellosis outbreaks are linked to agricultural animal products with a few serovars accounting for most *Salmonella* isolated from specific animal products, suggesting an adaptation to the corresponding animal hosts and their respective environments. Here, we utilized whole-genome sequence (WGS) data to analyze the evolution and population genetics of seven serovars frequently isolated from ground beef (Montevideo, Cerro, and Dublin), chicken (Kentucky, Infantis, and Enteritidis), and turkey (Reading) in the United States. In addition, publicly available metadata were used to characterize major clades within each serovar with regard to public health significance. Except for Dublin, all serovars were polyphyletic, comprising 2–6 phylogenetic groups. Further partitioning of the phylogenies identified 25 major clades, including 12 associated with animal or environmental niches. These 12 clades differed in evolutionary parameters (e.g., substitution rates) as well as public health relevant characteristics (e.g., association with human illness, antimicrobial resistance). Overall, our results highlight several critical trends: (i) the *Salmonella* generation time appears to be more dependent on source than serovar and (ii) all serovars contain clades and sub-clades that are estimated to have emerged after the year 1940 and that are enriched for isolates associated with humans, agricultural animals, antimicrobial resistance (AMR), and/or specific geographical regions. These findings suggest that serotyping alone does not provide enough resolution to differentiate isolates that may have evolved independently, present distinct geographic distribution and host association, and possibly have distinct public health significance.

**IMPORTANCE:**

Non-typhoidal *Salmonella* are major foodborne bacterial pathogens estimated to cause more than one million illnesses, thousands of hospitalizations, and hundreds of deaths annually in the United States. More than 70% of *Salmonella* outbreaks in the United States have been associated with agricultural animals. Certain serovars include persistent strains that have repeatedly contaminated beef, chicken, and turkey, causing outbreaks and sporadic cases over many years. These persistent strains represent a particular challenge to public health, as they are genetically clonal and widespread, making it difficult to differentiate distinct outbreak and contamination events using whole-genome sequence (WGS)-based subtyping methods (e.g., core genome allelic typing). Our results indicate that a phylogenetic approach is needed to investigate persistent strains and suggest that the association between a *Salmonella* serovar and an agricultural animal is driven by the expansion of clonal subtypes that likely became adapted to specific animals and associated environments.

## INTRODUCTION

*Salmonella enterica* subsp. *enterica* is incredibly diverse, comprising >1,500 unique serovars (1). Apart from typhoidal (Typhi) and paratyphoidal (Paratyphi A-C and Sendai) serovars, which cause enteric fever (2), all others are considered nontyphoidal *Salmonella* (NTS) serovars, which primarily cause gastroenteritis. While typhoidal and paratyphoidal serovars are restricted to humans as their exclusive reservoir (3), NTS serovars can be host-restricted, host-adapted, or host-promiscuous. For instance, *Salmonella enterica* subsp. *enterica* serovar (hereafter referred to as *S*.) Typhimurium, which caused an estimated 23.4% of human salmonellosis in the United States from 1996 to 2006 (4), represents a host-promiscuous NTS serovar frequently isolated from different hosts, including turkey, swine, cattle, and chicken (5). Conversely, *S*. Dublin is a cattle-adapted serovar responsible for fewer, but potentially more severe, human infections (6, 7). *S*. Gallinarum, which can only infect galliforme birds is an example of a host-restricted serovar (8).

NTS serovars pose a substantial public health concern and have been estimated to cause 1.35 million illnesses, 26,500 hospitalizations, and 420 deaths annually in the United States, representing the leading cause of bacterial foodborne illnesses and deaths in the United States (9, 10). Between 1998 and 2008, 72% of US *Salmonella* outbreaks with identifiable single-ingredient sources were linked to agricultural animal products, such as eggs, chicken, turkey, pork, and beef (11). Importantly, a number of NTS serovars associated with agricultural animals and their products (e.g., beef and poultry) were frequently implicated in *Salmonella* outbreaks (11, 12). For example, from 1998 to 2008, 64% of *Salmonella* outbreaks linked to chicken in the United States were attributed to serovars Enteritidis (28%), Typhimurium (23%), and Heidelberg (13%) (11). Likewise, among 96 salmonellosis outbreaks in the United States associated with beef consumption between 1973 and 2011, 42% were attributed to serovars Typhimurium (17%), Newport (16%), and Enteritidis (9%) (13). However, other NTS serovars are frequently associated with agricultural animals, and their products are rarely associated with outbreaks and human illness cases. For example, *S*. Kentucky has been the most prevalent serovar found in broiler chicken in the United States (14), but has rarely caused human disease in the United States (15); this has been attributed to the fact that the predominant *S*. Kentucky strain found in US chicken represents ST 152, which appear to show attenuated human virulence (16). Likewise, while *S*. Cerro is one of the most common *Salmonella* serovars obtained from healthy cattle in North America (17, 18), this serovar is also rarely associated with human illness (19). While serovar-based classification has clearly helped with identification of *Salmonella* subgroups that differ in their likelihood of causing human illnesses, the use of serovar-based approaches to identify *Salmonella* that differ in their public health risk is complicated as (i) *Salmonella* serovars can be polyphyletic, with isolates with identical antigenic formulae originating from distinct evolutionary lineages without a shared most recent common ancestor (MRCA) and (ii) even monophyletic *Salmonella* serovars may contain lineages with distinct phenotypic and public health relevant characteristics. The occurrence of polyphyletic *Salmonella* serovars is supported by Worley et al. (20), who identified 24 polyphyletic serovars among 247 distinct *Salmonella enterica* subsp. *enterica* serovars. A polyphyletic population structure has also been independently documented for several serovars including Newport (21), Mississippi (22), Saintpaul (23), and Bareilly (24). The potential importance of characterizing and defining polyphyletic serovars is particularly striking with regard to *S*. Kentucky, which includes two major lineages without a shared MRCA, with one lineage, *S*. Kentucky-1, including ST 152 and another lineage, *S*. Kentucky-2, including ST 198 showing very distinct characteristics, including related to virulence (15, 16).

Here, we selected seven serovars (i.e., Dublin, Cerro, Montevideo, Reading, Kentucky, Infantis, and Enteritidis) frequently isolated from sources related to cattle, chicken, and turkey in the United States, based on the results of a *Salmonella* risk-based sampling project conducted by the Food Safety and Inspection Service in 2014 (25). Whole-genome sequencing (WGS) data and metadata submitted to the National Center for Biotechnology Information (NCBI) Pathogen Detection (PD) database were utilized to provide a comprehensive overview of the evolution of these serovars. The population structure of each serovar was analyzed to identify phylogenetic groups (in cases of polyphyletic serovars) and clades within a given serovar, followed by characterization of the clades with respect to their (i) association with sources related to animals or non-animal, natural environment (hereafter referred to as environment), (ii) association with human illnesses, (iii) proportions of multidrug-resistant (MDR) isolates, and (iv) genetic diversity. We further performed evolutionary analysis for clades significantly associated with animal or environmental sources to understand their evolutionary history and identify sub-clades that likely experienced clonal expansion.

## MATERIALS AND METHODS

### Identification of candidate single nucleotide polymorphism clusters and singletons

The NCBI PD project (https://www.ncbi.nlm.nih.gov/pathogens/) clusters all isolates in its database into single nucleotide polymorphism (SNP) clusters by implementing single-linkage clustering with a cutoff of 25 whole-genome Multi-Locus Sequence Typing (wgMLST) alleles. Isolates not assigned to any SNP cluster are designated as singletons. On August 31, 2021, we obtained the metadata for all *Salmonella enterica* isolates available in the database to identify candidate SNP clusters and singletons for each of the seven serovars studied here (see Supplementary Information 1 for detailed procedures).

### *De novo* assembly, quality check, and *in silico* serotyping

For detailed phylogenetic analyses, a set of representative isolates (i.e., all singletons and isolates with the fewest number of contigs within their respective SNP cluster) was selected for each of the seven serovars studied. Genome assemblies of these isolates were acquired either by direct download from the NCBI PD database or by *de novo* assembly from raw sequence reads using SKESA v 2.4.0 (26); quality assessment was performed using QUAST v 5.1.0rcl (27). The serotype of isolates was confirmed through a customized *in silico* serotyping workflow (Fig. S24), which integrated SISTR v 1.1.1 (28), SeqSero2 v 1.2.1 (29), and additional methods. Details on the acquisition, quality assessment, and *in silico* serotyping of the genome assemblies are found in Supplementary Information 1.

### Reference-free core SNP variant calling and phylogenetic analyses

The framework described by Chen et al. (23) was used to obtain a comprehensive understanding of the phylogeny of each serovar. Specifically, phylogenetic analyses were performed using all representative isolates of a given serovar and a compilation of reference isolates [(22); Supplementary Information 1]. kSNP v 3.1 (30) was used to create a core SNP alignment among all representative isolates of each of the serovars and reference isolates. These core SNP alignments were used to construct maximum-likelihood phylogenies for each serovar using FastTree v 2.1.10 (31). Supplementary Information 1 provides details on the reference-free variant calling and phylogenetic reconstruction.

### Identification of phylogenetic groups and clades within serovars

The overall phylogenies described above were used to infer whether a given serovar is polyphyletic based on the tree topology and local support values. If a given serovar was polyphyletic, distinct phylogenetic groups and standalone singletons were identified (Supplementary Information 1). To compare between the seven serovars of interest, another reference-free core SNP variant calling and maximum-likelihood phylogeny reconstruction were performed as previously described (23). This analysis included the genome sequences of representative isolates from each phylogenetic group and standalone singletons identified in the analysis described above for the serovars of interest, and the set of reference isolates (22) described above.

The downstream analyses of a given serovar focused on the phylogenetic group(s) comprising the majority of isolates. To assess the relatedness of isolates within a specific phylogenetic group, a maximum-likelihood phylogeny was reconstructed using representative isolates within the group and closely related reference isolate(s). Bayesian analysis of population structure was subsequently performed, using the fastbaps v 1.0.6 package (32) in R Statistical Programming Environment (R) v 4.1.1 (33), to divide each given phylogenetic group into three nested levels of clades, which were visually examined to identify target clades for further characterizations. Supplementary Information 1 provides details on the procedures used for identification of phylogenetic groups and clades.

### Assessment of genetic diversity

The genetic diversity of each clade was determined by calculating Simpson’s Diversity Index, wherein each of the SNP clusters and singletons was treated as a distinct “subtype.” The resulting diversity index was then subtracted from 1 to derive a “Clonal Index,” which indicates the degree of clonality of a given clade.

### Identification of clades associated with animal or environmental sources

The metadata associated with US non-human *Salmonella enterica* isolates available in the NCBI PD database (accessed on August 31, 2021) included 6,348 and 160 unique and non-standardized expressions in the “isolation_source” (e.g., “Product-Raw-Ground, Comminuted or Otherwise Nonintact-Pork”) and “host” (e.g., “Domestic Cattle”) fields, respectively. To allow for formal analyses, we identified 29 standardized terms for isolation source (e.g., “Pork”) and 18 standardized terms for hosts (e.g., “Cattle”) that were associated with the different terms used in NCBI. Non-standardized expressions were then matched to the standardized terms to classify isolates as originating from cattle, swine, chicken, or turkey. Isolates with non-standardized expressions that could not be matched to any of the standardized terms were classified as “other,” which occasionally represented natural environmental sources unrelated to major agricultural animals.

To identify clades associated with specific animal or environmental sources across different serovars, clades with >100 non-human isolates were assessed for over- or underrepresentation of isolates collected from each animal or environmental source category. Specifically, the odds ratio (OR) of isolates associated with a specific source category was calculated for each clade by dividing the odds of isolates assigned to a category among all non-human isolates in the clade by the odds of isolates assigned to the category among all *Salmonella* non-human isolates available in the NCBI PD database not belonging to the clade. Based on the odds ratios, a one-sided Fisher’s exact test was performed, with Benjamini-Hochberg (BH) correction for multiple testing, to determine whether a clade showed a significant overrepresentation (for odds ratios > 1) or underrepresentation (for odds ratios < 1) of isolates belonging to a source category. A given animal or environmental source category was considered (i) overrepresented among isolates within a given clade provided an odds ratio >5 and a BH-corrected *P*-value < 0.05 or (ii) underrepresented provided an odds ratio <0.2 and a BH-corrected *P*-value < 0.05. Unless otherwise specified, clades with 100 or fewer non-human isolates were not assessed for association with animal or environmental source categories due to their relatively small sample size.

Notably, isolates of a given serovar may be oversampled over a short period of time from a specific commodity, for example, due to sampling linked to a suspected or known outbreak associated with a specific food or serotype (e.g., by regulatory agencies during traceback investigations). Episodic oversampling could introduce bias that could lead to incorrect identification of associations between *Salmonella* clades and source categories. We hence performed a post hoc identification of potential sampling biases in our analyses; this was performed by identifying months representing high outliers using the interquartile range rule for each of the 12 clades associated with animal or environmental sources. The odds ratios were then re-calculated as described above, but without high outliers.

Additional information on the process of creating keyword lists, categorizing isolates into different source categories, evaluating the association between clades and isolation source categories, and confirming the identified associations is found in Supplementary Information 1.

### Assessment of public health significance

The likelihood of causing human illnesses was considered a primary measure for inferring public health significance. Hence, to determine whether isolates within each phylogenetic group differ in their public health significance, we computed the odds ratio of human isolates for each clade (i.e., the odds of human isolates among all isolates in the clade divided by the odds of human isolates among all *Salmonella* isolates not belonging to the clade), followed by using the Fisher’s exact test, along with the BH method for multiple correction, to determine statistical significance. Notably, although 81% of the isolates in the PD database originated from the United States or the United Kingdom, the proportion of human isolates among all uploaded isolates varied considerably between the two countries (70% and 93% of US and UK isolates in NCBI were classified as human isolates). As a result, odds ratios and BH-corrected *P*-values were calculated for each clade based on isolates collected from either the United States or the United Kingdom, whichever had the greater number of isolates for a given clade. Clades that had an odds ratio >2 with BH-corrected *P*-value < 0.05 were deemed to exhibit enrichment for human isolates, signifying an enhanced public health significance.

Importantly, the PD database may contain sampling biases due to the addition of human isolates during outbreaks. To mitigate the potential impacts of such biases, we conducted a post hoc evaluation of the clades showing enrichment for human isolates, leveraging the fact that outbreaks often result in an influx of human isolates within a specific clade over a short period of time. Specifically, we defined the so-called “high outliers” as the months that showed disproportionately high proportions of human isolates within the clade compared to all human isolates. For each clade of interest, we identified the pertinent outliers using the interquartile range rule based on isolates collected from the predominant country, and we confirmed the enrichment of human isolates within the clade by conducting the identical analysis (i.e., odds ratios calculations and the subsequent Fisher’s exact tests) with the high outliers excluded.

### *In silico* analysis of antimicrobial resistance

To identify AMR determinants and MDR isolates in each clade, the presence/absence data for highly curated AMR determinants (i.e., genes or point mutations) from the Pathogen Detection Reference Gene Catalog (https://www.ncbi.nlm.nih.gov/pathogens/) was extracted for each isolate from the metadata. Each distinct AMR determinant was linked to a drug class defined by the Comprehensive Antibiotic Resistance Database [CARD; (34)], to quantify the number of drug classes a given isolate was resistant to. Isolates possessing AMR determinants responsible for at least three CARD drug classes were deemed MDR isolates.

### Evolutionary analysis

Clades exhibiting an overrepresentation of isolates associated with any of the five source categories were selected as the focus of the evolutionary analysis. For each clade, we (i) estimated critical evolutionary parameters such as the time to the MRCA (TMRCA) and substitution rate and (ii) reconstructed the past population dynamics and time-scaled phylogeny. Paired-end reads of 100 representative isolates for each clade were downloaded from the NCBI Short Read Archive (SRA) and pre-processed using fastp v 0.23.2 (35). To generate a core genome alignment among representative isolates, reference-based variant calling was conducted using Snippy v 4.3.6 (36). Gubbins v 3.1.2 (37) was employed to identify and remove recombination sites within the core genome alignment, and variant sites of the filtered core genome alignment were subsequently extracted using snp-sites v 2.5.1 (38).

To verify the suitability of representative isolates for the evolutionary analysis of a given clade, the date-randomization test (DRT; Fig. S25) was performed to assess the molecular clock assumption, which determined whether the sampling dates of the representative isolates provided sufficient temporal signal for measuring evolutionary changes (39). The optimal substitution model and molecular clock settings were determined using bModelTest v 1.1.2 (40), and the marginal likelihood of population models was estimated using stepping stone sampling analysis to calculate the Bayes factor for determining the optimal population model (41). For each clade, the past population dynamics and time-scaled phylogeny were then reconstructed using BEAST v 2.5.2 (42). Detailed procedures on selecting representative isolates, generating recombination-free core SNP alignments, conducting DRT, optimizing the substitution model and molecular clock, comparing population models, and reconstructing past population dynamics and time-scaled phylogeny are found in Supplementary Information 1.

### Identification of clonal sub-clades within clades associated with animal sources

To ascertain the presence, within the animal-associated clades, of clonal sub-clades that showed significant associations with human illnesses or animal sources, the reference-based core SNP alignment and time-scaled phylogeny of each clade were utilized to identify sub-clades of isolates with low genetic distances (<5% of the total number of core SNPs identified among the 100 representative isolates of the clade). Subsequently, clonal sub-clades enriched for isolates from human or animal sources were identified based on (i) number of isolates, (ii) posterior probability for branch support, (iii) maximum pairwise genetic distance, and (iv) enrichment of human- or animal-associated isolates as compared to the baseline isolates (i.e., isolates within the same clade, but not part of any clonal sub-clades), with enrichment defined as proportional increase for a given category by >20 percentage points compared to the baseline level. Complete procedures and criteria involved in this analysis are detailed in Supplementary Information 1.

### Statistical analysis

A series of linear regression models were developed to examine the potential impact of various populational and evolutionary factors on the likelihood of a given clade causing human illnesses. Each model utilized the odds ratio between human and non-human isolates as the response variable and one specific factor of interest (i.e., association with animal or environmental sources, TMRCA, source category, MDR level, or clonality level) as the explanatory variable. All models were built using the stats v 4.2.1 package in R (33) The significance threshold for all statistical tests was set to a *P*-value of 0.05.

## RESULTS

### Overview of the phylogeny, host association, genetic diversity, and public health significance of *Salmonella* serovars associated with agricultural animals

**Fig 1:**
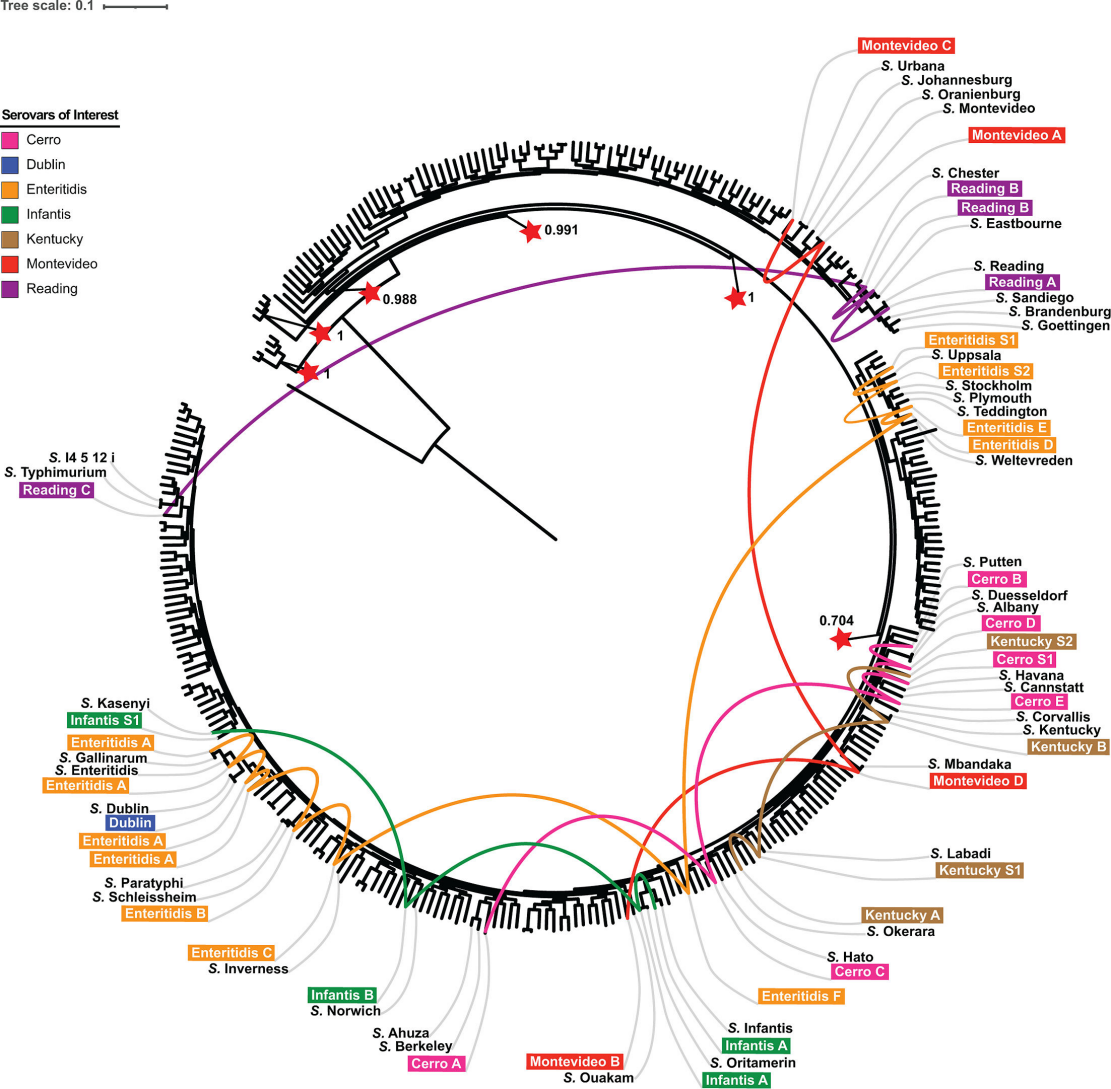
Overall maximum likelihood phylogeny of beef and poultry-associated NTS. The maximum likelihood phylogeny was reconstructed based on 22,364 core SNPs identified among representative isolates of phylogenetic groups and singletons of all seven serovars of interest, as well as reference isolates for 285 unique *Salmonella enterica* subsp. *enterica* serovars and one additional *Salmonella enterica* subspecies (*Salmonella enterica* subsp. *indica*). Branch lengths represent the average pairwise number of nucleotide substitutions per site. *Salmonella enterica* subsp. *indica*, the outgroup, was used to root the tree. The clustering confidence was assessed using the Shimodaira-Hasegawa (SH) test with 1,000 resamples. Red stars with the corresponding bootstrap values indicate deep splits that separate major clades of *Salmonella enterica* subsp. *enterica*. Tip labels for the phylogenetic groups [designated using the serovar name followed by a letter (e.g., A)] and singletons [designated using the serovar name followed by the letter S and an Arabic numeral (e.g., S1)] of the 7 serovars of interest are shown in distinct colors. Closely related serovars (including the reference isolates of the seven serovars of interest) are labeled in black. Phylogenetic groups that are monophyletic (e.g., Montevideo A) were represented by a single isolate, while paraphyletic groups (e.g., Enteritidis A) were represented by multiple isolates to display their phylogenetic relationship with other serovars clustering within the group. For each polyphyletic serovar, curved lines in the same color connect different phylogenetic groups and singletons.

**Fig 2:**
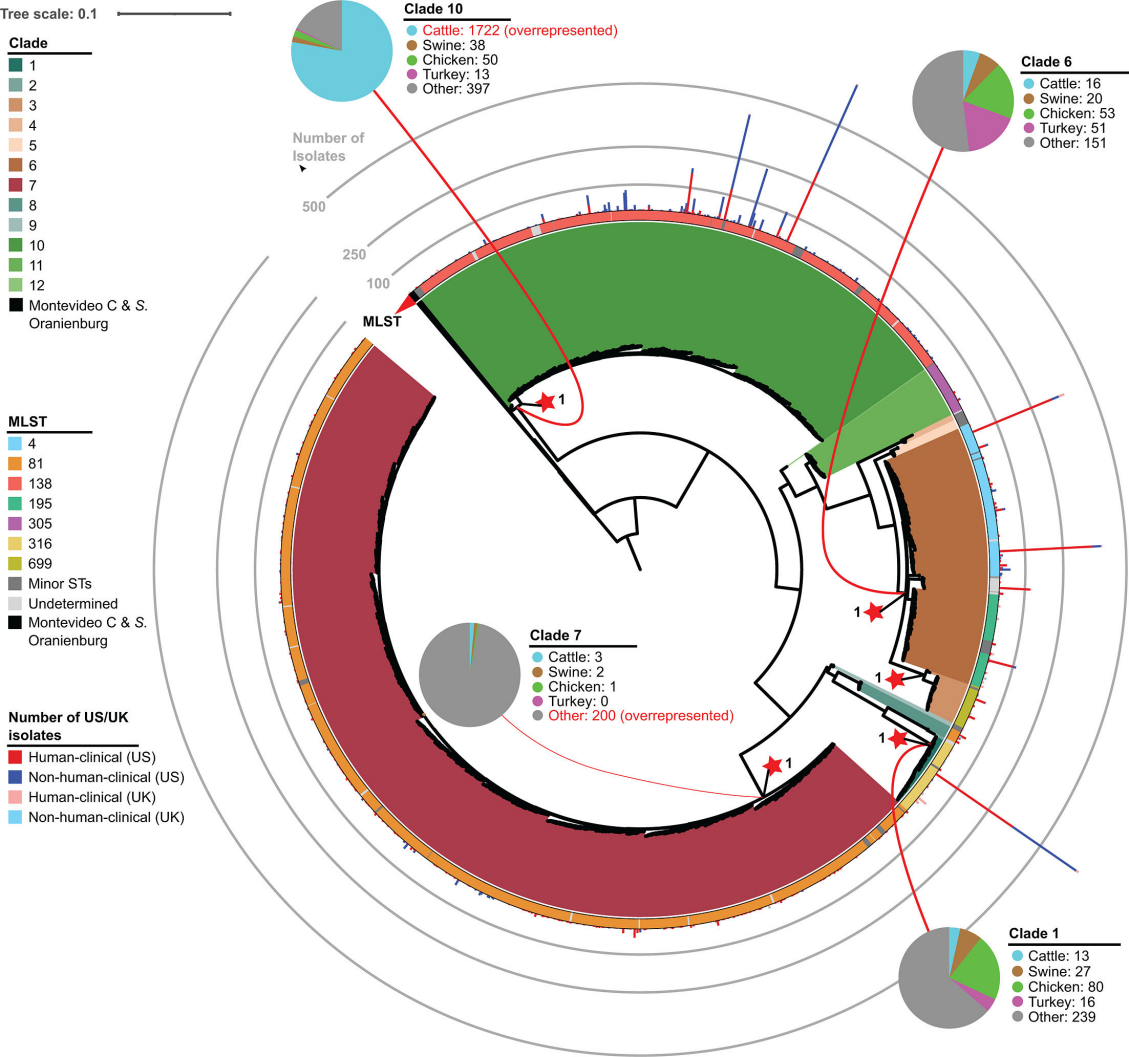
Maximum likelihood phylogeny for Montevideo A. Montevideo A is a monophyletic group comprising 12 clades, two of which are associated with cattle-related (clade 10) and environmental (clade 7) sources, respectively. The maximum likelihood phylogeny was reconstructed based on 45,469 core SNPs identified among isolates representing each of the SNP clusters and singletons from Montevideo A, as well as the outgroup (Montevideo C, and *S.* Oranienburg). The tree was rooted using Montevideo C, and the clustering confidence was assessed using the Shimodaira-Hasegawa (SH) test with 1,000 resamples. Branch lengths represent the average pairwise number of nucleotide substitutions per site. Color shades for the internal color strip indicate different clades, while black corresponds to the outgroup. Red stars followed by bootstrap values at internal nodes are used to describe the most recent common ancestor (MRCA) of the five clades with >100 isolates. The outer color strip represents the sequence type (ST) of the representative isolates. “Minor STs” represent STs accounting for ≤10 representative isolates. “Undetermined” designates isolates that could not be assigned to a ST. Stacked bars show the number of human and non-human isolates collected from the United States (red: human isolates; blue: non-human isolates) or UK (light red: human isolates; light blue: non-human isolates) for each SNP cluster. Pie charts exhibit the distribution of non-human isolates across five isolation source categories (i.e., cattle, swine, chicken, turkey, and other) for each clade with >100 non-human isolates (connected to the MRCA of the respective clade). Source categories that are over- or underrepresented within a clade are indicated in red font.

NTS serovars associated with major agricultural animals tend to be polyphyletic, comprising multiple phylogenetic groups that occasionally span multiple major clades of *Salmonella enterica* subsp. *enterica*. While *S*. Dublin was monophyletic, serovars Infantis, Kentucky, Reading, Montevideo, Cerro, and Enteritidis comprised 2, 2, 3, 4, 5, and 6 phylogenetic groups. Isolates were disproportionately distributed across different phylogenetic groups within a given serovar Table 1; Fig. 1; see Fig. S1 through S7 in supplementary materials for maximum-likelihood phylogenies for each serovar). Therefore, subsequent analyses were primarily focused on *S.* Dublin and the phylogenetic groups that represented most isolates from a given serovar, namely Cerro A, Enteritidis A, Infantis A, Montevideo A, Kentucky A and B, and Reading A, B, and C (Fig. 2; Fig. S8 through S16; see Data Set S1 for the summary statistics of all SNP clusters and singletons within each serovar/phylogenetic group). Among these 10 phylogenetic groups (including *S.* Dublin), 25 clades with >100 isolates were identified (Table 2). Twelve of these 25 clades were determined to be associated with one of the five source categories (i.e., cattle, chicken, swine, turkey, and environment; Table 3 ; Table S1). *S*. Montevideo phylogenetic group A clade 7 (Montevideo-A-7) was found to be associated with environment (e.g., water, sediment); Reading-A-1-1-2 was associated with turkey; Cerro-A-3, Infantis-A-1-2, and Reading-C-1-1-4 were associated with swine; Enteritidis-A-7, Infantis-A-1-3, and Kentucky-A-1 were associated with chicken; and Cerro-A-2, Dublin-2-3, Kentucky-B-2, and Montevideo-A-10 were associated with cattle. The remaining 13 clades with >100 isolates displayed no association with any specific source categories.

**TABLE 1:** Isolates, SNP clusters, and singletons available in NCBI pathogen detection by serovars and phylogenetic groups

Serovar (number of phylogenetic groups)	Phylogenetic groups	Number of SNP clusters	Number of singletons	Total number of isolates
Cerro (5)		207	539	1,902
	Cerro A	198	527	1,764
	Cerro B	6	8	124
	Cerro C	2	1	8
	Cerro D	1	0	3
	Cerro E	0	2	2
Dublin (1)		171	279	4,436
Enteritidis (6)		905	1,382	66,210
	Enteritidis A	882	1,291	66,058
	Enteritidis B	11	33	60
	Enteritidis C	5	30	41
	Enteritidis D	4	17	31
	Enteritidis E	2	9	15
	Enteritidis F	1	0	3
Infantis (2)		420	742	18,785
	Infantis A	417	738	18,769
	Infantis B	3	3	15
Kentucky (2)		279	443	10,050
	Kentucky A	238	372	8,314
	Kentucky B	41	69	1,734
Montevideo (4)		489	1,075	7,618
	Montevideo A	484	1,068	7,594
	Montevideo B	3	4	14
	Montevideo C	1	3	8
	Montevideo D	1	0	2
Reading (3)		60	84	2,792
	Reading A	7	7	2,072
	Reading B	40	60	369
	Reading C	13	17	351

**TABLE 2:** Characteristics of the 25 clades (with >100 isolates) identified within the seven serovars of interest

Phylogenetic group	Clade	Most common ST (proportion)^*a*^	Number of isolates			OR of human isolates^*c*^	OR of human isolates after removing outliers^*d*^	Number of MDR isolates (proportion)^*e*^	Clonal index	Association with source categories
			Total^*b*^	US	UK					
Cerro										
Cerro A	2	367 (0.93)	1,554	1,474	18	0.048	NC^*f*^	1 (0.001)	0.011	Yes
Cerro A	3	1593 (1)	164	123	24	0.184	NC	12 (0.073)	0.762	Yes
Dublin										
Dublin	2-2	10 (0.88)	201	35	0	14.534	14.107	6 (0.030)	0.014	No
Dublin	2-3	10 (0.97)	2,933	2,593	9	0.257	NC	2,601 (0.887)	0.742	Yes
Dublin	2-5	10 (0.89)	1,001	3	141	1.641	NC	19 (0.030)	0.148	No
Enteritidis										
Enteritidis A	1	11 (1)	107	105	0	2.779	2.504	0 (0)	0.017	No
Enteritidis A	6	183 (0.96)	785	8	639	0.456	NC	3 (0.004)	0.036	No
Enteritidis A	7	11 (0.92)	64,794	35,203	16,840	2.605	2.490	2,885 (0.045)	0.083	Yes
Enteritidis A	8	180 (0.83)	178	50	7	20.948	17.955	0 (0)	0.124	No
Infantis										
Infantis A	1-1	32 (1)	141	20	67	9.121	7.838	5 (0.035)	0.295	No
Infantis A	1-2	32 (0.97)	5,341	4,221	512	0.800	NC	259 (0.048)	0.158	Yes
Infantis A	1-3	32 (0.98)	12,479	9,471	1,136	0.349	NC	9,041 (0.724)	0.413	Yes
Infantis A	1-5	32 (0.95)	682	156	286	7.222	7.222	190 (0.279)	0.104	No
Kentucky										
Kentucky A	1	152 (0.92)	7,921	7,682	17	0.006	NC	429 (0.054)	0.114	Yes
Kentucky A	3	314 (0.98)	361	30	101	0.406	NC	112 (0.310)	0.370	No
Kentucky B	2	198 (0.98)	240	219	2	0.147	NC	8 (0.033)	0.074	Yes
Kentucky B	4	198 (0.93)	1,391	191	807	8.308	7.675	1,358 (0.976)	0.938	No
Montevideo										
Montevideo A	1	316 (0.98)	936	703	115	0.531	NC	18 (0.019)	0.695	No
Montevideo A	3	699 (0.97)	121	92	18	6.129	5.701	1 (0.008)	0.210	No
Montevideo A	6	4 (0.56); 195 (0.31)	1,727	1,452	144	2.188	2.060	68 (0.039)	0.138	No
Montevideo A	7	81 (0.97)	1,442	1,360	14	2.692	2.589	6 (0.004)	0.004	Yes
Montevideo A	10	138 (0.92)	3,211	3,054	53	0.171	NC	95 (0.030)	0.080	Yes
Reading										
Reading A	1-1-2	412 (1)	2,006	1,853	3	0.297	NC	566 (0.282)	0.971	Yes
Reading B	3-1-1	93 (0.97)	284	175	70	5.330	3.718	30 (0.106)	0.135	No
Reading C	1-1-4	1628 (0.87)	283	250	1	0.112	NC	70 (0.247)	0.291	Yes

^a^ Sequence type (ST) was determined using the in silico multilocus sequencing typing. For a given clade, the most common ST(s) and proportion(s) were determined based on representative isolates from each of the SNP clusters and all singletons in that clade.

^b^ The total number of isolates account for isolates collected worldwide. This number does not necessarily equal the sum of UK and US isolates.

^c^ The odds ratios (OR) of human isolates were calculated based on isolates collected from the United States or the United Kingdom, whichever represented the predominant country. All OR > 1.0 had a corresponding adjusted *P*-value < 0.01.

^d^ Outliers possibly representing human outbreak strains were removed and new OR were calculated as described in Materials and Methods. All OR > 1.0 had a corresponding adjusted *P*-value < 0.01.

^e^ The number and proportion of multidrug-resistant (MDR) isolates of a given clade were determined based on all isolates within the clade.

^f^ NC, not calculated.

**TABLE 3:** Key evolutionary parameters of the 12 clades associated with animal or environmental sources

Phylogenetic group	Clade	Isolation source	Mean TMRCA in years (95% HPD interval)^*a*^	Mean evolutionary rate in substitutions/site/year (95% HPD interval)^*b*^	Estimated emergence year^*c*^
Cerro					
Cerro A	2	Cattle	114.4 (112.0–119.9)	3.5 × 10^−7^ (2.9 × 10^−7^–4.0 × 10^−7^)	1907
Cerro A	3	Swine	24.9 (17.0–36.3)	3.7 × 10^−7^ (2.7 × 10^−7^–4.8 × 10^−7^)	1996
Dublin					
Dublin	2-3	Cattle	136.7 (97.0–184.1)	1.5 × 10^−7^ (1.1 × 10^−7^–1.9 × 10^−7^)	1884
Enteritidis					
Enteritidis A	7	Chicken	388.3 (284.4–505.2)	2.1 × 10^−7^ (1.8 × 10^−7^–2.4 × 10^−7^)	1633
Infantis					
Infantis A	1-2	Swine (OR = 4.8)	70.9 (54.4–89.9)	1.8 × 10^−7^ (1.3 × 10^−7^–2.3 × 10^−7^)	1950
Infantis A	1-3	Chicken	88.4 (62.1–119.4)	1.9 × 10^−7^ (1.4 × 10^−7^–2.4 × 10^−7^)	1933
Kentucky					
Kentucky A	1	Chicken	44.7 (36.1–54.4)	4.7 × 10^−7^ (4.0 × 10^−7^–5.5 × 10^−7^)	1976
Kentucky B	2	Cattle	65.4 (43.6–92.0)	1.7 × 10^−7^ (1.1 × 10^−7^–2.2 × 10^−7^)	1956
Montevideo					
Montevideo A	7	Other	3764.6 (947.0–8090.9)	8.1 × 10^−9^ (1.9 × 10^−9^–1.6 × 10^−8^)	1744 BCE
Montevideo A	10	Cattle	115.8 (63.5–180.4)	1.9 × 10^−7^ (1.3 × 10^−7^–2.5 × 10^−7^)	1905
Reading					
Reading A	1-1-2	Turkey	33.6 (26.1–42.0)	2.6 × 10^−7^ (2.0 × 10^−7^–3.2 × 10^−7^)	1987
Reading C	1-1-4	Swine	48.4 (35.8–62.7)	2.1 × 10^−7^ (1.7 × 10^−7^–2.6 × 10^−7^)	1973

^a^ The mean and 95% highest posterior density (HPD) of time to a most recent common ancestor (TMRCA) were inferred from the TreeHeight parameter estimates reported by Tracer.

^b^ The mean and 95% highest posterior density (HPD) of evolutionary rate were inferred from the parameter "rate.mean", estimated by Tracer.

^c^ The estimated emergence year of a given clade was calculated by subtracting the mean TMRCA from 2021, the collection year of the most recent isolate in the analysis.

The clonality level of the 25 clades varied widely even within serovars, with the clonal index (CI) ranging from 0.004 for Montevideo-A-7 (environment-associated; lowest clonality, highest genetic diversity) to 0.971 for Reading-A-1-1-2 (turkey-associated; highest clonality, lowest genetic diversity; Table 2). Four other clades also showed CI >0.500, namely Kentucky-B-4 (no source association; CI = 0.938), Cerro-A-3 (swine-associated; CI = 0.762), Dublin-2-3 (cattle-associated; CI = 0.742), and Montevideo-A-1 (cattle-associated; CI = 0.695).

The analysis of the clades identified 11 clades with enrichment for human isolates (OR >2.0; *P*-value < 0.05), including Montevideo-A-7 (environment-associated) and Enteritidis-A-7 (chicken-associated) as well as nine clades not associated with any source categories (Table 2). Most human-enriched clades exhibited a high genetic diversity with a CI up to 0.295 (Infantis-A-1-1), except for Kentucky-B-4, which exhibited a CI of 0.938.

For three clades, over 50% of isolates carried AMR determinants conferring resistance to three or more drug classes (thus considered MDR; Table 2; see Data Set S2 for the AMR genotype of all isolates within each serovar/phylogenetic group). Among these, two clades were source-associated and represented the largest clades within their respective serovars: Dublin-2-3 (89% MDR; cattle-associated; containing 66% of *S*. Dublin isolates) and Infantis-A-1-3 (72% MDR; chicken-associated; containing 66% of *S*. Infantis isolates). The highest proportion of MDR isolates (98%) was found in Kentucky-B-4 (no source association).

### Characterization of public health significance and time of emergence of *Salmonella* clades that show differential association with animal or environmental sources

**Fig 3:**
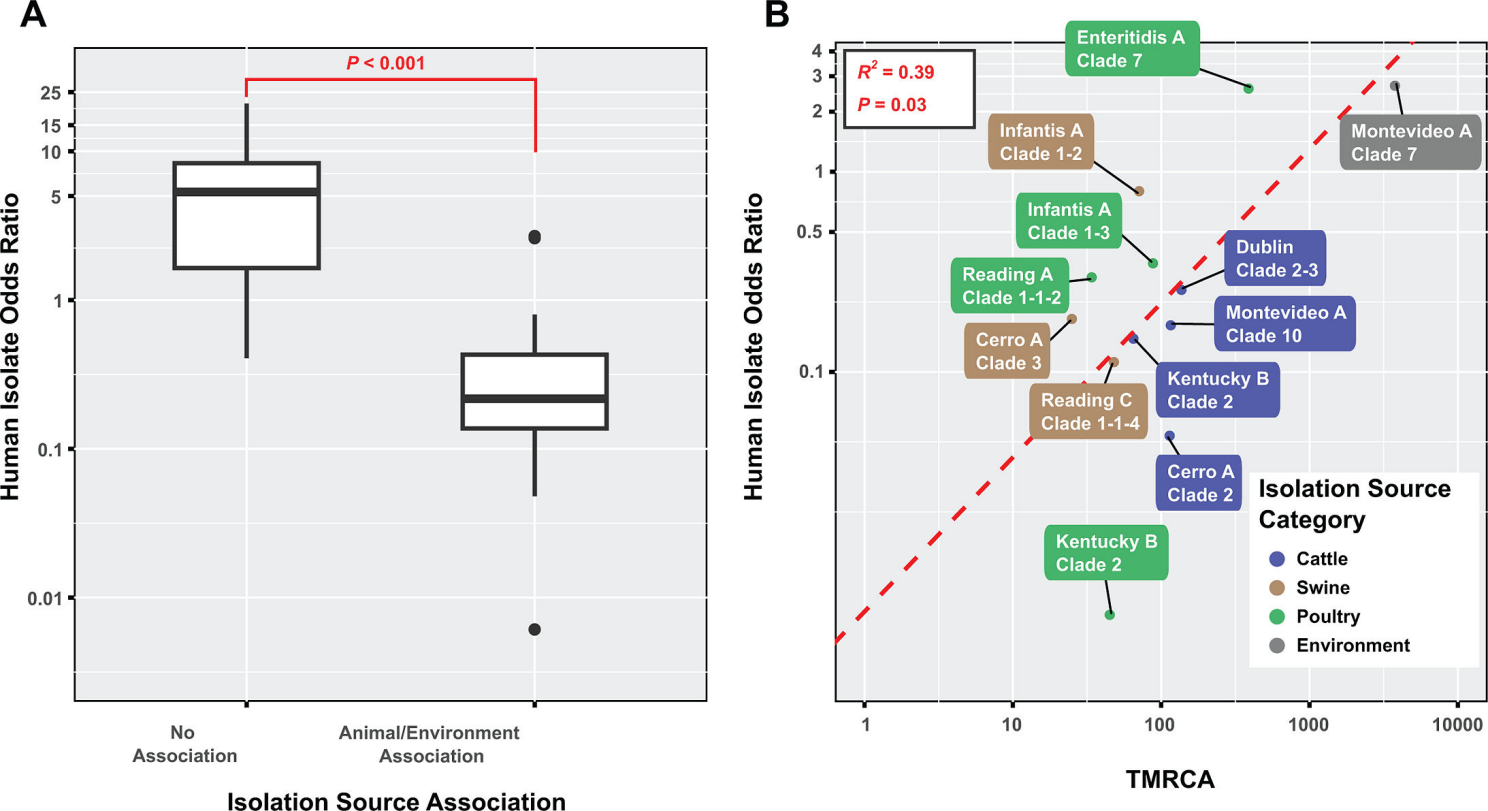
Potential factors associated with the odds ratio between human and non-human isolates within a given clade. (**A**) Box plot showing that association with animal or environmental sources (x-axis) corresponded to a reduction in the odds ratio between human and non-human isolates (y-axis). (**B**) X-Y plot showing that TMRCA (x-axis) had a positive association with the odds ratio between human and non-human isolates among animal- or environment-associated clades (y-axis). The red, dashed line represents the regression line returned by the corresponding linear regression model. Clades are color-coded according to the isolation category found to be enriched in each clade.

Clades not associated with specific source categories showed significantly (*P* < 0.001) higher human isolation odds ratios as compared to clades associated with animal or environmental sources, suggesting an enhanced likelihood of causing human illnesses for strains capable of circulating among multiple sources (Fig. 3A). Alternatively, this association could be due to oversampling from sources (e.g., chickens) linked to source-associated clades. Among animal- or environment-associated clades, a significant (*P* = 0.03) positive correlation was found between the time to the most recent common ancestor (TMRCA) and human isolation odds ratio, indicating clades of earlier origin tend to have greater public health significance compared to more recently emerged clades (Fig. 3B).

### Comparison of substitution rate of *Salmonella* isolates adapted to distinct environment- or animal-related niches

While the average evolutionary rate among animal-associated clades was 2.5 × 10^−7^ substitutions/site/year (equivalent to accumulation of 1.3 SNPs per year), the environment-associated clade Montevideo-A-7 had a 30 times lower estimated evolutionary rate (8.1 × 10^−9^ substitutions/site/year; 0.04 SNPs per year) (Table 3). The evolutionary rate of Montevideo-A-7 was also more than 23 times lower than that of the phylogenetically related cattle-associated clade Montevideo-A-10, suggesting that *S.* Montevideo isolates adapted to natural environments (e.g., water, sediments) have a generation time (i.e., the average time taken for bacterial numbers to double) 23 times longer than *S.* Montevideo isolates adapted to cattle-related sources, assuming similar mutation rates. Among animal-associated clades, cattle-associated clades tended to show lower evolutionary rates than those associated with other animal sources; three of the four clades with the lowest evolutionary rates were associated with cattle-related sources (Dublin-2-3: 1.5 × 10^−7^; Kentucky-B-2: 1.7 × 10^−7^; Montevideo-A-10: 1.9 × 10^−7^). Clades associated with chicken (*n* = 3), turkey (*n* = 1), and swine (*n* = 3) exhibited, on average, approximately 1.6 times higher evolutionary rates than these three cattle-associated clades. Two *S.* Cerro clades, Cerro-A-2 (cattle-associated) and Cerro-A-3 (swine-associated), exhibited comparable high evolutionary rates of 3.5 × 10^−7^ and 3.7 × 10^−7^, respectively, suggesting *S.* Cerro associated with cattle may have a naturally higher mutation rate or have adapted to cattle and/or cattle-related environments (e.g., cattle farms) in a manner that allows for faster replication compared to other cattle-associated serovars. Although the difference in evolutionary rate among clades associated with different source categories was not significant due to the relatively small sample size, our results suggest that evolutionary rates may be impacted by niche association.

### Emergence and evolutionary characteristics of the *S*. Montevideo clade associated with environmental sources

**Fig 4:**
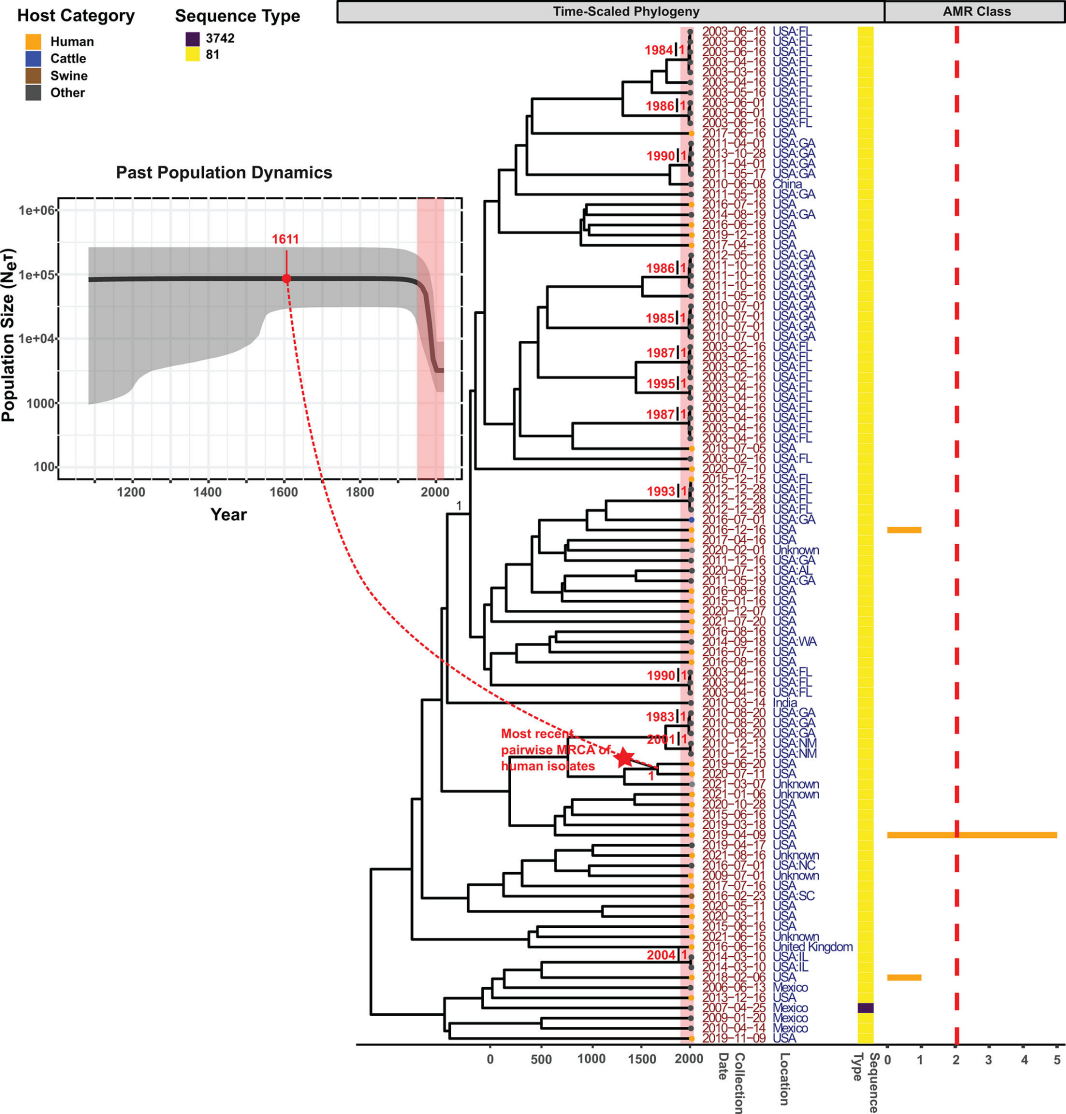
Time-scaled phylogeny (with BSP insert) for Montevideo A Clade 7. The Bayesian time-scaled maximum clade credibility (MCC) phylogeny and BSP were reconstructed based on core SNPs identified among 100 representative isolates within Montevideo-A-7; representative isolates were selected using time-stratified random sampling. The embedded BSP depicts the dynamics of the effective population size over time, with the y-axis representing the product of effective population size (***N****e*) and generation time (***τ***) and the x-axis representing time in years. A bold line is used to display the median of ***Neτ*** across the complete time course; the shaded area surrounding the line represents the 95% highest posterior density (HPD) interval. The MCC phylogeny next to the BSP represents time in years on the x-axis, with branch lengths reported in years. The red star indicates the most recent common ancestor between two human isolates, with the corresponding posterior probability of branch support next to it. Leaf nodes are color-coded to denote the isolation source category of the representative isolates, with the category “other” including isolates not collected from sources related to humans, cattle, swine, chicken, and turkey. The following information is displayed adjacent to the leaf nodes: (i) collection date, (ii) geographical location, and (iii) sequence type. The bar plot on the far-right displays, for each representative isolate, the number of antimicrobial resistance (AMR) classes, based on the presence of AMR determinants in the genome. Bars color-match the leaf nodes, and a vertical red dashed line is overlaid to distinguish multidrug-resistant (MDR) isolates (i.e., isolates resistant to at least one agent in at least three drug classes) from the rest. A red rectangle is overlaid on both the MCC phylogeny and the BSP to highlight the period in which a substantial reduction in ***Neτ*** was observed. Clusters of closely related environmental isolates are labeled with the date and posterior probability of branch support at the ancestral nodes (see the text for details).

The time-scaled phylogeny of the environment-associated Montevideo-A-7 clade showed an estimated TMRCA of 3,765 years (Table 3), indicating isolates from this clade shared an MRCA that existed around 1744 BCE, far earlier than the origin of any animal-associated clade studied here. We did not identify any sub-clades with distinct characteristics that have emerged after the MRCA of Montevideo-A-7 (Fig. 4). As the shortest pairwise TMRCA among human Montevideo-A-7 was 410 years, our results suggest that isolates within this clade have only caused sporadic cases, although human isolates constituted 86% of Montevideo-A-7 isolates. The Bayesian Skyline Plot (BSP), which depicts changes in the effective population size (*Ne*) and generation time (*τ*) (43) over time, showed a constant *Neτ* for Montevideo-A-7 until the 1950s, followed by a sharp decline. However, as the representative isolates included several clusters of non-human isolates with comparable geographic locations and collection dates, this *Neτ* decline after the 1950s likely reflected a sampling bias leading to the inclusion of multiple environmental isolates genetically closely related, rather than a genuine reduction in *Neτ*.

### Emergence and evolution of Dublin-2-3 and Reading-A-1-1-2: identification of clonal sub-clades characterized by an enrichment of human isolates

For the 11 animal-associated clades, the time-scaled phylogenies were examined further to identify clonal sub-clades that exclusively comprised isolates with a maximum pairwise SNP distance <5% of the total SNPs (among the 100 representative isolates within the clade); these sub-clades were expected to display distinct evolutionary and population genetics characteristics. Among the 11 animal-associated clades, 21 clonal sub-clades were identified (Table 4). Notably, two clades (Dublin-2-3 and Reading-A-1-1-2) contained clonal sub-clades enriched for human isolates.

**TABLE 4:** Clonal sub-clades identified from within clades associated with animal source categories

Sub-clade^*a*^	Number of isolates	Emergence year	Percentage point change in the proportions of isolates compared to baseline^*b*^								
			Human	Cattle	Swine	Chicken	Turkey	Other	AMR^*c*^	MDR^*d*^	
Clades with sub-clades enriched for human isolates											
*Dublin clade 2-3*											
I	68	1977	**+27**	–18	NA^*e*^	+2	NA	–8	**+53**	**+66**	
*Reading A clade 1-1-2*											
I	27	2009	**+34**	NA	NA	–2	–29	–3	**+29**	+19	
II	14	1999	–29	NA	NA	+13	+20^*f*^	–3	+2	–25	
Clades with sub-clades enriched for animal-associated isolates that correspond with increases in Ne											
*Enteritidis A clade 7*											
I	24	1940	–32	NA	NA	**+38**	NA	+3	–51	–51	
II	11	1937	–55	+9	NA	**+36**	NA	+18	–46	–55	
III	40	1975	+3	NA	NA	+5	NA	–7	–8	–45	
IV	14	1926	+11	NA	NA	NA	NA	–2	–55	–55	
*Infantis A clade 1-3*											
I	32	1990	–29	NA	–5	**+40**	NA	–7	**+39**	**+39**	
II	19	1977	–5	+5	–3	–11	NA	+16	–40	–40	
*Cerro A clade 2*											
I	33	1996	–18	**+31**	+2	–2	NA	–10	–6	NA	
Clades with sub-clades enriched for animal-associated isolates that do not correspond with increases in Ne											
*Reading C clade 1-1-4*											
I	30	2010	+5	–55	**+52**	NA	NA	+1	0	–62	
II	20	2002	–7	–52	**+72**	NA	NA	–11	0	–62	
*Cerro A clade 3*											
I	55	2011	–7	+1	**+33**	–9	NA	–12	–11	–4	
*Kentucky B clade 2*											
I	26	2013	–8	–56	NA	–3	NA	**+70**	–33	–2	
II	13	1985	–12	–56	NA	+12	**+70**	–14	**+44**	+13	
*Montevideo A clade 10*											
I	29	1968	–0	**+20**	NA	–2	+1	–16	+11	+5	
II	23	1958	–1	**+28**	NA	–2	–2	–20	**+21**	–2	
III	17	1979	–13	+4	+12	+3	–2	–2	**+21**	+3	
Clades containing no sub-clades enriched for human- or animal-associated isolates											
*Infantis A clade 1-2*											
I	31	1976	–0	–3	+13	–3	–1	–6	+0	–6	
*Kentucky A clade 1*											
I	45	1996	–6	NA	NA	+15	NA	–7	**+62**	+14	
II	21	2003	–6	NA	NA	+18	NA	–9	**+62**	–1	

^*a*^ Based on the time-scaled phylogeny reconstructed for a given clade, clonal sub-clades were defined as clusters of isolates that (i) account for >10% representative isolates, (ii) show a posterior probability of >0.8, and (iii) a maximum pairwise genetic distance less than 5% of the total number of core SNPs used for reconstructing the time-scaled phylogeny.

^*b*^ All percentages were calculated based on the representative isolates used for reconstructing the time-scaled phylogeny. Baseline percentages were calculated using isolates that do not belong to any clonal sub-clades. Percentage point increases over 20 are highlighted in bold and underlined.

^*c*^ Isolates harboring AMR determinants to at least one drug class are considered as AMR isolates.

^*d*^ Isolates harboring AMR determinants responsible for at least three drug classes are considered as MDR isolates.

^*e*^ NA: Not applicable. Both sub-clade and baseline contain no isolates for the respective group (e.g., chicken source, AMR, MDR).

^*f*^ Before rounding, Reading-A-1-1-2-II had a percentage point change of 19.6, and therefore, was not considered enriched for turkey isolates based on the 20-percentage point increase in comparison to baseline threshold that we used to identify enriched sub-clades.

**Fig 5:**
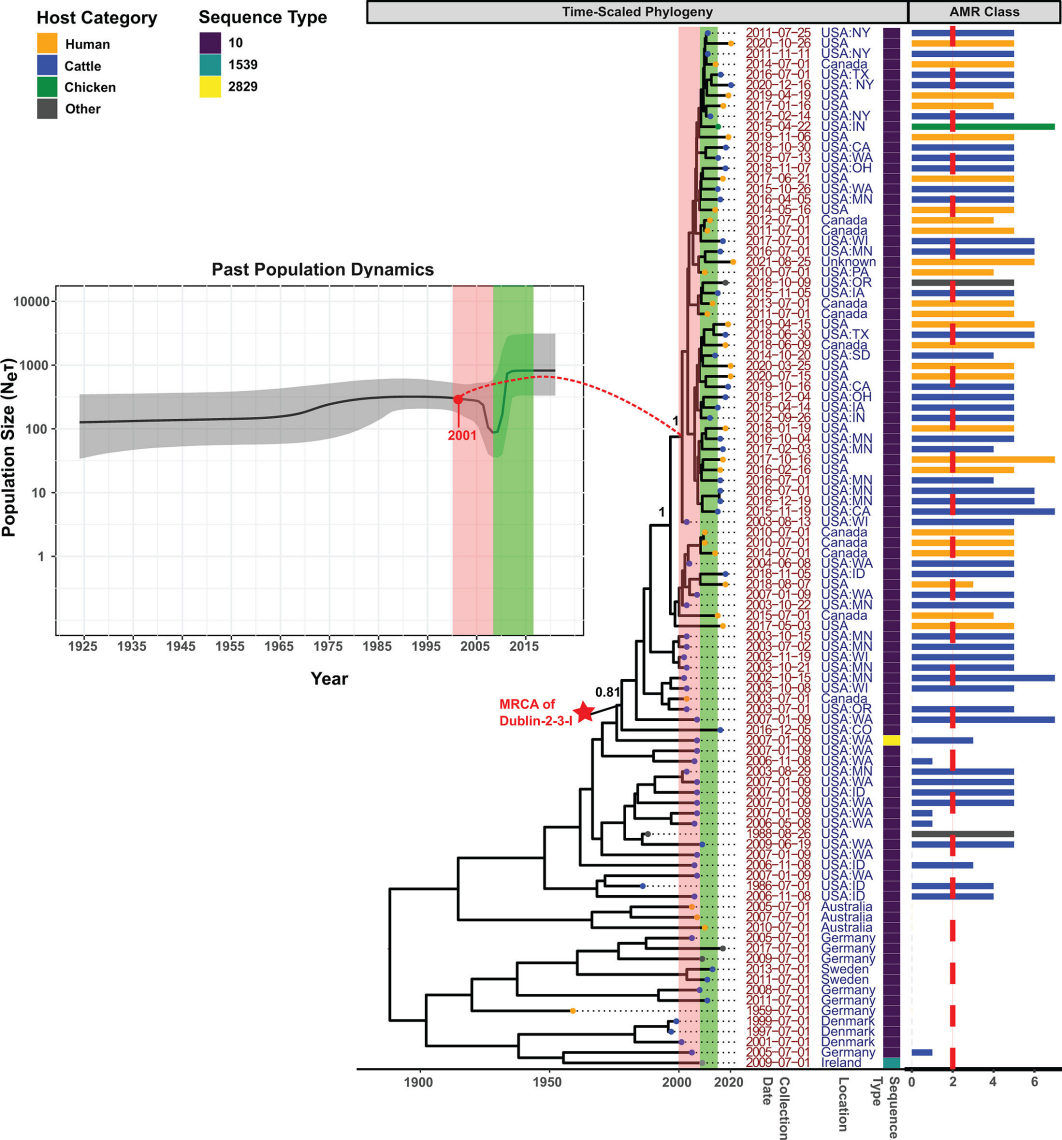
Time-scaled phylogeny (with BSP insert) for Dublin Clade 2-3. The Bayesian time-scaled maximum clade credibility (MCC) phylogeny and BSP were reconstructed based on core SNPs identified among 100 representative isolates within Dublin clade 2-3; representative isolates were selected using time-stratified random sampling. The embedded BSP depicts the dynamics of the effective population size over time, with the y-axis representing the product of effective population size (*Ne*) and generation time (***τ***) and the x-axis representing time in years. A bold line is used to display the median of *Neτ* across the complete time course; the shaded area surrounding the line represents the 95% highest posterior density (HPD) interval. The MCC phylogeny next to the BSP represents time in years on the x-axis, with branch lengths reported in years. The red star indicates the most recent common ancestor (MRCA) of sub-clade I, and posterior probabilities of branch support are attached to the MRCA along with two subsequent ancestors. Leaf nodes are color-coded to denote the isolation source category of the representative isolates, with the category “other” including isolates not collected from sources related to humans, cattle, swine, chicken, and turkey. The following information is displayed adjacent to the leaf nodes: (i) collection date, (ii) geographical location, and (iii) sequence type. The bar plot on the far-right displays, for each representative isolate, the number of antimicrobial resistance (AMR) classes, based on the presence of AMR determinants in the genome. Bars are color-matched with the leaf nodes, and a vertical red dashed line is overlaid to distinguish multidrug-resistant (MDR) isolates (i.e., isolates resistant to at least one agent in at least three drug classes) from the rest. Colored rectangles overlaid on both the MCC phylogeny and the BSP highlight important time blocks associated with the clonal expansion of sub-clade I (see text for details).

Dublin-2-3 (cattle-associated; estimated emergence in 1884) contained a sub-clade (Dublin-2-3-I; 68% of the isolates; emerged in 1977) within which 40% of the isolates were from humans, compared to 13% of the baseline isolates (i.e., isolates within the same clade, but not part of any clonal sub-clades) (Fig. 5). Most isolates within Dublin-2-3-I were collected in or after 2012 (71%), were MDR (97%), and were from North America (99%). Conversely, 100% of the baseline isolates were collected before 2012, 31% were MDR, and 50% were from North America. Furthermore, we observed an increase in the occurrence of short branches and coalescent events between the years 2000 and 2008 (Fig. 5, red time block), which are hallmarks of declining genetic diversity and that correspond well with the decline in *Neτ* shown in the BSP (Fig. 5). However, between 2009 and 2015, there is an increased number of long branches (Fig. 5, green time block), suggesting an increase in genetic diversity and corresponding well with the rise in *Neτ* shown in the BSP (Fig. 5). Therefore, this MDR, human-associated sub-clade appears to have experienced a recent clonal expansion (since around 2001) and has gradually become dominant within Dublin-2-3.

**Fig 6:**
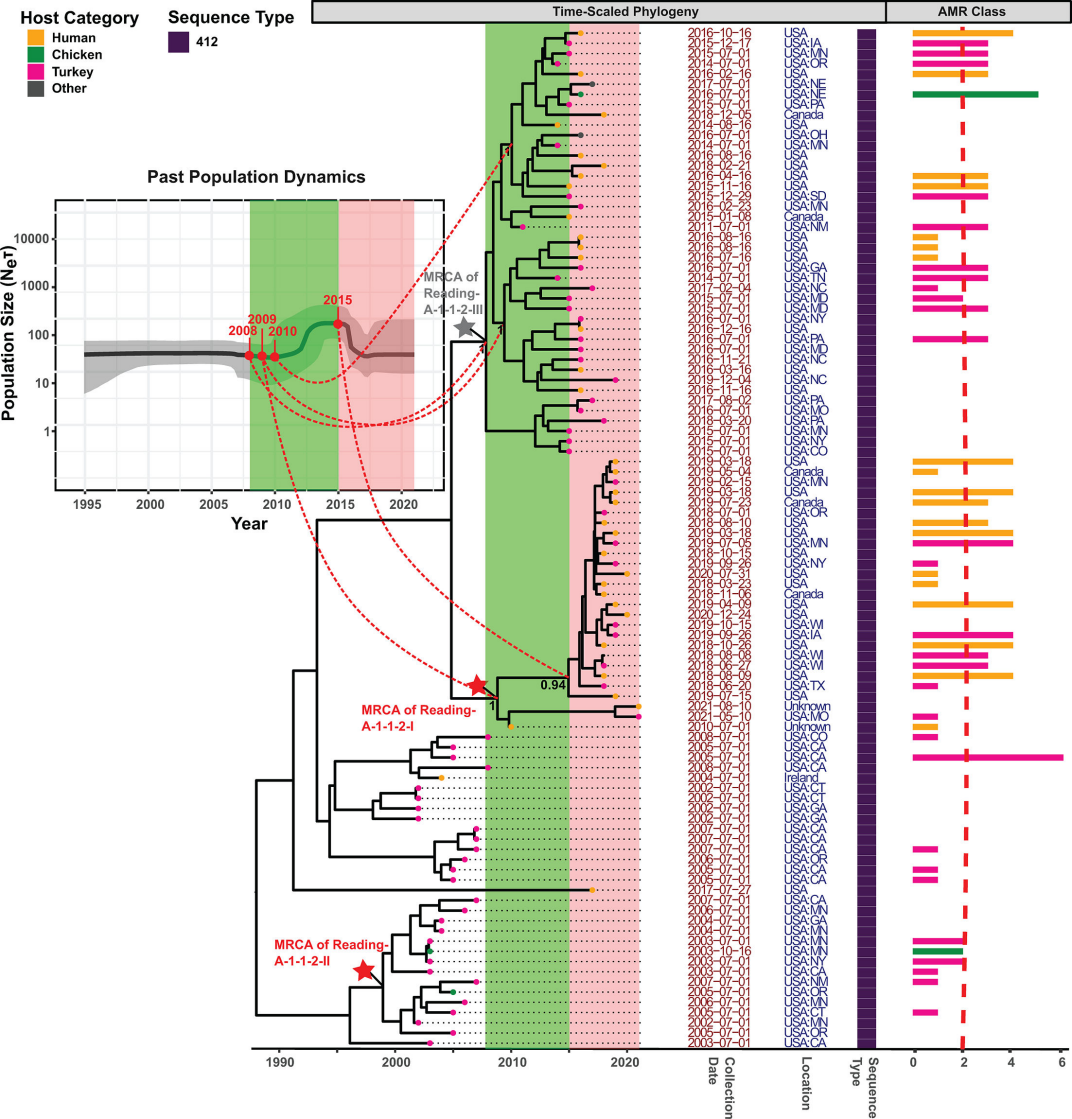
Time-scaled phylogeny (with BSP insert) for Reading A Clade 1-1-2. The Bayesian time-scaled maximum clade credibility (MCC) phylogeny and BSP were reconstructed based on core SNPs identified among 100 representative isolates within Reading A clade 1-1-2; representative isolates were selected using time-stratified random sampling. The embedded BSP depicts the dynamics of the effective population size over time, with the y-axis representing the product of effective population size (*Ne*) and generation time (*τ*) and the x-axis representing time in years. A bold line is used to display the median of *Neτ* across the complete time course; the shaded area surrounding the line represents the 95% highest posterior density (HPD) interval. The MCC phylogeny next to the BSP represents time in years on the x-axis; with branch lengths reported in years. Red stars indicate the most recent common ancestor (MRCA) of sub-clades I and II, and the gray star indicates the MRCA of a manually defined sub-clade III, which is highly diverse (see the text for details). Posterior probabilities of branch support are attached to the MRCAs as well as important subsequent ancestors. Leaf nodes are color-coded to denote the isolation source category of the representative isolates, with the category “other” including isolates not collected from sources related to humans, cattle, swine, chicken, and turkey. The following information is displayed adjacent to the leaf nodes: (i) collection date, (ii) geographical location, and (iii) sequence type. The bar plot on the far-right shows, for each representative isolate, the number of antimicrobial resistance (AMR) classes, based on the presence of AMR determinants in the genome. Bars are color-matched with the leaf nodes, and a vertical red dashed line is overlaid to distinguish multidrug-resistant (MDR) isolates (i.e., isolates resistant to at least one agent in at least three drug classes) from the rest. Colored rectangles are overlaid on both the MCC phylogeny and the BSP to highlight important time blocks that involve changes in *Neτ*.

Reading-A-1-1-2 (turkey-associated; estimated emergence in 1987) contained a sub-clade (Reading-A-1-1-2-I; 27% of the isolates; emerged in 2009) with a human isolate proportion of 63%, compared to 29% among baseline isolates (Fig. 6). A subset of 89% of isolates in Reading-A-1-1-2-I shared an ancestor with an estimated emergence date of 2015. Approximately 96% of the isolates within Reading-A-1-1-2-I were collected in or after 2018, while 95% of the other Reading A-1-1-2 isolates were collected in or before 2017. A second clonal sub-clade (Reading-A-1-1-2-II; 14% of the isolates; emerged in 1999) did not show any enrichment for human or animal sources (with enrichment defined as a proportional increase for a given category by >20 percentage points compared to the baseline level). We also manually identified an additional, genetically diverse sub-clade (Reading-A-1-1-2-III; 42% of the isolates; emerged in 2008) with 91% of isolates collected between 2012 and 2016 (as compared to 2% among isolates outside this sub-clade). The emergence of Reading-A-1-1-2-III corresponded with a notable rise in *Neτ* observed between 2008 and 2015 (Fig. 6, green time block), which was followed by a decline in *Neτ* between 2015 and 2021, likely driven by the emergence of Reading-A-1-1-2-I (Fig. 6, red time block). Therefore, our results suggest the emergence of a putative sub-clade (Reading-A-1-1-2-III) that gradually increased in genetic diversity and was replaced by Reading-A-1-1-2-I, which appeared to have an elevated public health significance and experienced a recent clonal expansion. Importantly, Reading-A-1-1-2-I and Reading-A-1-1-2-III accounted for a combined 96% (26/27) of the MDR isolates and 94% (32/34) of the human isolates in Reading-A-1-1-2, suggesting the involvement of AMR in the expansion of Reading A population.

### Emergence and evolution of Enteritidis-A-7, Infantis-A-1-3, and Cerro-A-2 sub-clades

Our analyses identified four clonal sub-clades within Enteritidis-A-7 (chicken-associated; estimated emergence in 1633). Two of these sub-clades, Enteritidis-A-7-I (24% of the isolates; emerged in 1940) and Enteritidis-A-7-II (11% of the isolates; emerged in 1937), showed an increased proportion of chicken isolates (38% and 36% inside Enteritidis-A-7-I and Enteritidis-A-7-II, respectively, compared to 0% among baseline isolates; Table 4; Fig. S17). In addition, Enteritidis-A-7-I has become increasingly prevalent, with 50% of its isolates collected in or after 2017, compared to 9% among baseline isolates. Importantly, the BSP of Enteritidis-A-7 suggested an increase in *Neτ* around the 1980s, which is right after the MRCA of an ubiquitous third clonal sub-clade (Enteritidis-A-7-III; 40% of the isolates; emerged in 1975) within which 85% of the isolates were collected from humans across different continents, suggesting a global spread of this sub-clade.

Infantis-A-1-3 (chicken-associated; estimated emergence in 1933) contained a clonal sub-clade (Infantis-A-1-3-I; 32% of the isolates; emerged in 1990) that showed an increase in the proportion of chicken-associated isolates (56% within sub-clade compared to 16% among baseline isolates; Fig. S18). The BSP for Infantis-A-1-3 indicated a decline in *Neτ* followed by a rapid *Neτ* increase around 2005–2010. Within Infantis-A-1-3-I, a node dated to year 2005 represented the TMRCA of 18% of the isolates in Infantis-A-1-3. All descendants from this node were obtained in the United States in or after 2015 and were MDR (100% of the 18 isolates compared to 59% of the remaining 82 isolates in Infantis-A-1-3). Moreover, these 18 isolates include 92% (12/13) of the chicken isolates from the United States in Infantis-A-1-3, suggesting an expansion of an MDR clonal Infantis A subtype within US chicken flocks since 2005.

Cerro-A-2 (cattle-associated; estimated emergence in 1907) contained a clonal sub-clade (Cerro-A-2-I; 33% of the isolates; emerged in 1996) that showed a notably higher proportion (67%) of cattle-associated isolates than that (36%) among baseline isolates (Fig. S19). The emergence and the subsequent clonal expansion of this sub-clade appear to be associated with an increase in *Neτ* of Cerro-A-2 between 2000 and 2010.

### Emergence and evolution of Reading-C-1-1-4, Cerro-A-3, Kentucky-B-2, and Montevideo-A-10 sub-clades

Reading-C-1-1-4 (swine-associated; estimated emergence in 1973) contained two clonal sub-clades, Reading-C-1-1-4-I (30% of the isolates; emerged in 2010) and Reading-C-1-1-4-II (20% of the isolates; emerged in 2002); 60% and 80% of isolates in these two sub-clades were obtained from swine (as compared to 8% among baseline isolates) (Fig. S20). Both Reading-C-1-1-4-I and Reading-C-1-1-4-II appeared to have experienced recent clonal expansion, which may have contributed to a slight decline in *Neτ*. While 95% of the isolates from Reading-C-1-1-4-II were collected before 2019, 90% of the isolates from Reading-C-1-1-4-I were collected in or after 2019, consistent with the later MRCA for Reading-C-1-1-4-I. Our results thus suggest a replacement of clonal sub-clades among Reading-C-1-1-4 isolates associated with swine sources, which may have caused a prolonged decline in *Neτ*.

Cerro-A-3 (swine-associated; emerged in 1996) contained a clonal sub-clade (Cerro-A-3-I; 55% of the isolates; estimated emergence in 2011) that showed an increased proportion of swine-associated isolates (71% within Cerro-A-3-I as compared to 38% among baseline isolates; Fig. S21).

Montevideo-A-10 (cattle-associated; estimated emergence in 1905) contained two sub-clades, Montevideo-A-10-I (29% of the isolates; estimated emergence in 1968) and Montevideo-A-10-II (13% of the isolates; estimated emergence in 1958), that exhibited a rise in the proportion of cattle-associated isolates (from 49% among baseline isolates to 69% and 77% in Montevideo-A-10-I and Montevideo-A-10-II, respectively; Fig. S22).

Although found to be associated with cattle, Kentucky-B-2 (estimated emergence in 1956) contained two sub-clades, Kentucky-B-2-I (26% of the isolates; estimated emergence in 2013) and Kentucky-B-2-II (13% of the isolates; emerged in 1985), which appeared to be enriched for isolates from other sources or turkey, respectively (Fig. S23). Kentucky-B-2-I had 88% of its isolates collected from alfalfa between February and April of 2016, primarily in the US states of California, Kansas, and Tennessee, and was therefore presumed to represent the outcome of an investigation into the alfalfa seed multi-state outbreak in 2015–2016 (https://www.cdc.gov/salmonella/muenchen-02-16/index.html). Accordingly, the decrease in *Neτ* observed in the BSP for Kentucky-B-2 was likely caused by a sampling bias due to an enhanced sampling of closely related isolates from alfalfa in response to the outbreak. Kentucky-B-2-II, however, exhibited a rise in the proportion of turkey isolates (69%) in contrast to the baseline isolates (0%), which appeared to reflect neither a sampling bias nor a clonal expansion.

## DISCUSSION

Assembly and comprehensive analysis of a large set of WGS data for seven *Salmonella* serovars associated with agricultural animals in the United States allowed to provide new insights into the evolution and population genetics of key *Salmonella* serovars. Initial analyses showed that each serovar contained distinct clades, including some that showed strong association with isolation from human clinical cases, different agricultural animals, and environmental sources. Subsequent analyses showed that *Salmonella* generation time is associated with source with an environmental *Salmonella* Montevideo clade showing substantially longer generation times than any other clade, which has important implications for both our understanding of *Salmonella* evolution and interpretation of genetic differences in outbreak investigations. Finally, we found that all serovars contain clades and sub-clades that emerged since 1937 and were enriched for isolates associated with humans, animals, AMR, and/or geographical regions. Some of these sub-clades appear to represent groups of particular public health and/or animal health concerns, which could be targeted for specific interventions. These findings show that the recently introduced concept of REP strains (https://www.cdc.gov/foodborne-outbreaks/php/rep-surveillance/index.html), which represent reoccurring, emerging, and persisting strains, has broad applicability and illustrates a pathway for more target approaches to addressing human and animal infectious disease prevention.

### Phylogenetic analysis using WGS data and their respective metadata available in public repositories provided supporting evidence for associations between clades within serovars and different source categories

Although several *Salmonella* serovars have been associated with specific sources (e.g., *S*. Reading and turkey, *S*. Enteritidis and chicken), the association of specific clades within serovars and sources has not been widely described. In this study, 12 clades were found to be associated with specific source categories, including four cattle-associated clades, three chicken-associated clades, one turkey-associated clade, three swine-associated clade, and one environment-associated clades. While a number of these clades appear to represent previously described clade/source associations (44–49), some were newly described here. Among those that were previously described, Reading-A-1-1-2 and Reading-C-1-1-4 correspond to clades 1 and 3, previously described by Miller et al. (48) as being associated with turkey and swine/bovine sources, respectively; Montevideo-A-10 and Montevideo-A-7 correspond to clades I and III, previously described by Nguyen et al. (46) as being associated with cattle and water/human sources, respectively; Cerro-A-2 corresponds to the large clonal clade, previously described by Cohn et al. (44) as being associated with cattle sources; Kentucky-B-2 corresponds to clade 198.1, previously described by Haley et al. (50), as being associated with cattle sources; Kentucky-A-1 corresponds to ST 152, previously described as being associated with poultry and dairy cattle sources in (49, 50); and Infantis-A-1-3 includes the pESI-like megaplasmid-carrying subtype, previously described by McMillan et al. (51), as being associated with US poultry sources in recent years. Importantly, however, these previous associations had not necessarily been supported statistically, due in part to limitations imposed by the sampling scheme and/or sample size of different studies. For example, Haley et al. (50) identified two distinct clusters within *S.* Kentucky ST 198. Cluster 198.2 corresponded with an epidemic MDR clone that emerged in Egypt in the 1990s and subsequently spread across continents through the potential transmission by poultry products (52–54), while cluster 198.1 comprised only five isolates collected from domestic cattle and food products. Here, we analyzed all *S.* Kentucky data available in the NCBI PD database, which led to the identification of two major ST 198 clades (Kentucky-B-2 and Kentucky-B-4) that contrasted in their human association, number of AMR determinants, clonality level, geographic association, and host association. Our findings thus emphasize the necessity of monitoring specific clades [https://www.cdc.gov/foodborne-outbreaks/php/rep-surveillance/index.html (55)] and highlight the importance of applying systematic approaches to more comprehensively characterize the phylogeny of a given serovar, taking advantages of the vast WGS data and associated metadata accessible through public databases.

### While *Salmonella* from clades associated with animal sources showed generation times more than 30 times shorter than those from environmental sources, *Salmonella* from cattle-associated clades typically had a longer generation time than *Salmonella* from swine- or poultry-associated clades

As WGS has been routinely incorporated in the surveillance of *Salmonella*, clustering based on genetic (e.g., SNP or allelic) differences has become the gold standard for subtyping and source tracking during outbreak investigations (56). To achieve optimal clustering results, the SNP or allelic difference thresholds used for defining clusters should take into consideration the generation time/substitution rate of the pathogen and/or their environmental settings (57, 58). Here, we reported an estimated substitution rate of 8.1 × 10^−9^ substitutions/site/year for the Montevideo-A-7 clade, which is putatively adapted to environmental sources; this substitution rate was more than 30 times lower than the average substitution rate of clades putatively adapted to animal-related sources. Based on the underlying premise of constant mutation rate, this indicates the generation time is considerably shorter for *Salmonella* from animal-related sources as compared to *Salmonella* from environmental sources. Similarly, it has been previously reported that the generation time of *Salmonella enterica* and *Escherichia coli* was 50 and 45 times longer in the wild as compared to laboratory environments, respectively, and that the growth in non-animal environments would be expected to be much slower than in animal intestinal tracts for both organisms (59).

Further comparison of the estimated substitution rate between clades associated with different animals revealed a generally lower substitution rate (and thus higher generation time) for cattle-associated clades as compared to swine- or poultry-associated clades, suggesting reduced replication and/or transmission of *Salmonella* in cattle-related sources. In the United States, one of the striking differences between farms raising cattle and other agricultural animals is related to animal density. According to the 2017 Census of Agriculture, the average number of animal heads per farm was 106 for cattle, 1,089 for swine, and 1,584 for poultry (60), suggesting that cattle farms had a much lower animal density than swine or poultry farms. We hypothesize that the lower population density of cattle farms compared to swine and chicken farms results in a less frequent transmission between animals, thus reducing the replication rate of *Salmonella* in cattle farms. Previous studies have provided some evidence supporting the effect of herd size and stocking density on promoting *Salmonella* infection in different agricultural animals. For instance, Cummings et al. investigated *Salmonella* infection in 831 US cattle herds and identified herd size as a potential predictor for salmonellosis incidence (61). Studies of laying-hen flocks in European countries also indicated a positive correlation between *Salmonella* contamination and flock size (62, 63). Furthermore, high stocking density has been reported to contribute to *Salmonella* infection in both laying hens (64) and finisher pigs (65), possibly through facilitating intestinal colonization and fecal shedding of the pathogen, along with enhancing direct contact between animals and feces.

Notably, however, not all cattle-associated clades displayed comparable substitution rates (per year). While three of the four clades with the lowest substitution rates were cattle-associated (Dublin-2-3, Montevideo-A-10, and Kentucky-B-2), the substitution rate of Cerro-A-2 ranked third among all animal-associated clades. Previous studies have provided insights into potential mechanisms that may have contributed to the high estimated substitution rate of Cerro-A-2, such as reduced virulence to cattle, leading to more generations inside the host, extended shedding, and potential higher animal-to-animal transmission as compared to other serovars (e.g., *S.* Dublin) in cattle-related sources.

Importantly, our data show that clades of the same serovar, but associated with different sources, can have very different substitution rates, presumably due to differences in generation times. Sharp discrepancies in generation time may lead to differential rates of accumulating point mutations, among other types of genetic variants, thereby highlighting the importance of setting dynamic, source-specific thresholds with respect to SNP and/or allelic differences for more accurate source tracking during outbreak investigations (66).

### Evolution of *Salmonella* serovars associated with animals involved the emergence and occasional expansion of clonal groups

Among the 11 animal-associated clades we identified here, seven contained clonal sub-clades enriched for isolates collected from the corresponding animal-related sources, suggesting clonal expansion possibly driven by adaptation of the clonal group to a specific host and subsequent expansion of that clonal group population. Various factors may mediate this process, such as natural selection, driven by, for example, the acquisition of AMR determinants (67) or virulence factors (68). For instance, the application of antimicrobials in agricultural animal settings may select for *Salmonella* that show AMR (69).

In our study, clonal expansions of sub-clades within Dublin-2-3, Cerro-A-2, Enteritidis-A-7, and Infantis-A-1-3 were each followed by an increase in the *Neτ* of the corresponding clade, suggesting an increase in genetic diversity and/or population size. The *Neτ* increase within Dublin-2-3 and Infantis-A-1-3 was likely attributed to the acquisition of AMR determinants. Moreover, the identification, within Dublin-2-3, of an MDR sub-clade that predominantly included isolates from North America is consistent with previous findings that Dublin isolates carrying AMR genes were almost exclusively collected from the United States (70). Infantis-A-1-3 experienced a decline followed by an increase in *Neτ* between 2005 and 2010, which aligns with the emergence and diversification of a chicken-associated clonal sub-clade identified within this clade. Importantly, this sub-clade appears to contain the MDR emergent poultry-associated *S.* Infantis (ESI) clone carrying a megaplasmid (pESI), which was initially reported in Israel in 2014 and is spreading throughout the United States (71). The emergence of this *S*. Infantis clone has resulted in a sharp increase in poultry-associated *S*. Infantis since 2015 in the United States. From 1998 to 2008, *S.* Infantis was predominantly associated with pork and was not frequently isolated from poultry sources in the United States (11). Furthermore, *S*. Infantis accounted for <8% of *Salmonella* isolates from US domestic chicken samples collected in 2015–2016, while this serovar represented >30% of the isolates in 2022 (72). This recent increase in the prevalence of *S*. Infantis in chicken is consistent with our results showing that most *S*. Infantis isolates from US chicken-related sources were collected after 2008 and that there was a rise in *Neτ* for the *S*. Infantis chicken-associated clade around 2015.

The observed increase in *Neτ* among Cerro-A-2 and Enteritidis-A-7 does not seem to be related to the acquisition of AMR determinants. Two chicken-associated clonal sub-clades identified within Enteritidis-A-7 dated back to around 1940 but both expanded after 1975, when a sharp increase in *Neτ* was observed for Enteritidis-A-7. Our findings are consistent with the proposed hypothesis that the elimination of *S.* Gallinarum from poultry flocks by the 1970s created an open niche that allowed *S.* Enteritidis to thrive and ultimately become the most frequent *Salmonella* serovar in the United States by 1994, following a rapid increase in human infections around mid-1970s (73). Prior to the 1970s, however, *S*. Enteritidis might have been excluded from poultry flocks by *S.* Gallinarum, which generated immunity against the O_9_ antigen shared by these two serotypes (74).

Overall, our findings indicate that continued efforts are needed to understand *Salmonella* evolution at or below serotype level. While *Salmonella* serotyping is considered a standard subtyping method (56), our results suggest that most NTS serovars associated with major agricultural animals are polyphyletic. Consequently, subtypes of the same serovar may have different evolutionary histories and show distinct geographic distributions, source associations, pathogenic potential, and public health significance. In addition, our results highlight the importance of incorporating source-specific thresholds of SNP or allelic differences during source tracking of *Salmonella* outbreak investigations, as isolates from non-animal environments may require a longer time to accumulate SNPs relative to those from agricultural animals. Finally, we showed that several serovars have become associated with agricultural animals in the past century, with most of them showing an association that dates back only a few decades. Our findings suggest that these associations are primarily due to the expansion of clonal subtypes that have likely adapted to a given host animal and its environments. Although resistance to antimicrobials may play an important role in this adaptation, other factors, such as displacement or elimination of an established serovar from a niche, opening this niche to another serovar, and loss-of-virulence towards the animal host, have likely also contributed.

## SUPPLEMENTAL MATERIAL

Data Set S1 mbio.01913-24-s0001.xlsx Details about clades analyzed.

Data Set S2 mbio.01913-24-s0002.xlsx Details about antimicrobial resistance in the clades analyzed.

Supplemental Figures mbio.01913-24-s0003.pdf Figures S1 to S25.

Additional experimental details mbio.01913-24-s0004.docx Detailed experimental procedures and legends to supplemental figures.

Supplemental Tables mbio.01913-24-s0005.docx Tables S1 to S3.

ASM does not own the copyrights to Supplemental Material that may be linked to, or accessed through, an article. The authors have granted ASM a non-exclusive, world-wide license to publish the Supplemental Material files. Please contact the corresponding author directly for reuse.
