# Supplementary material for: Salmonella associated with agricultural animals exhibit diverse evolutionary rates and show evidence of recent clonal expansion
Source: mBio. 2024 Sep 17;15(10):e01913-24. doi: 10.1128/mbio.01913-24 (PMC11492988; doi:10.1128/mbio.01913-24)
Supplement: Supplemental Figures — Figures S1 to S25. [file mbio.01913-24-s0003.pdf]

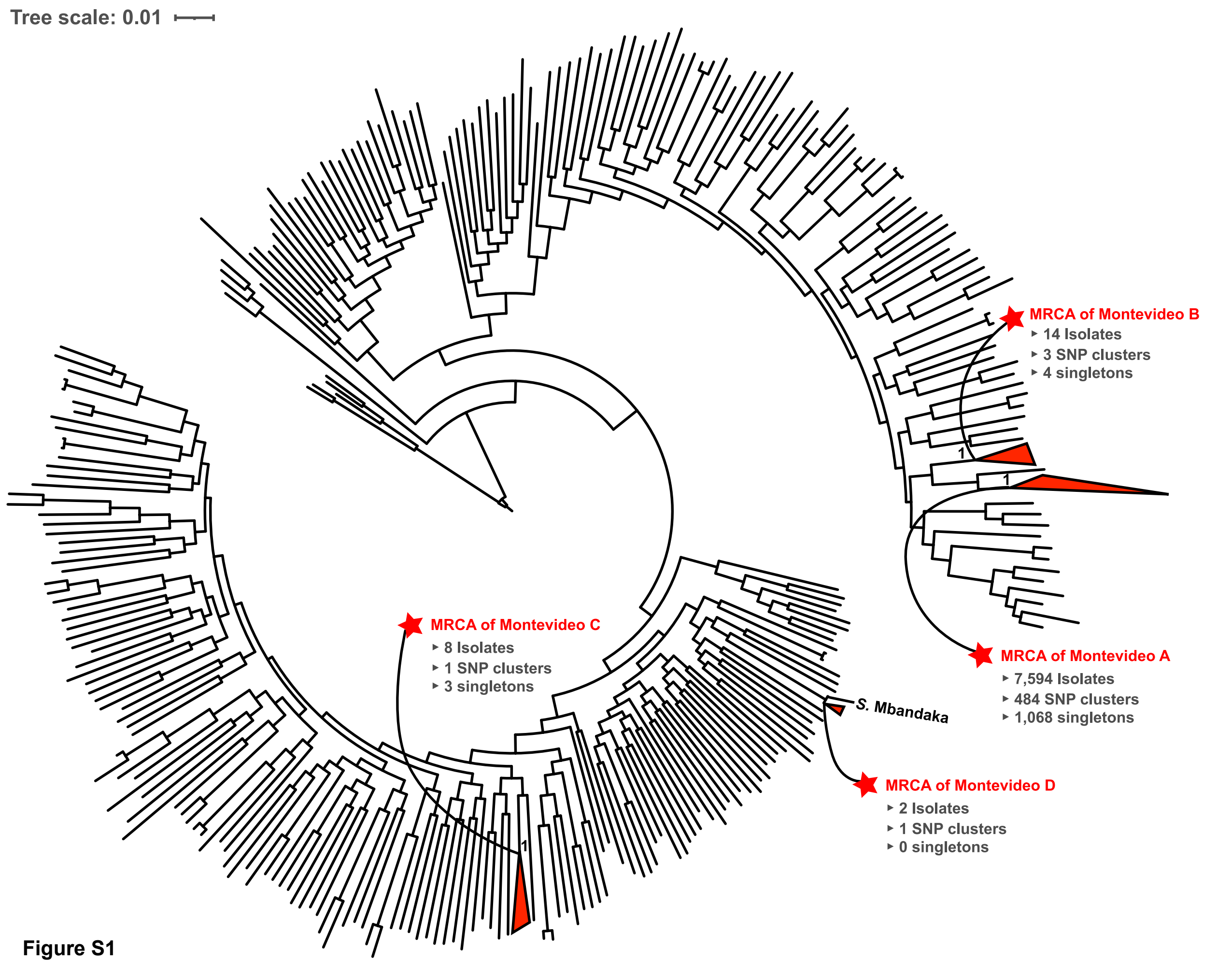
Tree scale: 0.01
MRCA of Montevideo B
14 Isolates
3 SNP clusters
4 singletons
1
1
MRCA of Montevideo A
7,594 Isolates
484 SNP clusters
1,068 singletons
MRCA of Montevideo C
8 Isolates
1 SNP clusters
3 singletons
1
S. Mbandaka
MRCA of Montevideo D
2 Isolates
1 SNP clusters
0 singletons

Figure S1

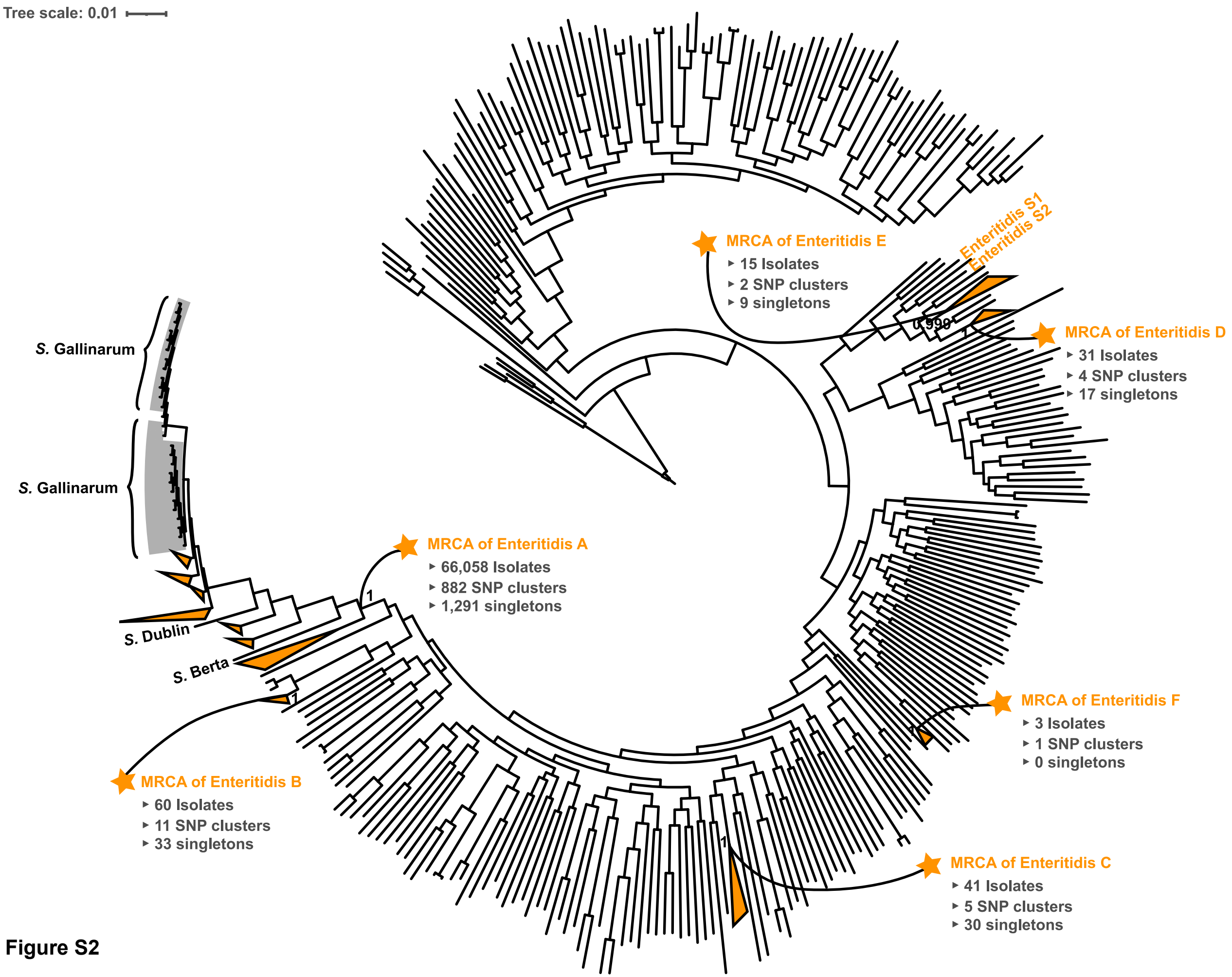

Tree scale: 0.01
S. Gallinarum
S. Gallinarum
S. Dublin
S. Berta
MRCA of Enteritidis A
66,058 Isolates
882 SNP clusters
1,291 singletons
MRCA of Enteritidis B
60 Isolates
11 SNP clusters
33 singletons
MRCA of Enteritidis C
41 Isolates
5 SNP clusters
30 singletons
MRCA of Enteritidis D
31 Isolates
4 SNP clusters
17 singletons
MRCA of Enteritidis E
15 Isolates
2 SNP clusters
9 singletons
MRCA of Enteritidis F
3 Isolates
1 SNP clusters
0 singletons
Enteritidis S1
Enteritidis S2
0.990
1
1
1
1

Figure S2

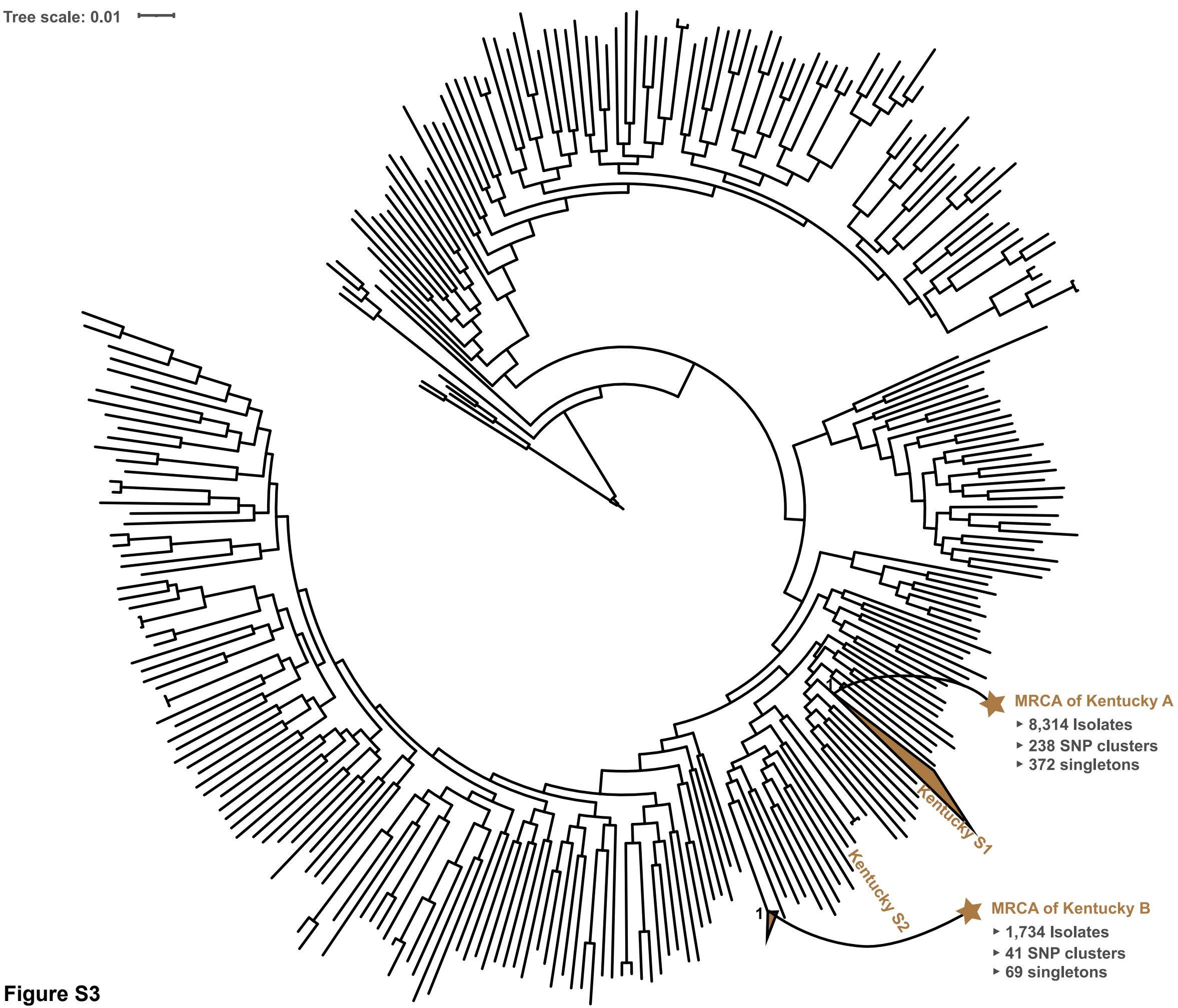
Tree scale: 0.01
MRCA of Kentucky A
8,314 Isolates
238 SNP clusters
372 singletons
Kentucky S1
1
Kentucky S2
1
MRCA of Kentucky B
1,734 Isolates
41 SNP clusters
69 singletons

Figure S3

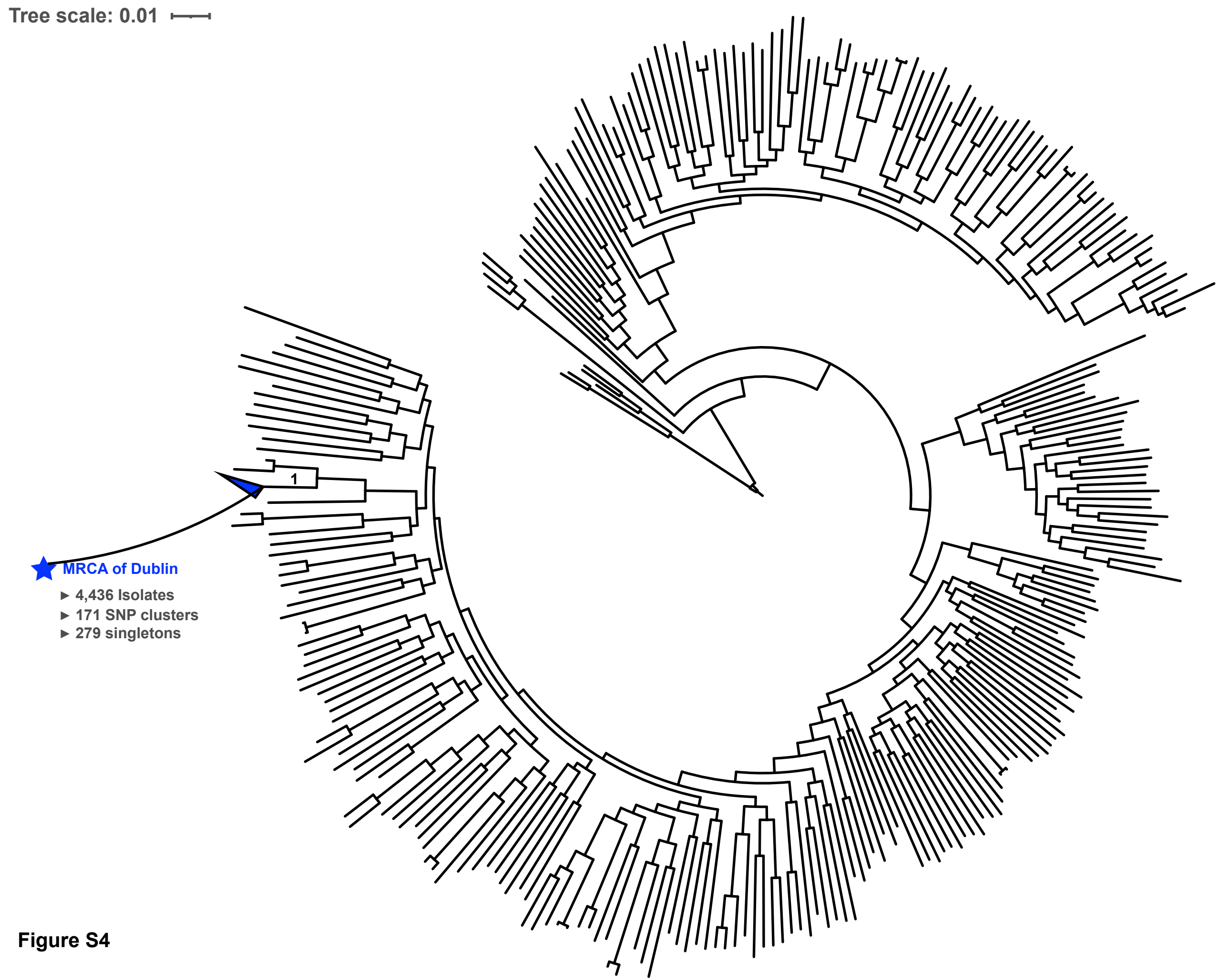
Tree scale: 0.01
1
MRCA of Dublin
► 4,436 Isolates
► 171 SNP clusters
► 279 singletons

Figure S4

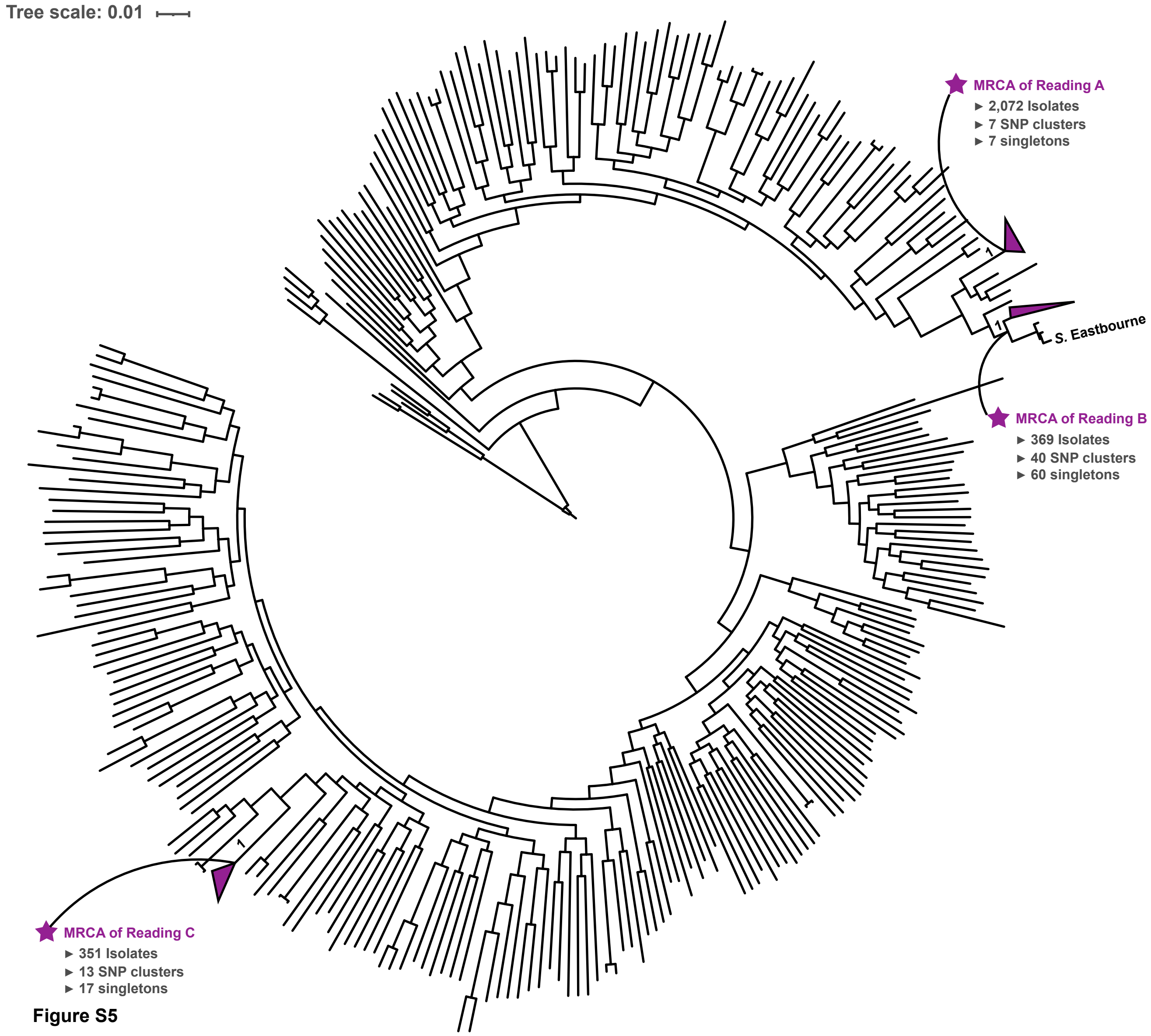
Tree scale: 0.01
MRCA of Reading A
► 2,072 Isolates
► 7 SNP clusters
► 7 singletons
1
1
S. Eastbourne
MRCA of Reading B
► 369 Isolates
► 40 SNP clusters
► 60 singletons
1
MRCA of Reading C
► 351 Isolates
► 13 SNP clusters
► 17 singletons

Figure S5

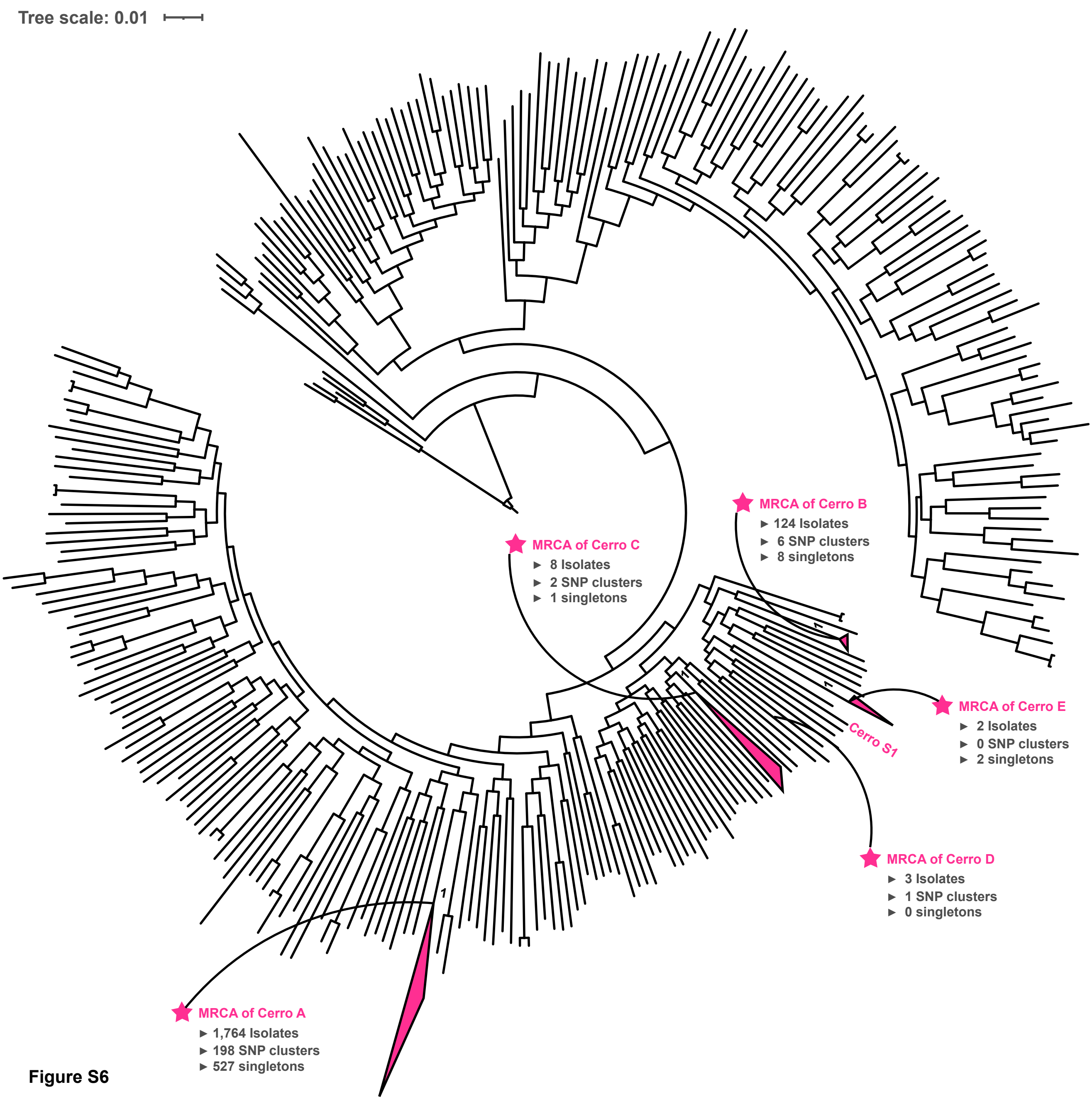

Tree scale: 0.01
MRCA of Cerro B
▶ 124 Isolates
▶ 6 SNP clusters
▶ 8 singletons
MRCA of Cerro C
▶ 8 Isolates
▶ 2 SNP clusters
▶ 1 singletons
MRCA of Cerro E
▶ 2 Isolates
▶ 0 SNP clusters
▶ 2 singletons
Cerro S1
MRCA of Cerro D
▶ 3 Isolates
▶ 1 SNP clusters
▶ 0 singletons
1
MRCA of Cerro A
▶ 1,764 Isolates
▶ 198 SNP clusters
▶ 527 singletons

Figure S6

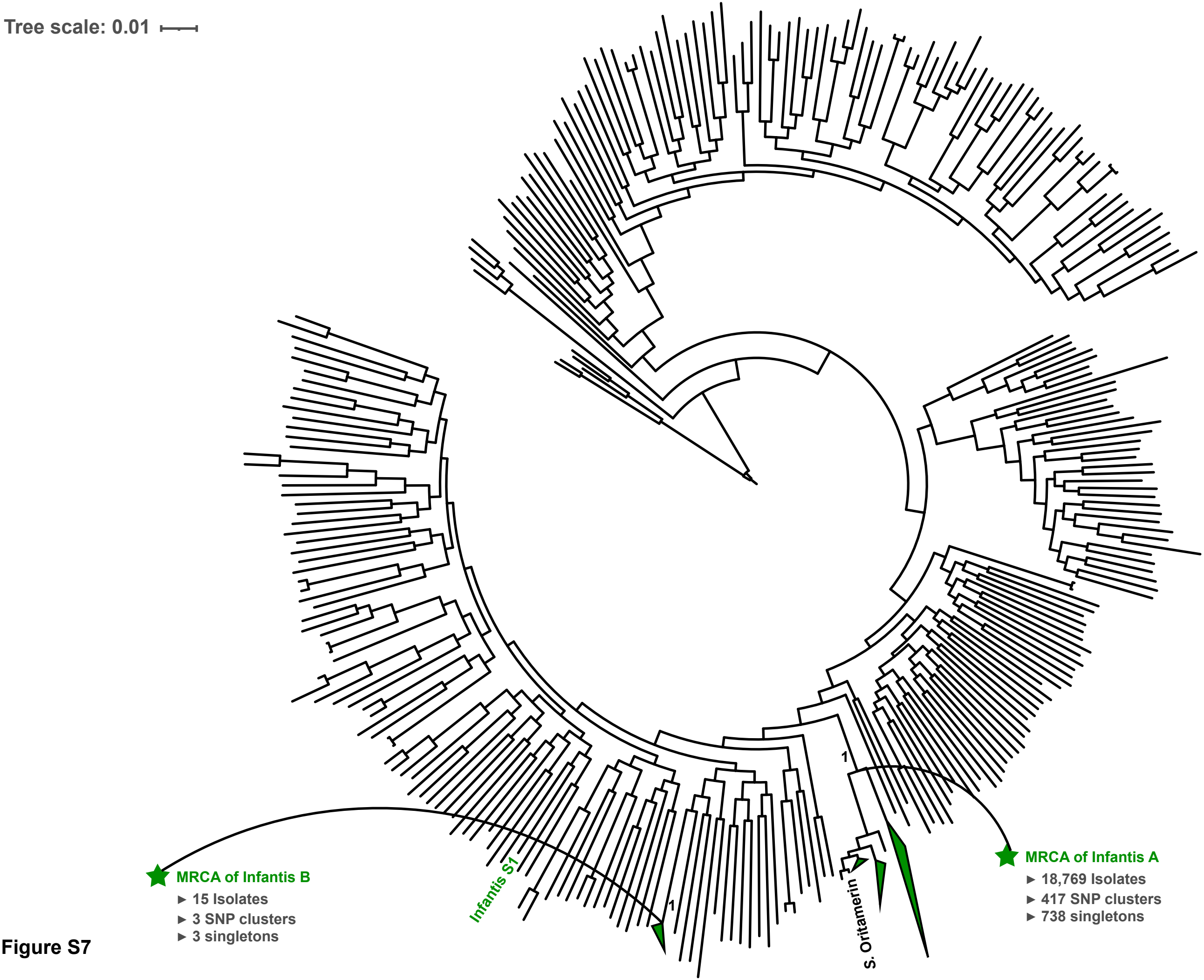
Tree scale: 0.01
Infantis S1
S. Oritamerin
1
1
MRCA of Infantis B
► 15 Isolates
► 3 SNP clusters
► 3 singletons
MRCA of Infantis A
► 18,769 Isolates
► 417 SNP clusters
► 738 singletons

Figure S7

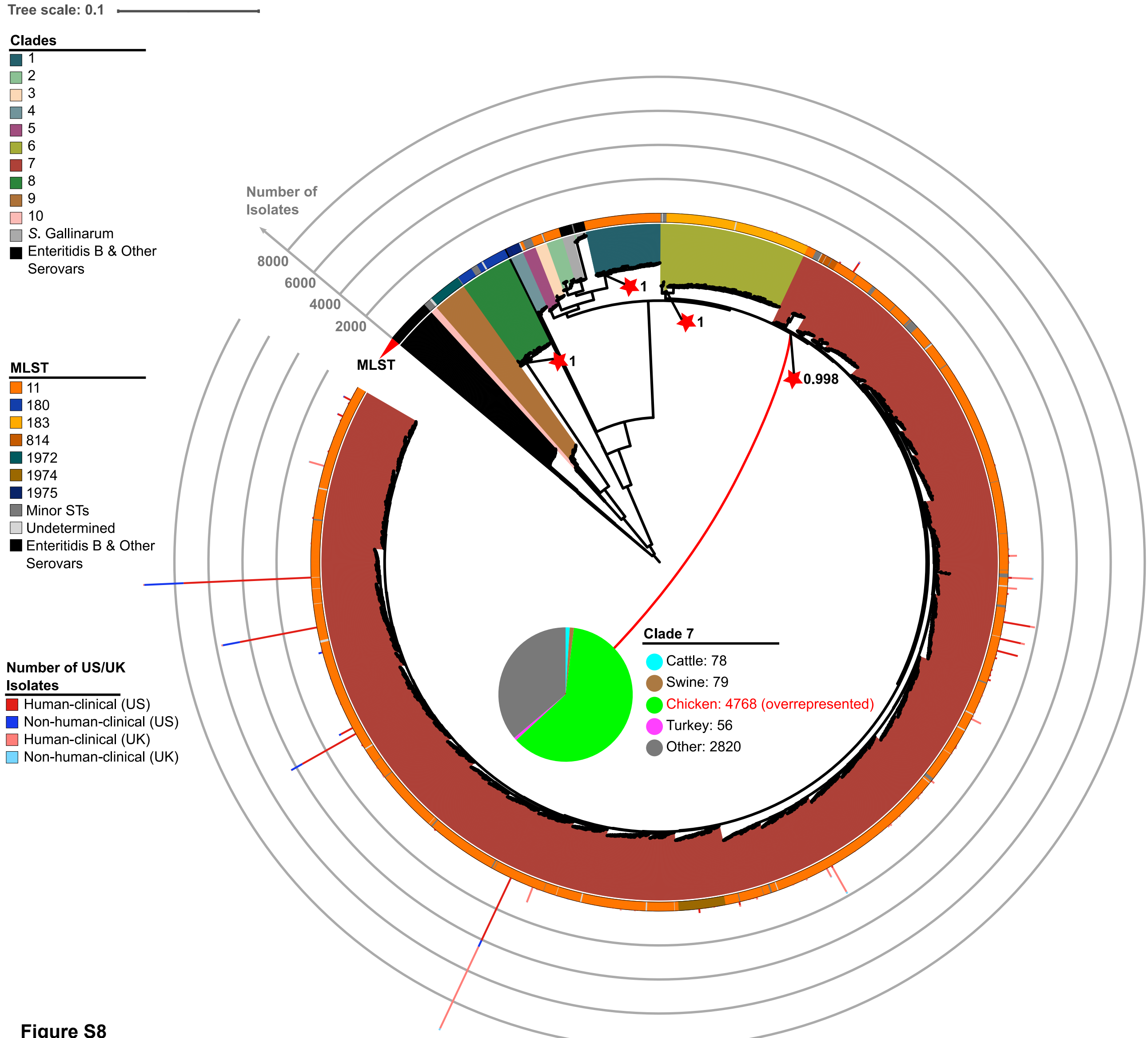

Tree scale: 0.1
Clades
1
2
3
4
5
6
7
8
9
10
S. Gallinarum
Enteritidis B & Other Serovars
MLST
11
180
183
814
1972
1974
1975
Minor STs
Undetermined
Enteritidis B & Other Serovars
Number of US/UK Isolates
Human-clinical (US)
Non-human-clinical (US)
Human-clinical (UK)
Non-human-clinical (UK)
Number of Isolates
8000
6000
4000
2000
MLST
1
1
1
0.998
Clade 7
Cattle: 78
Swine: 79
Chicken: 4768 (overrepresented)
Turkey: 56
Other: 2820

Figure S8

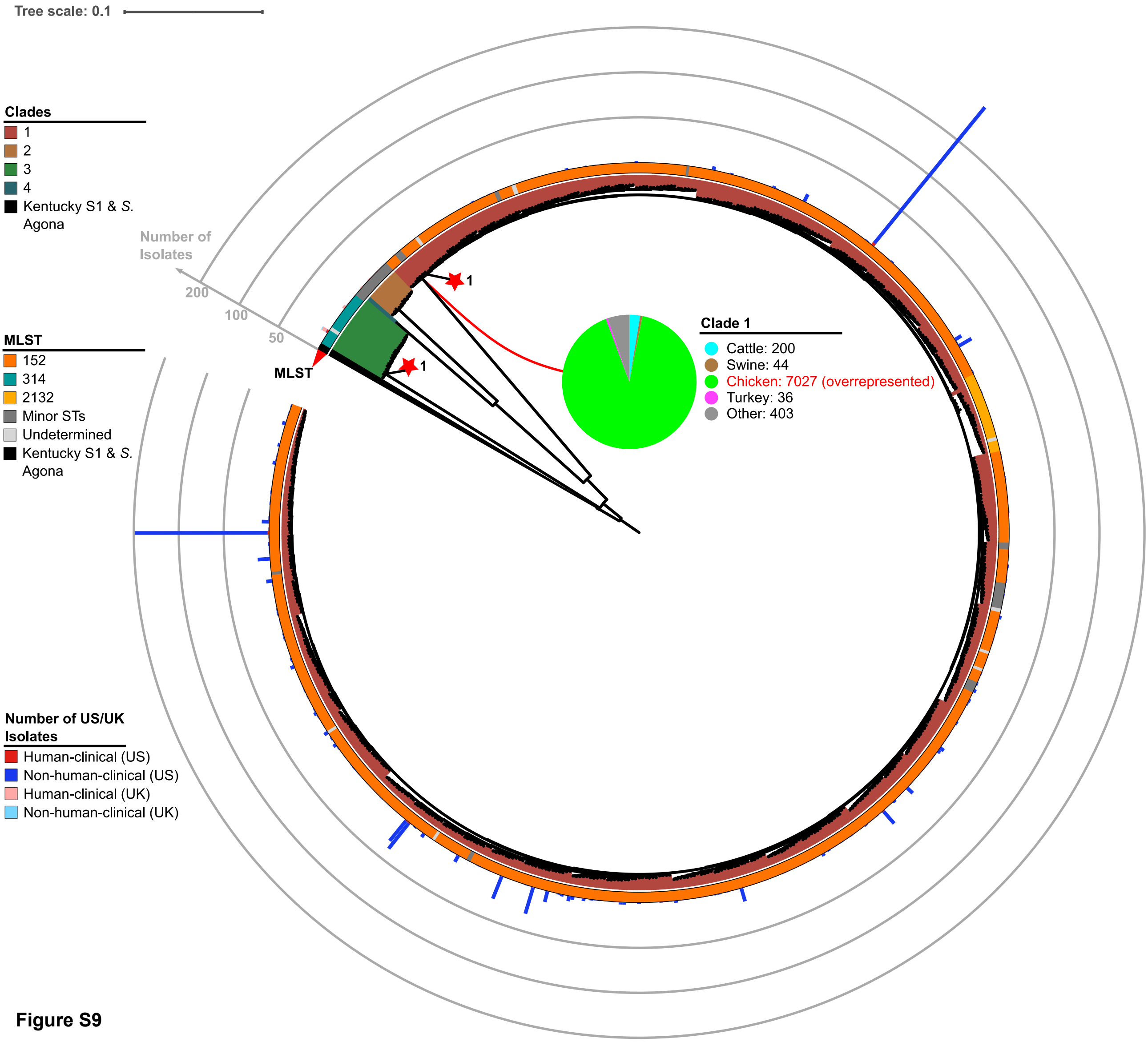

Tree scale: 0.1
Clades
1
2
3
4
Kentucky S1 & *S.* Agona
Number of Isolates
200
100
50
MLST
MLST
152
314
2132
Minor STs
Undetermined
Kentucky S1 & *S.* Agona
1
1
Clade 1
Cattle: 200
Swine: 44
Chicken: 7027 (overrepresented)
Turkey: 36
Other: 403
Number of US/UK Isolates
Human-clinical (US)
Non-human-clinical (US)
Human-clinical (UK)
Non-human-clinical (UK)

Figure S9

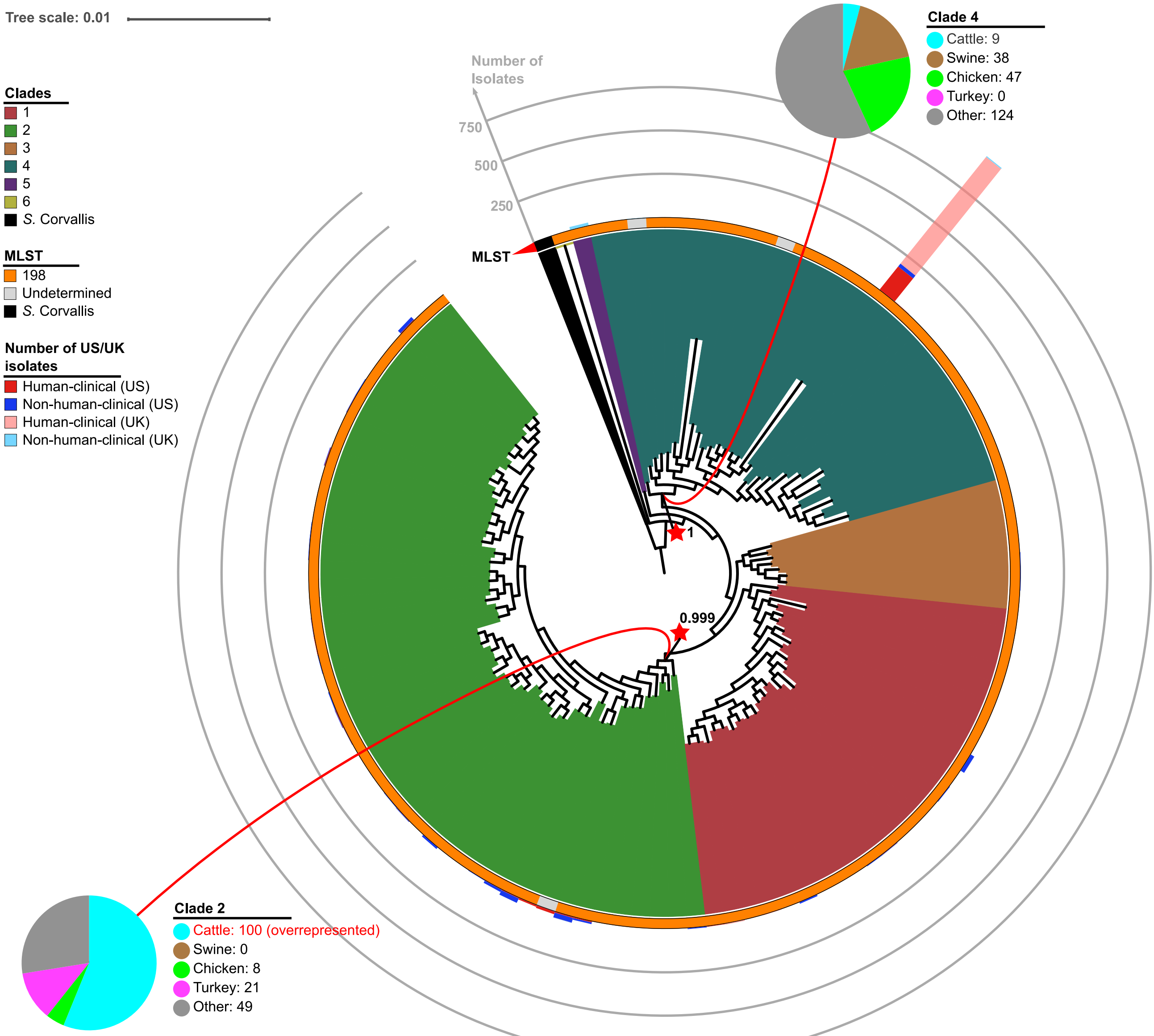

Tree scale: 0.01
Clades
1
2
3
4
5
6
S. Corvallis
MLST
198
Undetermined
S. Corvallis
Number of US/UK isolates
Human-clinical (US)
Non-human-clinical (US)
Human-clinical (UK)
Non-human-clinical (UK)
Number of Isolates
750
500
250
MLST
1
0.999
Clade 4
Cattle: 9
Swine: 38
Chicken: 47
Turkey: 0
Other: 124
Clade 2
Cattle: 100 (overrepresented)
Swine: 0
Chicken: 8
Turkey: 21
Other: 49

Figure S10

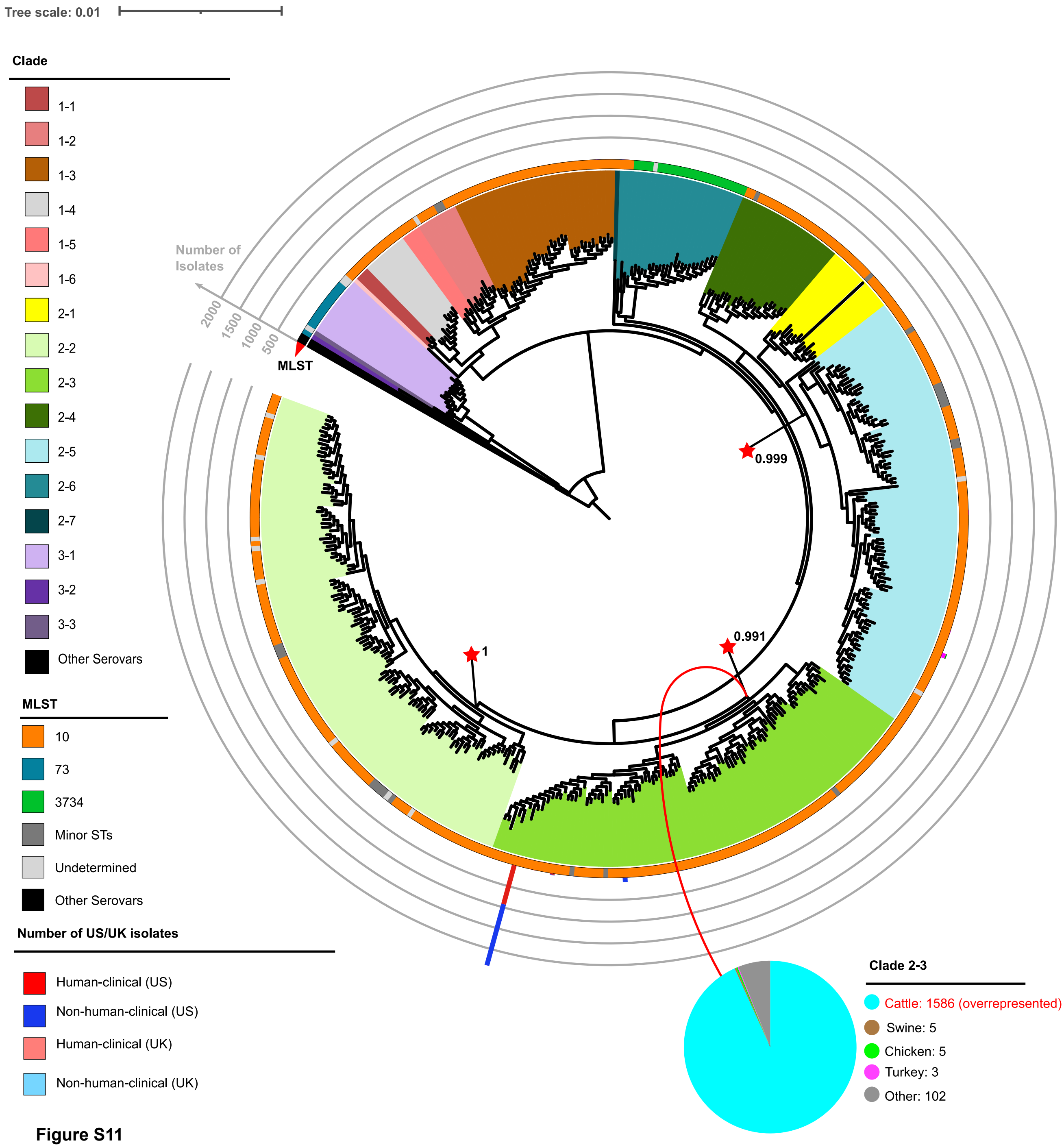

Tree scale: 0.01
Clade
1-1
1-2
1-3
1-4
1-5
1-6
2-1
2-2
2-3
2-4
2-5
2-6
2-7
3-1
3-2
3-3
Other Serovars
MLST
10
73
3734
Minor STs
Undetermined
Other Serovars
Number of US/UK isolates
Human-clinical (US)
Non-human-clinical (US)
Human-clinical (UK)
Non-human-clinical (UK)
Number of Isolates
2000
1500
1000
500
MLST
0.999
0.991
1
Clade 2-3
Cattle: 1586 (overrepresented)
Swine: 5
Chicken: 5
Turkey: 3
Other: 102

Figure S11

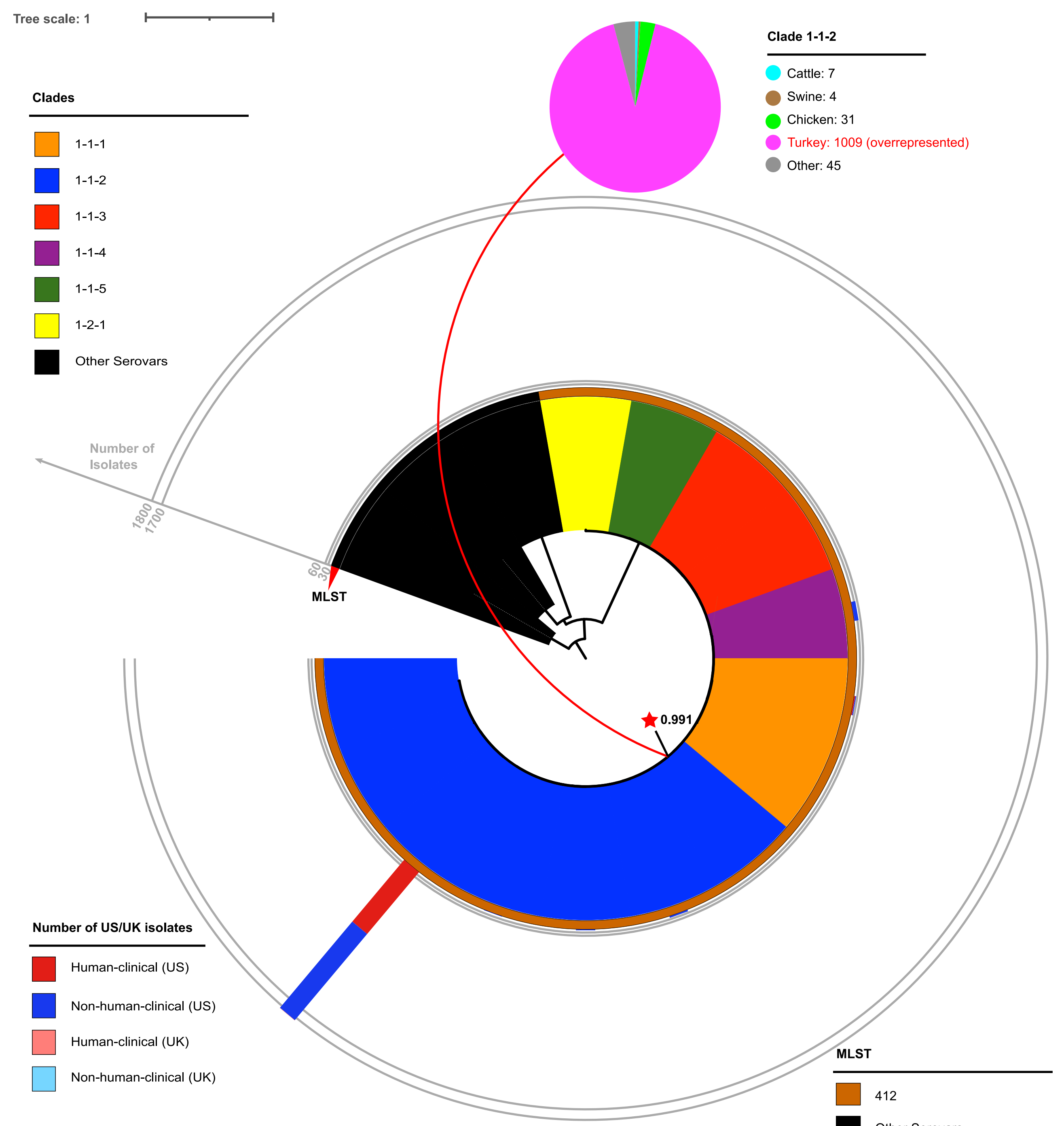
Tree scale: 1
Clade 1-1-2
Cattle: 7
Swine: 4
Chicken: 31
Turkey: 1009 (overrepresented)
Other: 45
Clades
1-1-1
1-1-2
1-1-3
1-1-4
1-1-5
1-2-1
Other Serovars
Number of Isolates
1800
1700
60
30
MLST
0.991
Number of US/UK isolates
Human-clinical (US)
Non-human-clinical (US)
Human-clinical (UK)
Non-human-clinical (UK)
MLST
412
Other Serovars

Figure S12

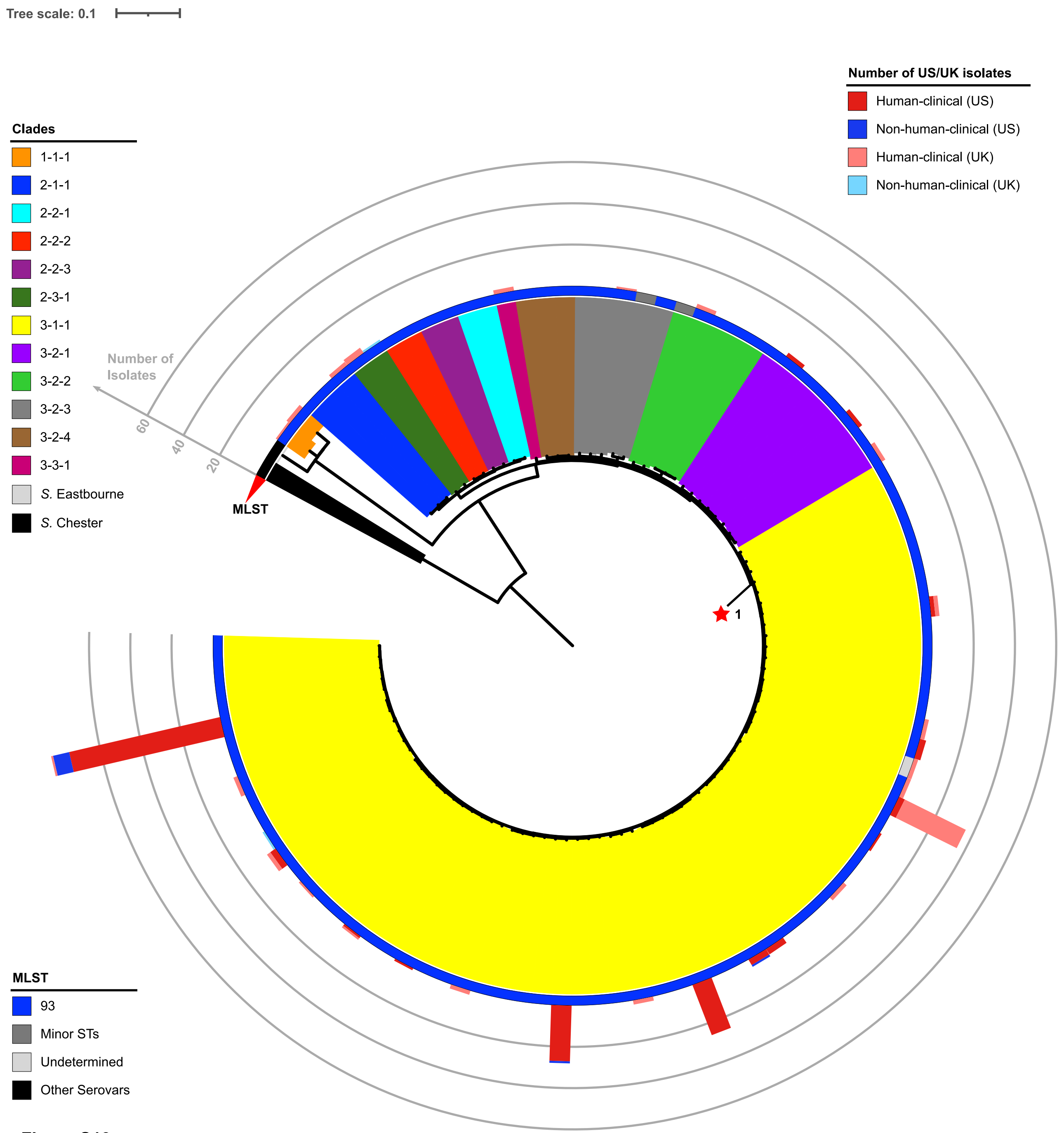
Tree scale: 0.1
Number of US/UK isolates
Human-clinical (US)
Non-human-clinical (US)
Human-clinical (UK)
Non-human-clinical (UK)
Clades
1-1-1
2-1-1
2-2-1
2-2-2
2-2-3
2-3-1
3-1-1
3-2-1
3-2-2
3-2-3
3-2-4
3-3-1
S. Eastbourne
S. Chester
Number of Isolates
60
40
20
MLST
1
MLST
93
Minor STs
Undetermined
Other Serovars

Figure S13

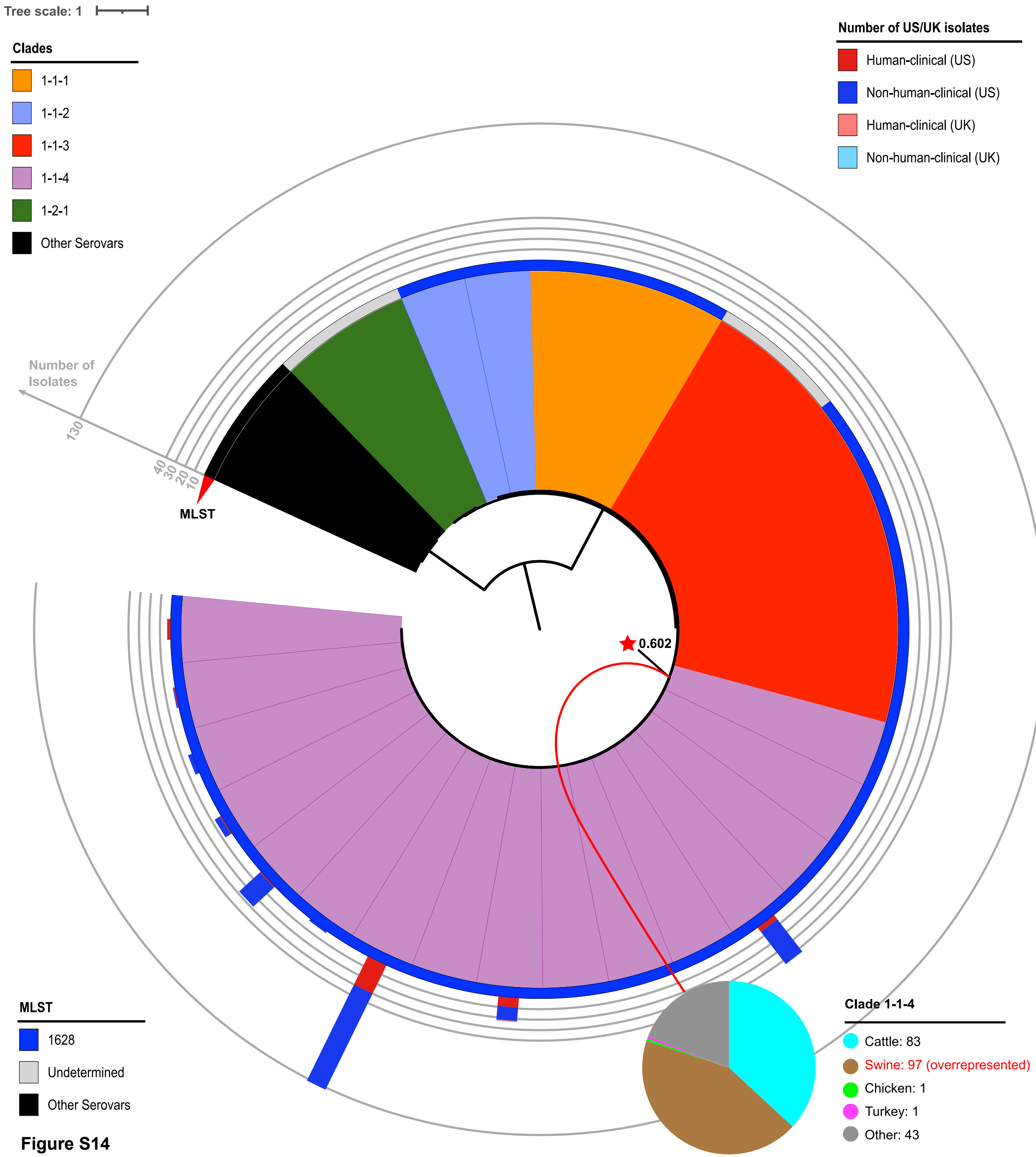

Tree scale: 1
Clades
1-1-1
1-1-2
1-1-3
1-1-4
1-2-1
Other Serovars
Number of US/UK isolates
Human-clinical (US)
Non-human-clinical (US)
Human-clinical (UK)
Non-human-clinical (UK)
Number of Isolates
130
40
30
20
10
MLST
0.602
MLST
1628
Undetermined
Other Serovars
Clade 1-1-4
Cattle: 83
Swine: 97 (overrepresented)
Chicken: 1
Turkey: 1
Other: 43

Figure S14

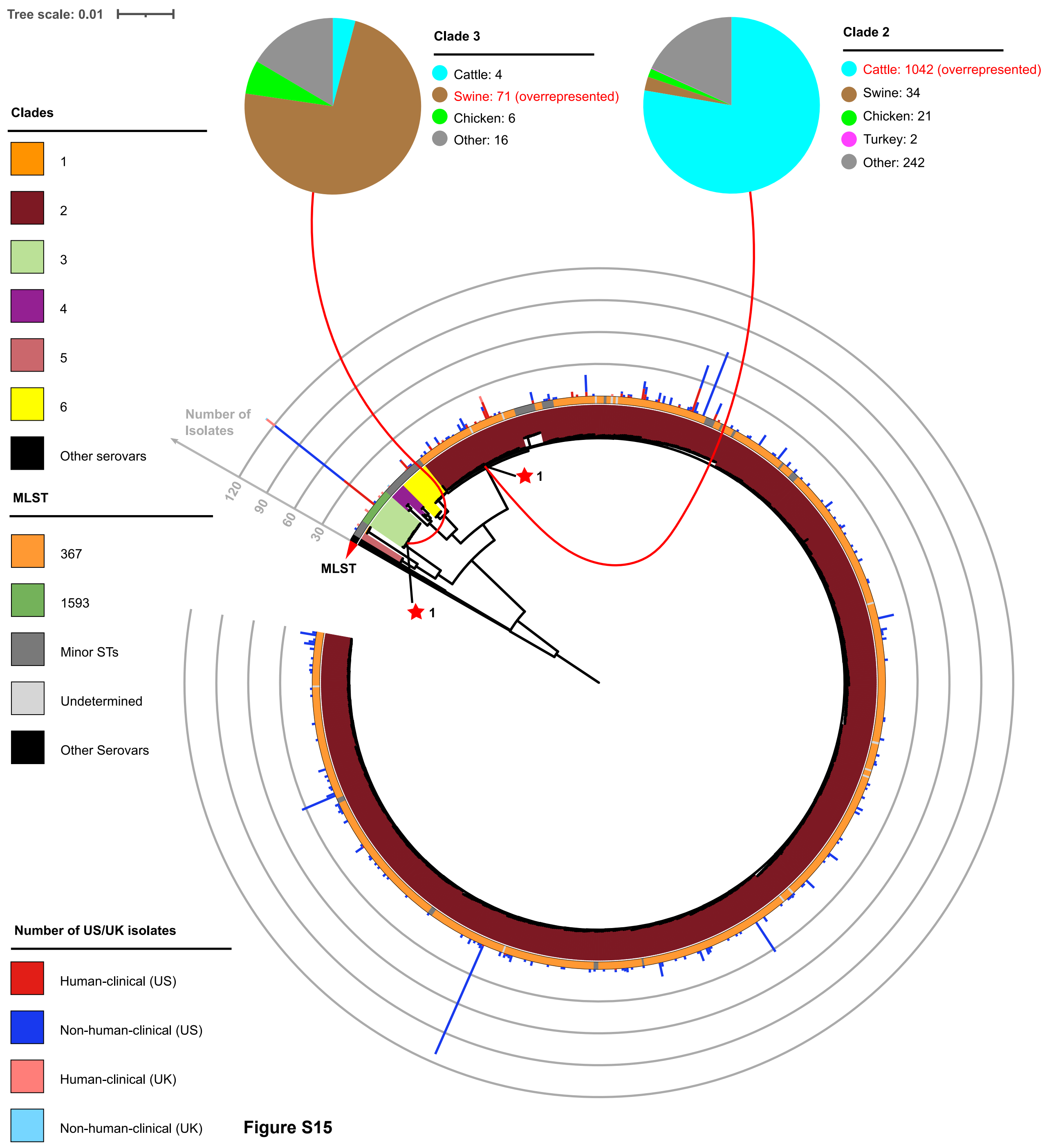

Tree scale: 0.01
Clade 3
Cattle: 4
Swine: 71 (overrepresented)
Chicken: 6
Other: 16
Clade 2
Cattle: 1042 (overrepresented)
Swine: 34
Chicken: 21
Turkey: 2
Other: 242
Clades
1
2
3
4
5
6
Other serovars
Number of Isolates
120
90
60
30
MLST
1
1
MLST
367
1593
Minor STs
Undetermined
Other Serovars
Number of US/UK isolates
Human-clinical (US)
Non-human-clinical (US)
Human-clinical (UK)
Non-human-clinical (UK)

Figure S15

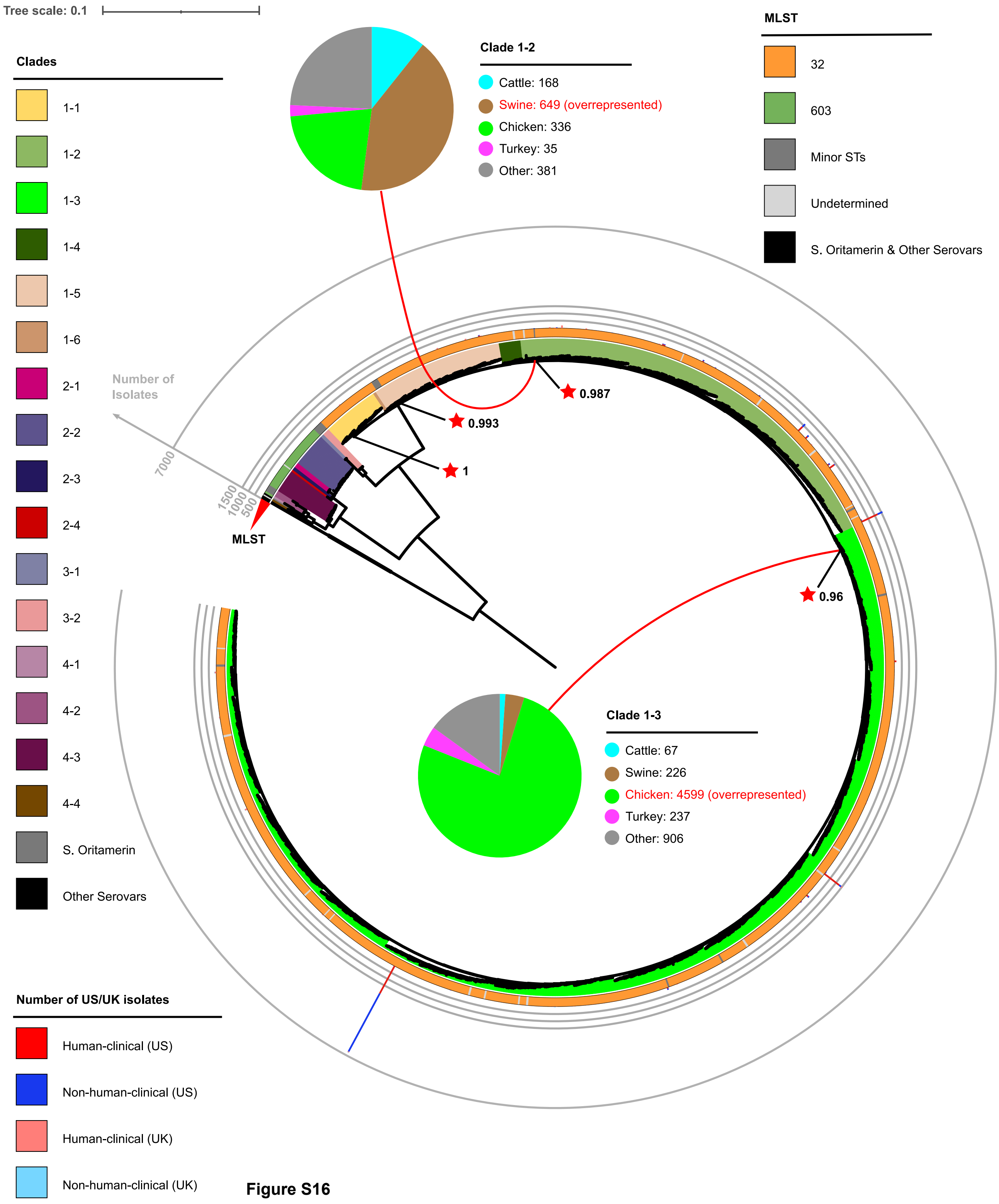
Tree scale: 0.1
Clades
1-1
1-2
1-3
1-4
1-5
1-6
2-1
2-2
2-3
2-4
3-1
3-2
4-1
4-2
4-3
4-4
S. Oritamerin
Other Serovars
Clade 1-2
Cattle: 168
Swine: 649 (overrepresented)
Chicken: 336
Turkey: 35
Other: 381
MLST
32
603
Minor STs
Undetermined
S. Oritamerin & Other Serovars
Number of Isolates
7000
1500
1000
500
MLST
0.987
0.993
1
0.96
Clade 1-3
Cattle: 67
Swine: 226
Chicken: 4599 (overrepresented)
Turkey: 237
Other: 906
Number of US/UK isolates
Human-clinical (US)
Non-human-clinical (US)
Human-clinical (UK)
Non-human-clinical (UK)

Figure S16

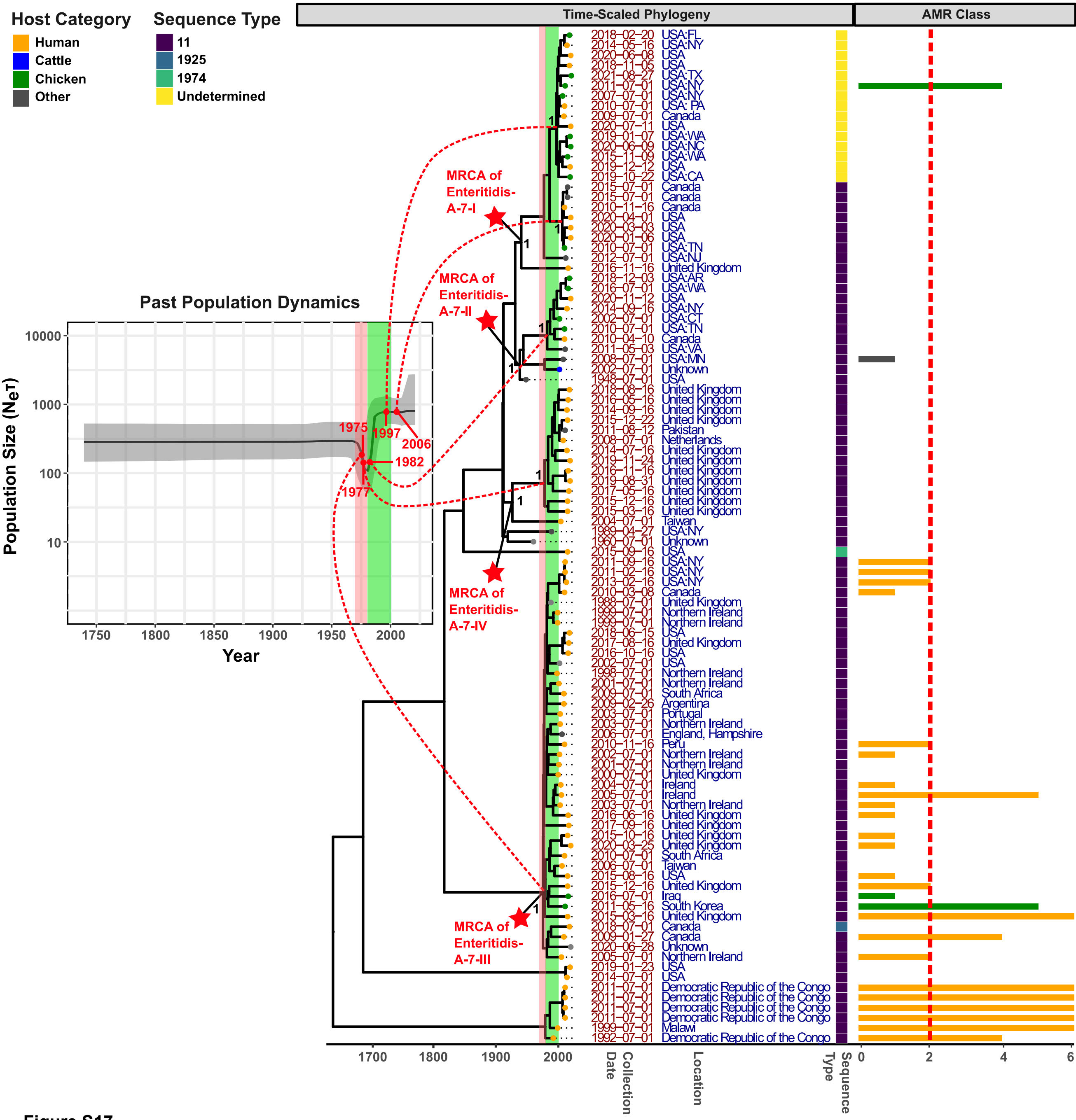
Host Category
Human
Cattle
Chicken
Other
Sequence Type
11
1925
1974
Undetermined
Time-Scaled Phylogeny
AMR Class
Past Population Dynamics
Population Size (Neτ)
Year
1975
1977
1982
1997
2006
MRCA of Enteritidis-A-7-I
MRCA of Enteritidis-A-7-II
MRCA of Enteritidis-A-7-III
MRCA of Enteritidis-A-7-IV
Collection Date
Location
Sequence Type

Figure S17

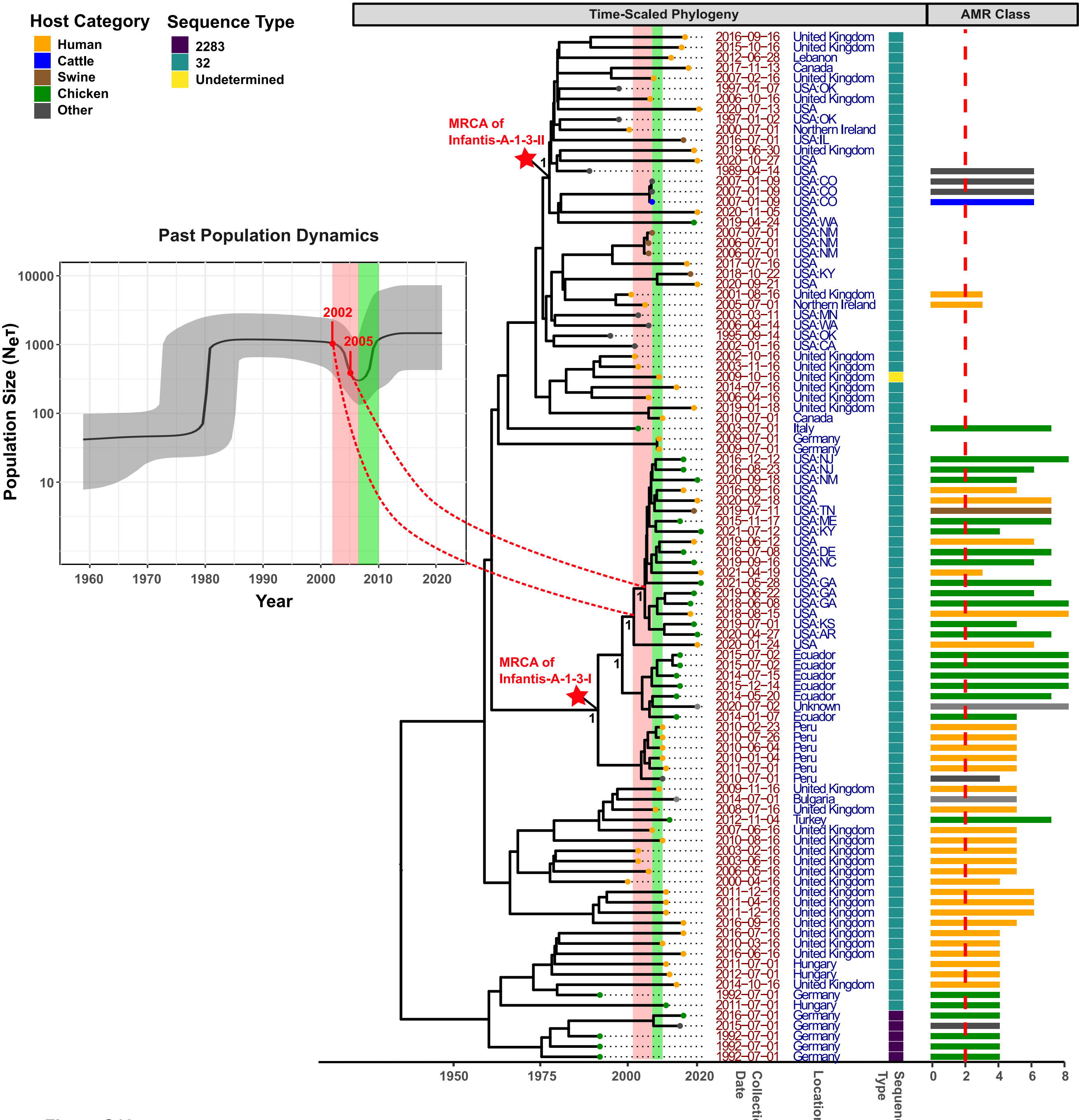

Host Category
Human
Cattle
Swine
Chicken
Other
Sequence Type
2283
32
Undetermined
Time-Scaled Phylogeny
AMR Class
Past Population Dynamics
Population Size (NeT)
10000
1000
100
10
1960
1970
1980
1990
2000
2010
2020
Year
2002
2005
MRCA of Infantis-A-1-3-II
MRCA of Infantis-A-1-3-I
1
1950
1975
2000
2020
Collection Date
Location
Sequence Type
0
2
4
6
8
2016-09-16 United Kingdom
2015-10-16 United Kingdom
2012-06-28 Lebanon
2017-11-13 Canada
2007-02-16 United Kingdom
1997-01-07 USA:OK
2006-10-16 United Kingdom
2020-07-13 USA
1997-01-02 USA:OK
2000-07-01 Northern Ireland
2016-07-01 USA:IL
2019-06-30 United Kingdom
2020-10-27 USA
1989-04-14 USA
2007-01-09 USA:CO
2007-01-09 USA:CO
2007-01-09 USA:CO
2020-11-05 USA
2019-04-24 USA:WA
2007-07-01 USA:NM
2006-07-01 USA:NM
2006-07-01 USA:NM
2017-07-16 USA
2018-10-22 USA:KY
2020-09-21 USA
2001-08-16 United Kingdom
2005-07-01 Northern Ireland
2003-03-11 USA:MN
2006-04-14 USA:WA
1995-09-14 USA:OK
2002-01-16 USA:CA
2002-10-16 United Kingdom
2003-11-16 United Kingdom
2009-10-16 United Kingdom
2014-07-16 United Kingdom
2006-04-16 United Kingdom
2019-01-18 United Kingdom
2010-07-01 Canada
2003-07-01 Italy
2009-07-01 Germany
2009-07-01 Germany
2016-12-12 USA:NJ
2016-08-23 USA:NJ
2020-09-18 USA:NM
2016-09-16 USA
2020-02-18 USA
2019-07-11 USA:TN
2015-11-17 USA:ME
2021-07-12 USA:KY
2019-06-12 USA
2016-07-08 USA:DE
2019-09-16 USA:NC
2021-04-19 USA
2021-05-28 USA:GA
2019-06-22 USA:GA
2018-06-08 USA:GA
2018-08-15 USA
2019-07-01 USA:KS
2020-04-27 USA:AR
2020-01-24 USA
2015-07-02 Ecuador
2015-07-02 Ecuador
2014-07-15 Ecuador
2015-12-14 Ecuador
2014-05-20 Ecuador
2020-07-02 Unknown
2014-01-07 Ecuador
2010-02-23 Peru
2010-07-26 Peru
2010-06-04 Peru
2010-01-04 Peru
2011-07-01 Peru
2010-07-01 Peru
2009-11-16 United Kingdom
2014-07-01 Bulgaria
2008-07-16 United Kingdom
2012-11-04 Turkey
2007-06-16 United Kingdom
2010-08-16 United Kingdom
2003-02-16 United Kingdom
2003-06-16 United Kingdom
2006-05-16 United Kingdom
2000-04-16 United Kingdom
2011-12-16 United Kingdom
2011-04-16 United Kingdom
2011-12-16 United Kingdom
2016-09-16 United Kingdom
2016-07-16 United Kingdom
2010-03-16 United Kingdom
2016-06-16 United Kingdom
2011-07-01 Hungary
2012-07-01 Hungary
2014-10-16 United Kingdom
1992-07-01 Germany
2011-07-01 Hungary
2016-07-01 Germany
2015-07-01 Germany
1992-07-01 Germany
1992-07-01 Germany
1992-07-01 Germany

FIgure S18

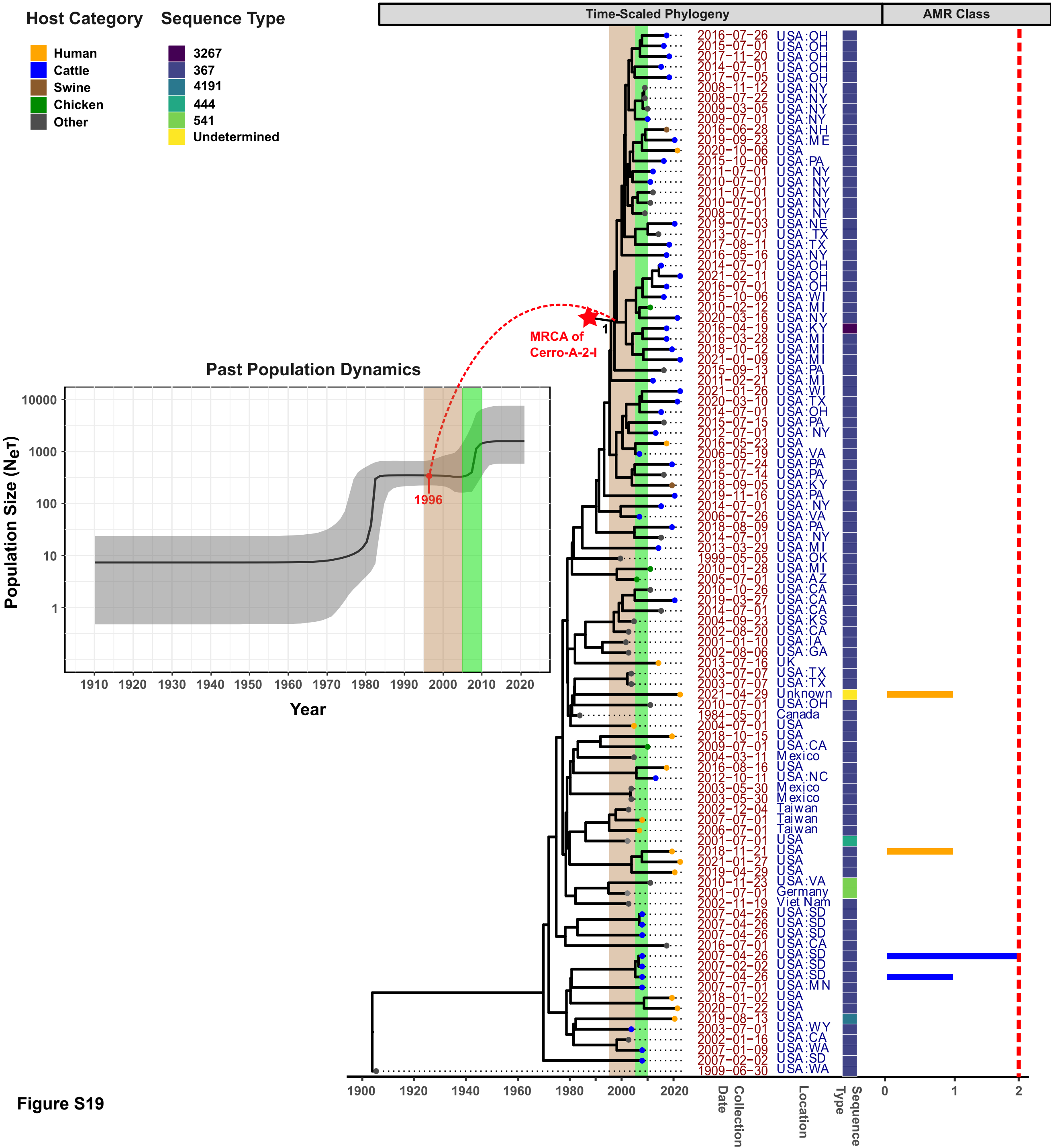

Host Category
Human
Cattle
Swine
Chicken
Other
Sequence Type
3267
367
4191
444
541
Undetermined
Time-Scaled Phylogeny
AMR Class
MRCA of Cerro-A-2-I
1
Past Population Dynamics
Population Size (NeT)
10000
1000
100
10
1
1996
1910 1920 1930 1940 1950 1960 1970 1980 1990 2000 2010 2020
Year
2016-07-26 USA:OH
2015-07-01 USA:OH
2017-11-20 USA:OH
2014-07-01 USA:OH
2017-07-05 USA:OH
2008-11-12 USA:NY
2008-07-22 USA:NY
2009-03-05 USA:NY
2009-07-01 USA:NY
2016-06-28 USA:NH
2019-09-23 USA:ME
2020-10-06 USA
2015-10-06 USA:PA
2011-07-01 USA: NY
2010-07-01 USA: NY
2011-07-01 USA: NY
2010-07-01 USA: NY
2008-07-01 USA: NY
2019-07-03 USA:NE
2013-07-01 USA: TX
2017-08-11 USA:TX
2016-05-16 USA:NY
2014-07-01 USA:OH
2021-02-11 USA:OH
2016-07-01 USA:OH
2015-10-06 USA:WI
2010-02-12 USA:MI
2020-03-16 USA:NY
2016-04-19 USA:KY
2016-03-28 USA:MI
2018-10-12 USA:MI
2021-01-09 USA:MI
2015-09-13 USA:PA
2011-02-21 USA:MI
2021-01-26 USA:WI
2020-03-10 USA:TX
2014-07-01 USA:OH
2015-07-15 USA:PA
2012-07-01 USA: NY
2016-05-23 USA
2006-05-19 USA:VA
2018-07-24 USA:PA
2015-07-14 USA:PA
2018-09-05 USA:KY
2019-11-16 USA:PA
2014-07-01 USA: NY
2006-07-26 USA:VA
2018-08-09 USA:PA
2014-07-01 USA: NY
2013-03-29 USA:MI
1999-05-05 USA:OK
2010-01-28 USA:MI
2005-07-01 USA:AZ
2010-10-26 USA:CA
2019-03-27 USA:CA
2014-07-01 USA:CA
2004-09-23 USA:KS
2002-08-20 USA:CA
2001-01-10 USA:IA
2002-08-06 USA:GA
2013-07-16 UK
2003-07-07 USA:TX
2003-07-07 USA:TX
2021-04-29 Unknown
2010-07-01 USA:OH
1984-05-01 Canada
2004-07-01 USA
2018-10-15 USA
2009-07-01 USA:CA
2004-03-11 Mexico
2016-08-16 USA
2012-10-11 USA:NC
2003-05-30 Mexico
2003-05-30 Mexico
2002-12-04 Taiwan
2007-07-01 Taiwan
2006-07-01 Taiwan
2001-07-01 USA
2018-11-21 USA
2021-01-27 USA
2019-04-29 USA
2010-11-23 USA:VA
2001-07-01 Germany
2002-11-19 Viet Nam
2007-04-26 USA:SD
2007-04-26 USA:SD
2007-04-26 USA:SD
2016-07-01 USA:CA
2007-04-26 USA:SD
2007-02-02 USA:SD
2007-04-26 USA:SD
2007-07-01 USA:MN
2018-01-02 USA
2020-07-22 USA
2019-08-13 USA
2003-07-01 USA:WY
2002-01-16 USA:CA
2007-01-09 USA:WA
2007-02-02 USA:SD
1909-06-30 USA:WA
1900 1920 1940 1960 1980 2000 2020
Collection Date
Location
Sequence Type
0 1 2

Figure S19

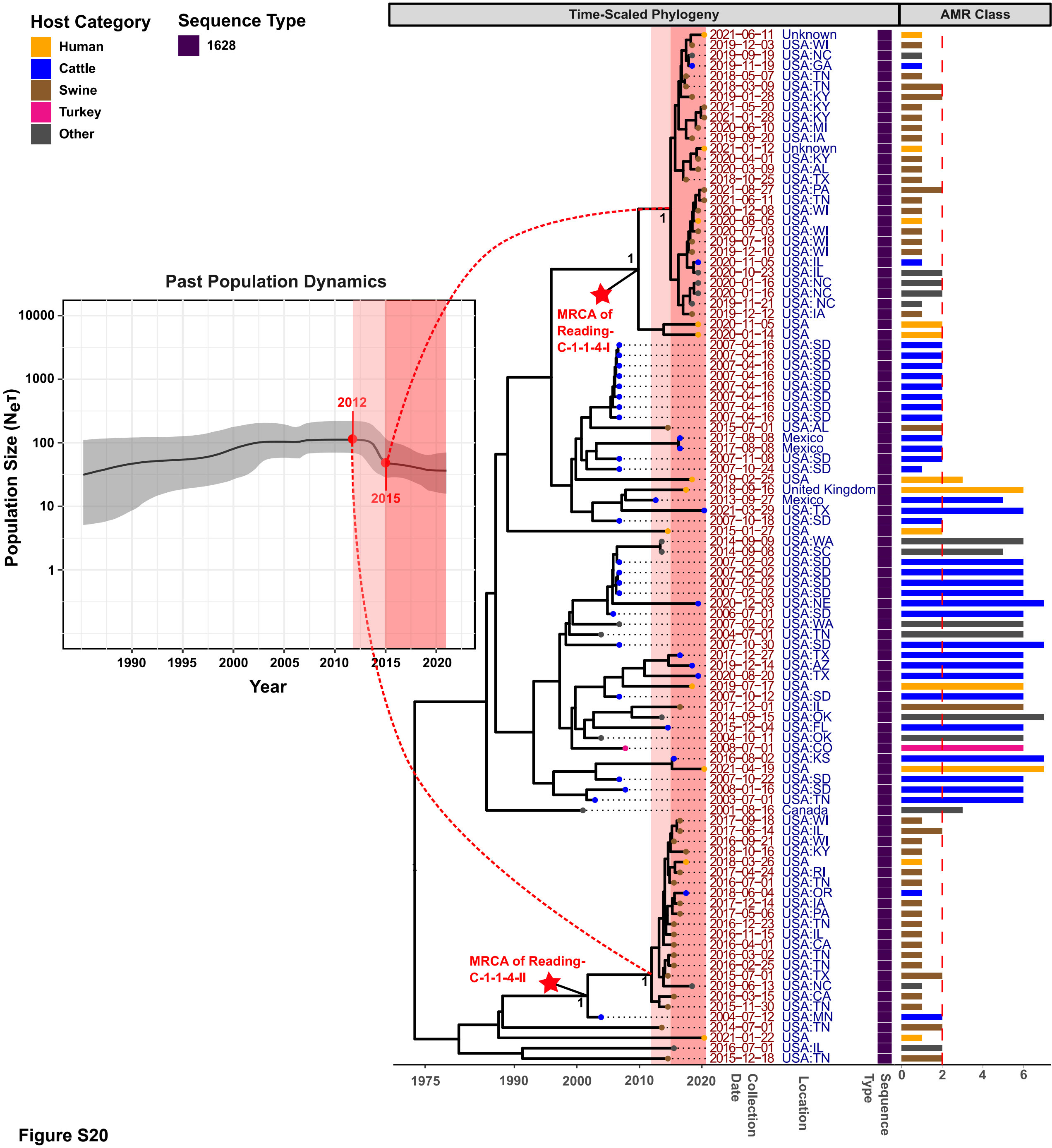

Host Category
Human
Cattle
Swine
Turkey
Other
Sequence Type
1628
Time-Scaled Phylogeny
AMR Class
Past Population Dynamics
Population Size (Neτ)
Year
2012
2015
MRCA of Reading-C-1-1-4-I
MRCA of Reading-C-1-1-4-II
Collection Date
Location
Sequence Type

Figure S20

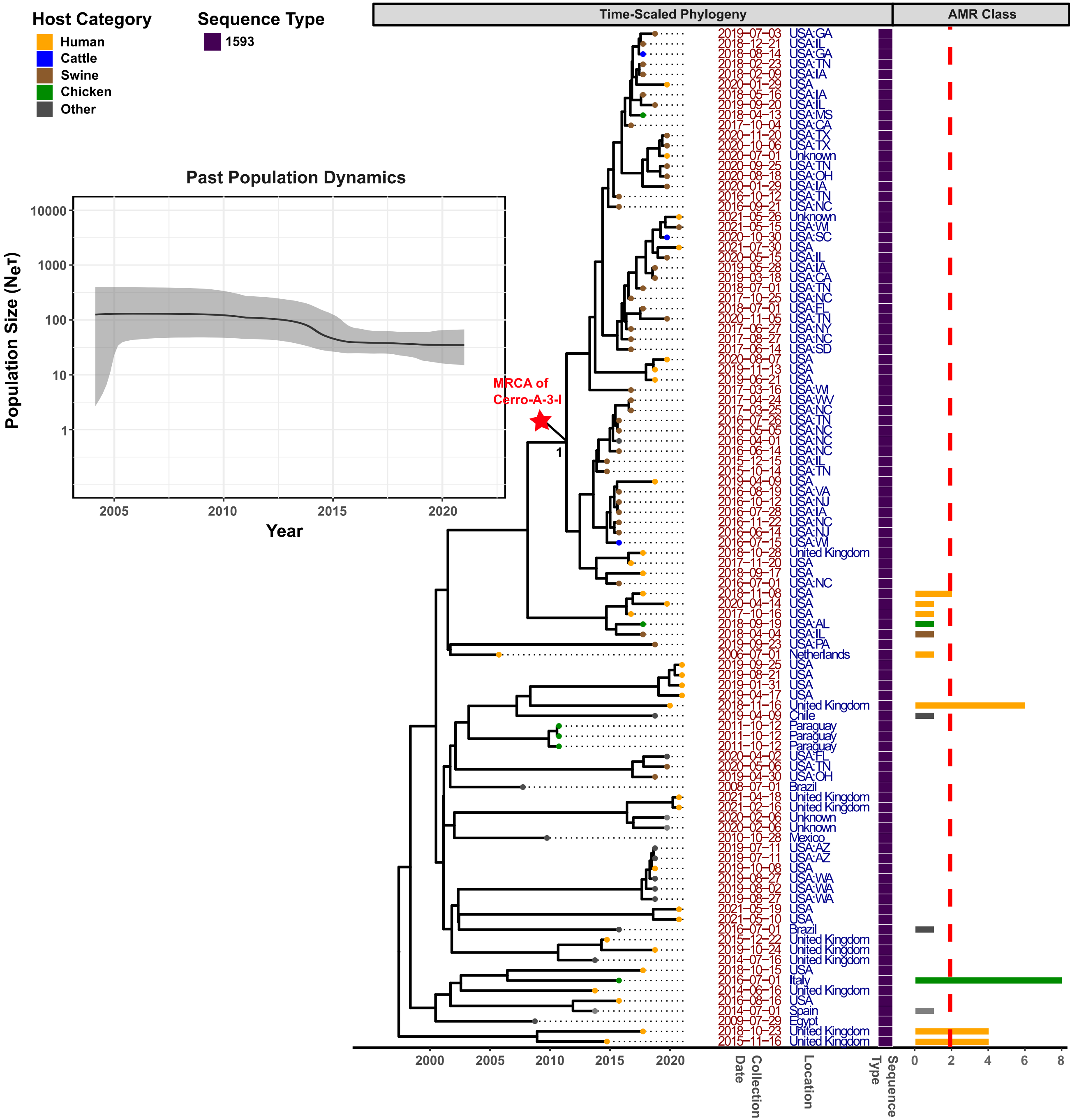

Host Category
Human
Cattle
Swine
Chicken
Other
Sequence Type
1593
Past Population Dynamics
Population Size (Neτ)
10000
1000
100
10
1
2005
2010
2015
2020
Year
Time-Scaled Phylogeny
AMR Class
MRCA of Cerro-A-3-I
1
2019-07-03 USA:GA
2018-12-21 USA:IL
2018-08-14 USA:GA
2018-02-23 USA:TN
2018-02-09 USA:IA
2020-01-29 USA
2018-05-16 USA:IA
2019-09-20 USA:IL
2018-04-13 USA:MS
2017-10-04 USA:CA
2020-11-20 USA:TX
2020-10-06 USA:TX
2020-07-01 Unknown
2020-09-25 USA:TN
2020-08-18 USA:OH
2020-01-29 USA:IA
2016-10-12 USA:TN
2016-09-21 USA:NC
2021-05-26 Unknown
2021-05-15 USA:WI
2020-10-30 USA:SC
2021-07-30 USA
2020-05-15 USA:IL
2019-05-28 USA:IA
2019-03-18 USA:CA
2018-07-01 USA:TN
2017-10-25 USA:NC
2018-07-01 USA:FL
2020-11-05 USA:TN
2017-06-27 USA:NY
2017-08-27 USA:NC
2017-06-14 USA:SD
2020-08-07 USA
2019-11-13 USA
2019-06-21 USA
2017-03-16 USA:WI
2017-04-24 USA:WV
2017-03-25 USA:NC
2016-07-26 USA:TN
2016-05-05 USA:NC
2016-04-01 USA:NC
2016-06-14 USA:NC
2015-12-15 USA:IL
2015-10-14 USA:TN
2019-04-09 USA
2016-08-19 USA:VA
2016-10-12 USA:NJ
2016-07-28 USA:IA
2016-11-22 USA:NC
2016-06-14 USA:NJ
2016-07-15 USA:WI
2018-10-28 United Kingdom
2017-11-20 USA
2018-09-17 USA
2016-07-01 USA:NC
2018-11-08 USA
2020-04-14 USA
2017-10-16 USA
2018-09-19 USA:AL
2018-04-04 USA:IL
2019-09-23 USA:PA
2006-07-01 Netherlands
2019-09-25 USA
2019-08-21 USA
2019-01-31 USA
2019-04-17 USA
2018-11-16 United Kingdom
2019-04-09 Chile
2011-10-12 Paraguay
2011-10-12 Paraguay
2011-10-12 Paraguay
2020-04-02 USA:FL
2020-05-06 USA:TN
2019-04-30 USA:OH
2008-07-01 Brazil
2021-04-18 United Kingdom
2021-02-16 United Kingdom
2020-02-06 Unknown
2020-02-06 Unknown
2010-10-28 Mexico
2019-07-11 USA:AZ
2019-07-11 USA:AZ
2019-10-08 USA
2019-08-27 USA:WA
2019-08-02 USA:WA
2019-08-27 USA:WA
2021-05-19 USA
2021-05-10 USA
2016-07-01 Brazil
2015-12-22 United Kingdom
2019-10-24 United Kingdom
2014-07-16 United Kingdom
2018-10-15 USA
2016-07-01 Italy
2014-06-16 United Kingdom
2016-08-16 USA
2014-07-01 Spain
2009-07-29 Egypt
2018-10-23 United Kingdom
2015-11-16 United Kingdom
2000
2005
2010
2015
2020
Collection Date
Location
Sequence Type
0
2
4
6
8

Figure S21

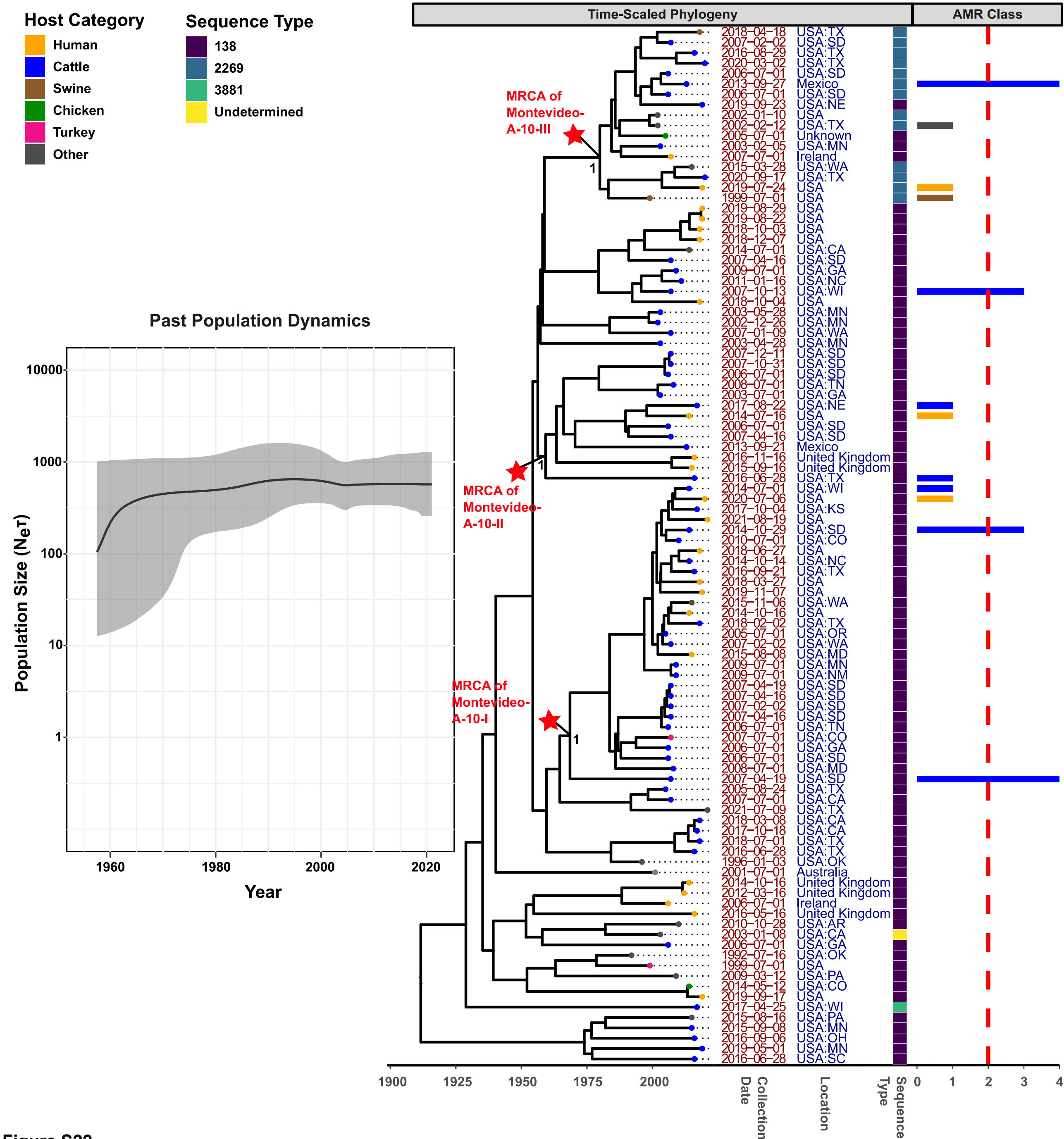

Host Category
Human
Cattle
Swine
Chicken
Turkey
Other
Sequence Type
138
2269
3881
Undetermined
Past Population Dynamics
Population Size (Neτ)
10000
1000
100
10
1
1960
1980
2000
2020
Year
Time-Scaled Phylogeny
AMR Class
MRCA of Montevideo-A-10-III
MRCA of Montevideo-A-10-II
MRCA of Montevideo-A-10-I
1
2018-04-18 USA:TX
2007-02-02 USA:SD
2016-08-29 USA:TX
2020-03-02 USA:TX
2006-07-01 USA:SD
2013-09-27 Mexico
2006-07-01 USA:SD
2019-09-23 USA:NE
2002-01-10 USA
2002-02-12 USA:TX
2005-07-01 Unknown
2003-02-05 USA:MN
2007-07-01 Ireland
2015-03-28 USA:WA
2020-09-17 USA:TX
2019-07-24 USA
1999-07-01 USA
2019-08-29 USA
2019-08-22 USA
2018-10-03 USA
2018-12-07 USA
2014-07-01 USA:CA
2007-04-16 USA:SD
2009-07-01 USA:GA
2011-01-16 USA:NC
2007-10-13 USA:WI
2018-10-04 USA
2003-05-28 USA:MN
2002-12-26 USA:MN
2007-01-09 USA:WA
2003-04-28 USA:MN
2007-12-11 USA:SD
2007-10-31 USA:SD
2006-07-01 USA:SD
2008-07-01 USA:TN
2003-07-01 USA:GA
2017-08-22 USA:NE
2014-07-16 USA
2006-07-01 USA:SD
2007-04-16 USA:SD
2013-09-21 Mexico
2016-11-16 United Kingdom
2015-09-16 United Kingdom
2016-06-28 USA:TX
2014-07-01 USA:WI
2020-07-06 USA
2017-10-04 USA:KS
2021-08-19 USA
2014-10-29 USA:SD
2010-07-01 USA:CO
2018-06-27 USA
2014-10-14 USA:NC
2016-09-21 USA:TX
2018-03-27 USA
2019-11-07 USA
2015-11-06 USA:WA
2014-10-16 USA
2018-02-02 USA:TX
2005-07-01 USA:OR
2007-02-02 USA:WA
2015-08-08 USA:MD
2009-07-01 USA:MN
2009-07-01 USA:NM
2007-04-19 USA:SD
2007-04-16 USA:SD
2007-02-02 USA:SD
2007-04-16 USA:SD
2006-07-01 USA:TN
2007-07-01 USA:CO
2006-07-01 USA:GA
2006-07-01 USA:SD
2008-07-01 USA:MD
2007-04-19 USA:SD
2005-08-24 USA:TX
2007-07-01 USA:CA
2021-07-09 USA:TX
2018-03-08 USA:CA
2017-10-18 USA:CA
2018-07-01 USA:TX
2016-06-28 USA:TX
1996-01-03 USA:OK
2001-07-01 Australia
2014-10-16 United Kingdom
2012-03-16 United Kingdom
2006-07-01 Ireland
2016-05-16 United Kingdom
2010-10-28 USA:AR
2003-01-08 USA:CA
2006-07-01 USA:GA
1992-07-16 USA:OK
1999-07-01 USA
2009-03-12 USA:PA
2014-05-12 USA:CO
2019-09-17 USA
2017-04-25 USA:WI
2015-08-16 USA:PA
2015-09-08 USA:MN
2016-09-06 USA:OH
2019-05-01 USA:MN
2016-06-28 USA:SC
1900
1925
1950
1975
2000
Collection Date
Location
Sequence Type
0
1
2
3
4

Figure S22

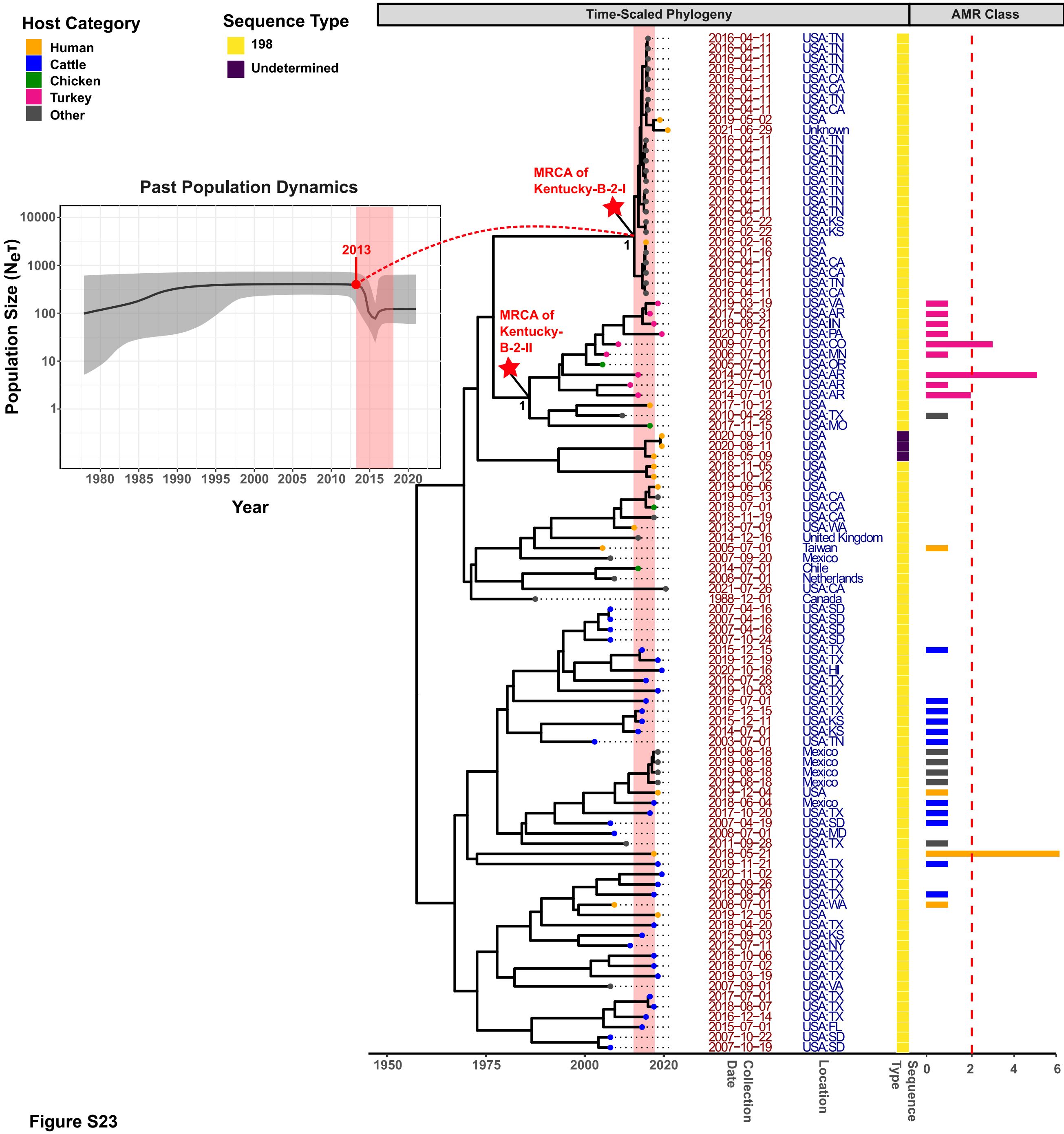
Host Category
Human
Cattle
Chicken
Turkey
Other
Sequence Type
198
Undetermined
Time-Scaled Phylogeny
AMR Class
Past Population Dynamics
Population Size (NeT)
Year
2013
MRCA of Kentucky-B-2-I
MRCA of Kentucky-B-2-II
Collection Date
Location
Sequence Type

Figure S23

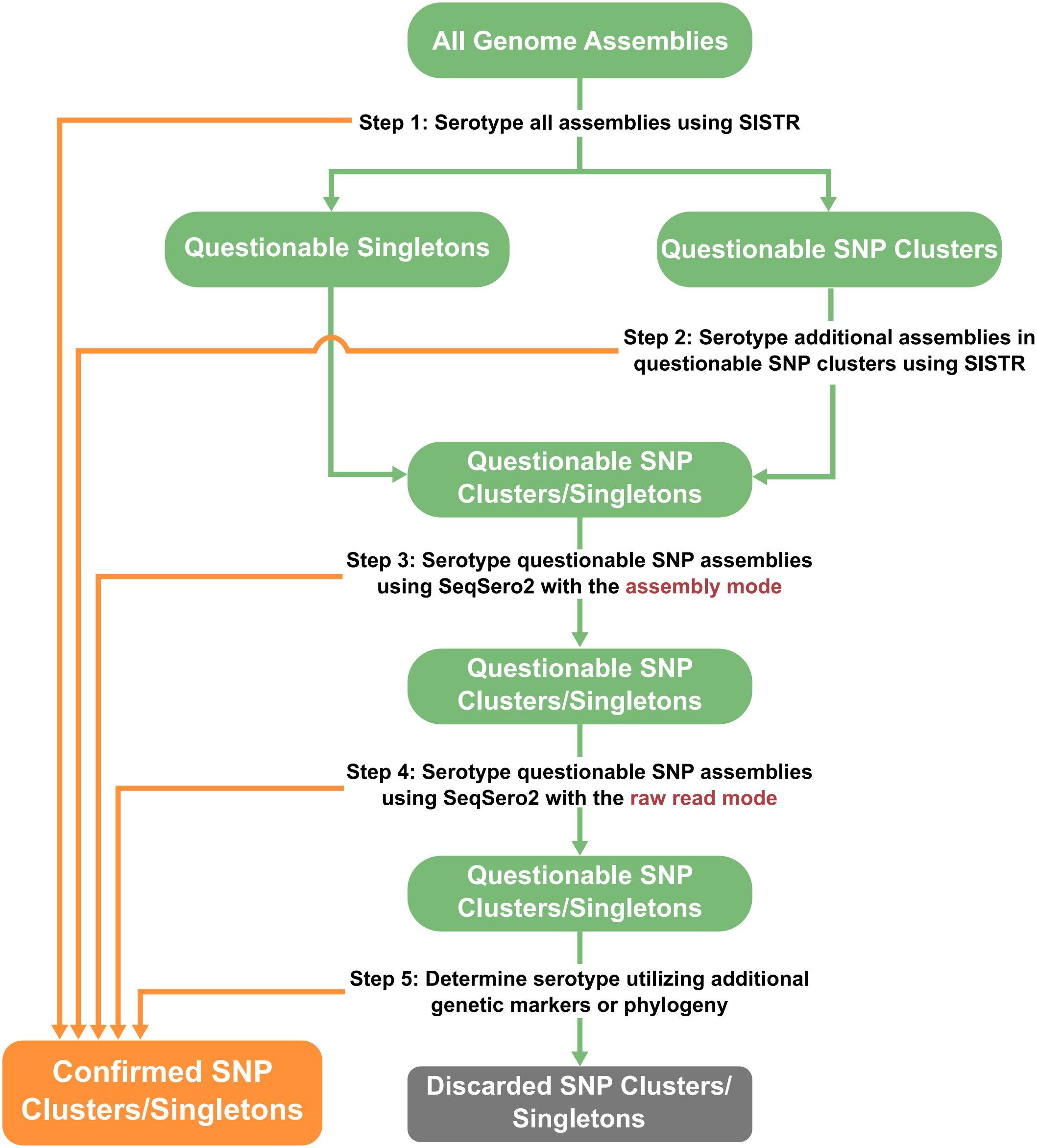
All Genome Assemblies
Step 1: Serotype all assemblies using SISTR
Questionable Singletons
Questionable SNP Clusters
Step 2: Serotype additional assemblies in questionable SNP clusters using SISTR
Questionable SNP Clusters/Singletons
Step 3: Serotype questionable SNP assemblies using SeqSero2 with the assembly mode
Questionable SNP Clusters/Singletons
Step 4: Serotype questionable SNP assemblies using SeqSero2 with the raw read mode
Questionable SNP Clusters/Singletons
Step 5: Determine serotype utilizing additional genetic markers or phylogeny
Confirmed SNP Clusters/Singletons
Discarded SNP Clusters/ Singletons

Figure S24

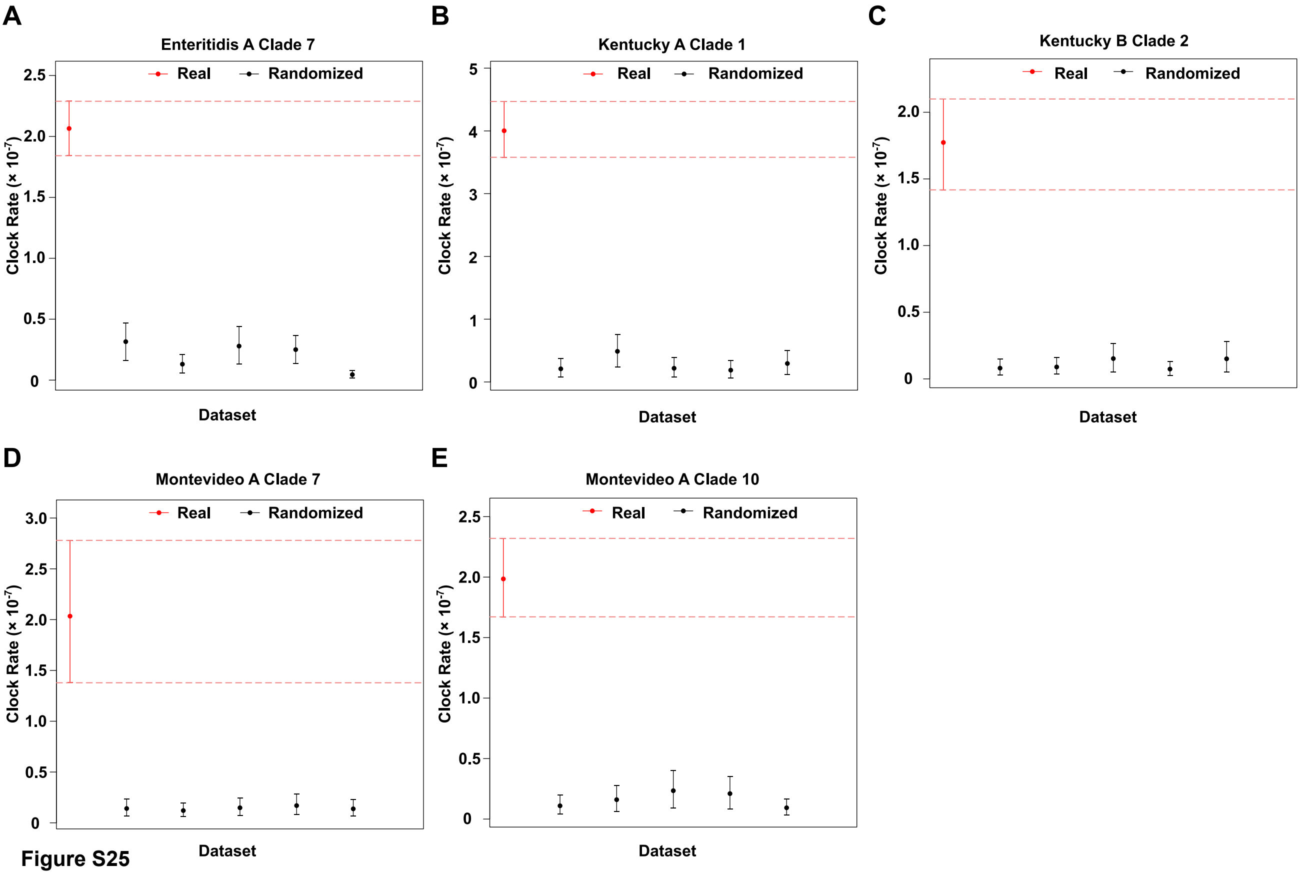
A
Enteritidis A Clade 7
Real
Randomized
Clock Rate (× 10^{-7})
2.5
2.0
1.5
1.0
0.5
0
Dataset
B
Kentucky A Clade 1
Real
Randomized
Clock Rate (× 10^{-7})
5
4
3
2
1
0
Dataset
C
Kentucky B Clade 2
Real
Randomized
Clock Rate (× 10^{-7})
2.0
1.5
1.0
0.5
0
Dataset
D
Montevideo A Clade 7
Real
Randomized
Clock Rate (× 10^{-7})
3.0
2.5
2.0
1.5
1.0
0.5
0
Dataset
E
Montevideo A Clade 10
Real
Randomized
Clock Rate (× 10^{-7})
2.5
2.0
1.5
1.0
0.5
0
Dataset

Figure S25
