# Supplementary material for: Salmonella associated with agricultural animals exhibit diverse evolutionary rates and show evidence of recent clonal expansion
Source: mBio. 2024 Sep 17;15(10):e01913-24. doi: 10.1128/mbio.01913-24 (PMC11492988; doi:10.1128/mbio.01913-24)
Supplement: Additional experimental details — Detailed experimental procedures and legends to supplemental figures. [file mbio.01913-24-s0004.docx]

**Supplementary Information.** Extended version of the Materials and Methods section of the main text.

**MATERIALS AND METHODS**

**Identification of candidate SNP clusters and singletons.** As part of the automated real-time surveillance analyses, the National Center for Biotechnology Information (NCBI) Pathogen Detection (PD) project (<https://www.ncbi.nlm.nih.gov/pathogens/>) clusters all isolates in its database into single nucleotide polymorphism (SNP) clusters by implementing single-linkage clustering with a cutoff of 25 whole-genome Multi-Locus Sequence Typing (wgMLST) alleles. Isolates not assigned to any SNP clusters are designated as singletons. On August 31, 2021, we obtained the metadata for all *Salmonella enterica* isolates available in the database to identify candidate SNP clusters and singletons for each serovar. Specifically, for a given serovar (e.g., Montevideo), we identified isolates with the serovar name and/or the corresponding antigenic formula (e.g., I 6,7,14:g,m,[p],s:[1,2,7]) displayed in the “computed_types” column within the metadata. SNP clusters with ≥ 10% of their isolates identified for a given serovar were considered candidate SNP clusters of that serovar, while any identified isolates not yet assigned to a SNP cluster were considered candidate singletons of the serovar.

***De novo* assembly, quality check, and in silico serotyping.** For detailed phylogenetic analyses, a set of representative isolates was selected for each of the seven serovars studied. For a given serovar, this included all singletons and the isolates that had the fewest contigs within their respective SNP cluster. Genome assemblies of the representative isolates were acquired by directly download from the NCBI PD database. In cases where the genome assemblies were not yet available, raw sequence reads were acquired from the NCBI Sequence Read Archive (SRA; <https://www.ncbi.nlm.nih.gov/sra/>) and *de novo* assembled using SKESA v 2.4.0 (1). To optimize the accuracy and precision of the phylogeny reconstruction, all assemblies were checked for quality using QUAST v 5.1.0rcl (2), with the criteria of having (i) fewer than 500 contigs, (ii) a Q50 value greater than 5,000, and (iii) a genome size between 4 and 6 Mb. Assemblies that failed to meet all these criteria were removed (for singletons) or iteratively replaced with assemblies from the same SNP cluster until a suitable assembly was found (for SNP clusters). In addition, the serotype of the representative isolates was confirmed using a customized in silico serotyping workflow, which integrated SISTR v 1.1.1 (3), SeqSero2 v 1.2.1 (4), and additional methods such as those based on additional genetic markers or phylogenetic affinity (Fig. S24; see the figure legend for detailed procedures).

**Reference-free core SNP variant calling and phylogenetic analyses.** The framework described in Chen et al. (5) was used to obtain a comprehensive understanding of the phylogeny of each serovar. Specifically, for a given serovar, phylogenetic analyses were performed using all its representative isolates, along with a compilation of reference isolates that represent 285 distinct *Salmonella enterica* subsp. *enterica* serovars and an additional *Salmonella enterica* subspecies (*indica*) as the outgroup for rooting the tree (6). kSNP v 3.1 (7) was used to create the core SNP alignment among all representative and reference isolates. An optimal k-mer size of 19, estimated using Kchooser, was specified. This core SNP alignment was then used to reconstruct a maximum likelihood phylogeny was reconstructed with FastTree v 2.1.10 (8), specifying the GTR+CAT model and incorporating the gamma discrete model with 20 rate categories for likelihood calculation (9, 10). The reliability of tree topology was assessed by computing local support values using the Shimodaira-Hasegawa test (11) with 1,000 resamples. The phylogeny was visualized and annotated using the Interactive Tree Of Life (iTOL; 11).

**Identification of phylogenetic groups and clades within serovars.** Overall phylogenies were used to infer whether a given serovar is polyphyletic, meaning that it comprises isolates with identical antigenic formula from different lineages that do not share the same most recent common ancestor (MRCA), based on the tree topology and local support values. If a given serovar was polyphyletic, distinct phylogenetic groups, either monophyletic or paraphyletic, were identified and alphabetically designated from A onwards based on the total number of isolates (e.g., Montevideo A, Montevideo B, etc.), with the highest number corresponding to A. Singletons of the serovar, which did not cluster with any phylogenetic groups, were identified and numbered in order (e.g., Enteritidis S1, Enteritidis S2, etc.). To enable comparison between different serovars, an additional round of reference-free core SNP variant calling and maximum-likelihood phylogeny reconstruction were performed using kSNP3 and FastTree, respectively, to include selected isolates from each phylogenetic group and standalone singleton of any serovars of interest and the set of reference isolates, as described previously in the section “Reference-free core SNP variant calling and phylogenetic analyses”.

For a given serovar, the downstream analyses focused on the phylogenetic group(s) comprising the majority of isolates. To further assess the relatedness of isolates within a specific phylogenetic group, representative isolates within the group and reference isolates of closely related serovar(s), manually selected based on the overall phylogeny, were used to perform a reference-free core SNP variant calling, followed by reconstruction of maximum-likelihood phylogeny, as detailed in the “Reference-free core SNP variant calling and phylogenetic analyses” section. Bayesian analysis of population structure was subsequently performed, using the fastbaps v 1.0.6 package (13) in the R Statistical Programming Environment (R) v 4.1.1 (14), to divide the phylogenetic group into three nested levels of clades based on the core SNP alignment and the reconstructed phylogeny. This analysis allowed for the partitioning of an existing phylogeny into clades, maximizing the marginal likelihood of a Dirichlet process mixture model.

To determine appropriate clades for downstream analyses, clades at different levels within each phylogenetic group were manually examined. For a given phylogenetic group, if more than one clade with > 100 isolates were present at a particular level, each of these clades was treated as a clade of interest and no further inspections were conducted on lower-level clades. Conversely, if only one clade contained > 100 isolates at a given level, further inspections were carried out on clades at one level below that were nested within this biggest clade. For each identified clade of interest, mlst v 2.16.1 (14; https://github.com/tseemann/mlst) was utilized to determine the sequence type of each SNP cluster and singleton from within.

**Assessment of genetic diversity.** The genetic diversity of each clade was determined by calculating the Simpson’s Diversity Index, wherein each of the SNP clusters and singletons were treated as a distinct “subtype”. The resulting diversity index was then subtracted from 1 to derive a “Clonal Index”, which indicates the degree of clonality of a given clade.

**Isolation date assignment for the isolates.** The date of isolation for each isolate was determined based on the “collection_date” column (comprising collection year, month, and day) and the “target_creation_date” column (comprising creation year, month, and day) of the metadata. Specifically, for isolates with precise collection year, month, and day, we directly referred to the collection date as the date of isolation; for isolates with collection year and month but with missing data for collection day, we used the creation day if the collection year and month matched the creation year and month, and we assumed 16th of the collection month if otherwise; for isolates with collection year but with missing data for collection month and day, we used the creation month and day if the collection year matched the creation year, and we assumed July 1 of the collection year if otherwise; for isolates with missing data for collection year, month, and day, we directly referred to the creation date as the date of isolation.

**Identification of clades associated with animal or non-animal, natural environmental sources.** Two sets of case-insensitive keywords were created for each of the four major agricultural animals in the US, namely cattle, swine, chicken, and turkey, by manual inspection of the 6,348 and 160 unique expressions in the “isolation_source” (e.g., “Feces [Bos taurus]”) and “host” (e.g., “Gallus gallus”) columns, respectively, within the metadata that encompassed all non-human *Salmonella* *enterica* isolates available in the NCBI PD database (accessed 31 August 2021). Within each column, unique expressions (e.g., “Bos taurus”, “Gallus gallus”) that indicated an association with a given animal were added to the keyword list for that animal. For instance, “Feces [Bos taurus]” was added to the list for cattle, while “Tissue [Gallus gallus]” was added to the list for chicken. As such, the unique expressions in each column were thus classified into four source categories, each corresponding to one of the four animals, and an additional “other” category was created to encompass unique expressions that did not fit into any of the four animal categories, which occasionally represented natural environmental sources unrelated to major agricultural animals (hereafter referred to as environmental sources). Unique expressions that indicated (i) cooked products (e.g., “Chicken meat strips, cooked”), (ii) highly processed products (e.g., “diced ham sold frozen”), (iii) products with multiple major ingredients (e.g., “coleslaw”), (iv) dairy and egg products (e.g., “chicken egg”, and/or (v) products for pets (e.g., “chicken jerky pet treat”) were not assigned to any animal source categories. The final classification of unique expressions was subsequently used to associate non-human *Salmonella enterica* isolates in the NCBI PD database with different animal or environmental source categories.

To identify clades associated with specific animal or environmental sources across different serovars, clades with > 100 non-human isolates were assessed for over- or underrepresentation of isolates collected from each animal or environmental source category. Specifically, the odds ratio of isolates associated with a specific source category was calculated for each clade by dividing the odds of isolates assigned to the category among all non-human isolates in the clade by the odds of isolates assigned to the category among all non-human isolates available in the NCBI PD database not belonging to the clade. Based on the odds ratios, the one-sided Fisher’s exact test was performed, with Benjamini-Hochberg (BH) correction for multiple testing, to determine whether a clade showed a significant overrepresentation (for odds ratios > 1) or underrepresentation (for odds ratios < 1) of isolates belonging to a source category. A given animal or environmental source category was considered overrepresented among isolates within a given clade provided an odds ratio > 5 and a BH-corrected *P*-value < 0.05 while underrepresented thereof provided an odds ratio < 0.2 and a BH-corrected *P*-value < 0.05. Unless otherwise specified, clades with 100 or fewer non-human isolates were not associated with any animal or environmental source categories due to their relatively small sample size.

Notably, ascertainment biases due to the oversampling of isolates by regulatory agencies during traceback investigations could introduce uncertainties regarding the associations of *Salmonella* clades with source categories determined using the approach described above, as they might affect the accurate calculation of odds ratios and *P*-values. Hence, it is imperative to conduct a post hoc evaluation of the potential impacts of these ascertainment biases on our analyses. Importantly, traceback investigations leading to oversampling of time- and source-associated isolates within a specific clade could be pinpointed by searching for the so-called “high outliers”, referring to the months signified by disproportionately high proportions of source-associated isolates within the clade compared to all *Salmonella* isolates from the respective source category. Therefore, for each clade associated with animal or environmental sources, we calculated the proportion of source-associated isolates for each month, and we identified the pertinent high outliers by applying the interquartile range rule to the proportions. Using 1% of the total source-associated isolates within the clade as the threshold, months with too few source-associated isolates within the clade were not included for identifying high outliers. Subsequently, the association between the clade and its respective source category was confirmed by conducting the identical analysis (i.e., odds ratio calculations and the subsequent Fisher’s exact tests) with the high outliers excluded. The odds ratios and *P*-values of the analysis after removal of high outliers were reported.

**Assessment of public health significance.** The likelihood for causing human illnesses was considered as a primary measure for inferring public health significance. Hence, to determine whether isolates within each phylogenetic group differ in their public health significance, we employed epidemiological evidence to compute the odds ratio of human isolates for each clade (i.e., the odds of human isolates among all isolates in the clade divided by the odds of human isolates among all *Salmonella* isolates not belonging to the clade), followed by using the one-tailed Fisher’s exact test, along with the Benjamini-Hochberg (BH) method for multiple correction, to determine statistical significance. Notably, although 81% of the isolates in the NCBI PD database originated from the US or the UK, the proportion of human isolates among all uploaded isolates varied considerably between the two countries (70% for the US and 93% for the UK). As a result, the odds ratio and BH-corrected *P*-value were calculated for each clade based on isolates collected from either the US or the UK, whichever represented the predominant country. Clades that had an odds ratio > 2 with BH-corrected *P*-value < 0.05 were deemed to exhibit an enrichment for human isolates, signifying an enhanced public health significance.

Importantly, the NCBI PD database may contain ascertainment biases due to the addition of human isolates derived from outbreaks. To mitigate the potential impacts of such biases on our analysis, we conducted a post hoc evaluation of the clades showing enrichment for human isolates, leveraging the fact that outbreaks often result in an influx of human isolates within a specific clade over a short period of time. Specifically, we defined the so-called “high outliers” as the months that showed disproportionately high proportions of human isolates within the clade compared to all human isolates. For each clade of interest, we calculated the proportion of human isolates for each month, based on isolates collected from the predominant country, and we identified the pertinent high outliers by applying the interquartile range rule to the proportions. Using 1% of the total human isolates within the clade as the threshold, months with too few human isolates within the clade were not included for identifying high outliers. Subsequently, we confirmed the enrichment for human isolates within the clade by conducting the identical analysis (i.e., odds ratios calculations and the subsequent Fisher’s exact tests) with the high outliers excluded. The odds ratios and *P*-values of the analysis after removal of high outliers were reported.

**In silico analysis of antimicrobial resistance.** To assess the potential of antimicrobial resistance (AMR) and multidrug resistance (MDR; defined as acquired non-susceptibility to at least one agent in three or more antimicrobial classes) of each clade, the presence/absence data for the highly curated AMR determinants (i.e., genes or point mutations) from the Pathogen Detection Reference Gene Catalog (https://www.ncbi.nlm.nih.gov/pathogens/refgene) was extracted for each isolate from the metadata. Following this, each distinct AMR determinant was linked to one or more drug classes defined by the Comprehensive Antibiotic Resistance Database (CARD; 15), and the number of drug classes to which resistance was likely conferred was determined for each isolate, based on the presence of AMR determinants in the genome. Isolates possessing AMR determinants responsible for at least three CARD drug classes were deemed putative MDR isolates.

**Evolutionary analysis.** Clade exhibiting an overrepresentation of isolates associated with any of the five source categories were selected as the focus of the evolutionary analysis. For each clade, this aimed to (i) estimate important evolution parameters, such as the time to most recent common ancestor (TMRCA) and substitution rate, and (ii) reconstruct the past population dynamics and time-scaled phylogeny. For each clade, 100 representative isolates were selected via stratified random sampling based on the collection year. The paired-end raw sequence reads of the representative isolates were downloaded from SRA and pre-processed using fastp v 0.23.2 (17) to (i) remove reads with over 40% unqualified bases, which were defined as bases with a phred score < 15 and/or a length < 15 bp, and (ii) detect and trim adaptors using a per read overlap analysis. A reference-based variant calling was then performed using Snippy v 4.3.6 (https://github.com/tseemann/snippy) to generate a core genome alignment among representative isolates, taking the pre-processed sequence reads as input and a closed genome or high-quality genome assembly as the reference genome for mapping reads. Recombination sites within the core genome alignment were identified and removed using Gubbins v 3.1.2 (18), and the variant sites of the filtered core genome alignment were extracted using snp-sites v 2.5.1 (19).

To verify the suitability of representative isolates for the evolutionary analysis of a given clade, the date-randomization test (DRT) was performed to assess the molecular-clock assumption, which determined whether the sampling dates of the representative isolates provided sufficient temporal signal for measuring evolutionary changes (19). Briefly, BEAUti v 2.5.2 (21) was used to generate an XML configuration file for the original dataset, specifying the HKY substitution model, the strict molecular clock, and the coalescent constant population size. An ascertainment bias correction was manually incorporated in the XML file to account for the use of only variant sites (https://groups.google.com/g/beast-users/c/QfBHMOqImFE). According to previous studies, the initial clock rate, measured in substitutions/site/year, was set to 4.8 × 10^7^ for Kentucky B (22), 2.2 × 10^7^ for Enteritidis A (23), 7.2 × 10^7^ for Cerro A (24), and 4.1 × 10^7^ for Reading A as well as Reading C (25). The initial clock rates for Montevideo A, Kentucky A, Dublin, and Infantis A remained poorly documented and were thus set to the average rate of bacteria, which is 2.1 × 10^7^ substitutions/site/year. Five datasets with randomized dates (and their corresponding XML files) were generated using the TipDatingBeast v 1.1-0 package (26) in R. In these datasets, the sampling dates of the representative isolates were randomly reassigned, which disrupted the association between substitutions and time. BEAST v 2.5.2 (27) was then employed to estimate the substitution rate of both the original and date-randomized datasets, and the results were visualized using the TipDatingBeast v 1.1-0 package. The DRT was deemed successfully passed if the estimated substitution rate of the original dataset did not fall within the 95% credible intervals of substitution rate estimated for the date-randomized datasets. Based on this criterion, all datasets generated for the clades associated with animal or environmental sources were deemed to have sufficient temporal signal for measuring evolutionary changes, which justified further evolutionary analysis using the Bayesian framework (Fig. S25).

bModelTest v 1.1.2 (28) was employed to implement a reversible jump Markov Chain Monte-Carlo algorithm (rjMCMC) on selected clades from various serovars. This aimed to determine (i) the most suitable nucleotide substitution model for the clades, (ii) whether to include Gamma rate heterogeneity, and (iii) whether the lognormal relaxed molecular clock would be more appropriate compared to the strict molecular clock. The substitution model that received the highest posterior support (i.e., the amount of time the Markov chain spent on a given model relative to other competing models) was deemed the best-fit model; the Gamma rate heterogeneity was considered appropriate to incorporate if it was included in the Markov chain for > 50% of the time spent; and the lognormal relaxed molecular clock was considered to have superior performance if the rate coefficient of variation exceeded 0.1. The analysis revealed that the substitution model number 23, which assumes identical rates of nucleotide change between AC and GT as well as between AG and CT, was the best-fit model for all but one selected clade. Therefore, this model was used for all clades in the subsequent analyses (Table S2). Furthermore, the incorporation of Gamma rate heterogeneity and lognormal relaxed molecular clock was recommended for all selected clades (Table S2), and these settings were thus applied to all clades in the subsequent analyses.

To identify the optimal population model for characterizing the past population dynamics of different clades, a subset of clades from various serovars were selected to compare three candidate models: (i) coalescent constant, (ii) coalescent exponential growth, and (iii) coalescent Bayesian Skyline. Specifically, for each selected clade, we performed three independent analysis using the stepping stone sampling approach (29) with different random seeds to estimate the marginal likelihood for each of the three candidate models. A chain length of 4 million generations and 50 steps were specified for all stepping stone analyses. The optimal population model for each selected clade was determined by calculating the Bayes factor (i.e., the ratio of estimated marginal likelihood) among candidate models and comparing between each pair of models using the following criteria: (i) 3.2 < BF < 10 indicated substantial evidence in favor of the numerator model, (ii) 10 < BF < 100 indicated strong evidence in favor of the numerator model, and (iii) BF > 100 indicated decisive evidence in favor of the numerator model (https://www.beast2.org/2021/11/01/hypothesis-testing.html). The coalescent Bayesian Skyline model was found to be the optimal population model for all selected clades, and it was thus applied to all clades in the subsequent analyses (Table S3).

To estimate the posterior probability distribution of the genealogical and demographic parameters associated with each clade, a Markov Chain Monte-Carlo algorithm (MCMC) was run using BEAST v 2.5.2 (27) for three independent times with different random seeds. The chain length was set to at least 100 million generations, with sampling performed every 10,000 generations. The log files from the three independent runs for each clade were combined using LogCombiner v 2.5.2 (30), with the first 10% iterations of each MCMC chain removed as burn-ins. The adequate mixing of each independent MCMC chain and the agreement across all three chains were manually confirmed using Tracer v 1.7.2 (31) by checking the effective sample sizes (ESSs) of run statistics and comparing the patterns of the individual traces. The estimate of a given parameter was deemed dependable if (i) the ESS produced was > 100 for each independent MCMC chain and > 200 when combining all three chains, and (ii) the traces for different MCMC chains were overlapped and no distinct trends could be identified. The tree files obtained from the three independent runs for each clade were combined using LogCombiner v 2.5.2, after discarding the first 10% iterations of each MCMC chain as burn-ins. The resulting tree file was then analyzed using TreeAnnotator v 2.5.2 (32) to produce a maximum clade credibility tree, which represented the time-scaled phylogeny. This tree was visualized and edited using FigTree v 1.4.4 (33) as well as R packages ggtree v 3.2.1 (34, 35) and treeio v 1.18.1 (36, 37). To reveal the past population dynamics for each clade, a Bayesian Skyline plot was reconstructed in Tracer v 1.7.2 using the combined log and tree files.

**Identification of clonal sub-clades within clades associated with animal sources.** To ascertain the presence, within the animal-associated clades, of clonal sub-clades that showed an enhanced likelihood of causing human illnesses or adapting to animal sources, the reference-based core SNP alignment and time-scaled phylogeny of each clade were utilized to identify clusters of isolates with low genetic distances (< 5% of the total number of core SNPs identified among the 100 representative isolates of the clade). Subsequently, the clusters were matched with relevant metadata to determine their association with human or animal host adaptation. Clonal sub-clades enriched for isolates from human or animal sources were identified based on a set of criteria predefined with respect to (i) number of isolates, (ii) posterior probability for branch support, (iii) maximum pairwise genetic distance, and (iv) changes in the proportion of human- or animal-associated isolates as compared to the baseline level (see Supplementary Information 1 for details).

**Identification of clonal sub-clades within clades associated with animal sources.** To ascertain the presence, within the animal-associated clades, of clonal sub-clades that showed an enhanced likelihood of causing human illnesses or adapting to animal sources, the reference-based core SNP alignment and time-scaled phylogeny of each clade were utilized to identify clusters of isolates with low genetic distances (i.e., clonal sub-clades). Subsequently, the clonal sub-clades were matched with relevant metadata and epidemiology data to determine their association with human or animal host adaptation. Briefly, for each clade, we used ClusterPicker v 1.2.5 (38) to partition the time-scaled phylogeny into clusters, such that each cluster had a posterior probability > 0.8 and a maximum pairwise genetic distance < 5% of the total number of substitutions in the core SNP alignment. Clusters with > 10 of the 100 representative isolates were considered as clonal sub-clades within the clade. ClusterMatcher v 1.2.7 (39) was then employed to annotate each clonal sub-clade with information regarding source category, collection dates, geographical location, and AMR/MDR profiles. Clonal sub-clades enriched for isolates from human or animal sources were identified as the ones that showed a proportion of human or animal isolates at least 20 percentage points higher than that of the baseline population, which comprised all isolates not belonging to any of the clonal sub-clades.

**Statistical analysis.** A series of linear regression models were developed to examine the potential impact of various populational and evolutionary factors on the likelihood of a given clade causing human illnesses. Each model utilized the odds ratio between human and non-human isolates as the response variable and one specific factor of interest (i.e., association with animal or environmental sources, TMRCA, source category, MDR level, or clonality level) as the explanatory variable. All models were built using the stats v 4.2.1 package in R (13). The significance threshold for all statistical tests was set to a *P*-value of 0.05.

**Supplemental Figure Legends**

**Figure S1.** **Maximum Likelihood Phylogeny for *S.* Montevideo.** *S.* Montevideo is a polyphyletic serovar, comprising four distinct phylogenetic groups (Montevideo A-D). The maximum likelihood phylogeny was reconstructed based on 23,296 core SNPs identified among isolates representing each of the SNP clusters and singletons belonging to *S.* Montevideo, as well as 285 unique *Salmonella enterica* subsp. *enterica* serovars and one additional *Salmonella enterica* subspecies (*indica*) as the outgroup. The average pairwise number of nucleotide substitutions per site is used to report branch lengths. *Salmonella enterica* subsp. *indica*, the outgroup, is used for rooting the tree, and the outgroup branch was shortened (1:100) for improved visualization of the tree topology. The Shimodaira-Hasegawa (SH) test with 1,000 resamples is used to assess the clustering confidence. The most recent common ancestor (MRCA) of each phylogenetic group is labeled with a red star and the corresponding bootstrap support value.

**Figure S2. Maximum Likelihood Phylogeny for *S.* Enteritidis.** *S.* Enteritidis is a polyphyletic serovar, comprising six distinct phylogenetic groups (Enteritidis A-F) and two singletons (Enteritidis S1 and S2). The maximum likelihood phylogeny was reconstructed based on 22,078 core SNPs identified among isolates representing each of the SNP clusters and singletons belonging to *S.* Enteritidis, as well as 285 unique *Salmonella enterica* subsp. *enterica* serovars and one additional *Salmonella enterica* subspecies (*indica*) as the outgroup. The average pairwise number of nucleotide substitutions per site is used to report branch lengths. *Salmonella enterica* subsp. *indica*, the outgroup, was used for rooting the tree, and the outgroup branch was shorted (1:100) for improved visualization of the tree topology. The Shimodaira-Hasegawa (SH) test with 1,000 resamples is used to assess the clustering confidence. The most recent common ancestor (MRCA) of each phylogenetic group is labeled with a red star and the corresponding bootstrap support value.

**Figure S3. Maximum Likelihood Phylogeny for *S.* Kentucky.** *S.* Kentucky is a polyphyletic serovar, comprising two distinct phylogenetic groups (Kentucky A and B) and two singletons (Kentucky S1 and S2). The maximum likelihood phylogeny was reconstructed based on 10,722 core SNPs identified among isolates representing each of the SNP clusters and singletons belonging to *S.* Kentucky, as well as 285 unique *Salmonella enterica* subsp. *enterica* serovars and one additional *Salmonella enterica* subspecies (*indica*) as the outgroup. The average pairwise number of nucleotide substitutions per site is used to report branch lengths. *Salmonella enterica* subsp. *indica*, the outgroup, was used for rooting the tree, and the outgroup branch was shortened (1:100) for improved visualization of the tree topology. The Shimodaira-Hasegawa (SH) test with 1,000 resamples is used to assess the clustering confidence. The most recent common ancestor (MRCA) of each phylogenetic group is labeled with a red star and the corresponding bootstrap support value.

**Figure S4. Maximum Likelihood Phylogeny for *S.* Dublin.** *S.* Dublin is a monophyletic serovar, comprising only one phylogenetic group. The maximum likelihood phylogeny was reconstructed based on 19,225 core SNPs identified among isolates representing each of the SNP clusters and singletons belonging to *S.* Dublin, as well as 285 unique *Salmonella enterica* subsp. *enterica* serovars and one additional *Salmonella enterica* subspecies (*indica*) as the outgroup. The average pairwise number of nucleotide substitutions per site is used to report branch lengths. *Salmonella enterica* subsp. *indica*, the outgroup, was used for rooting the tree, and the outgroup branch was shortened (1:100) for improved visualization of the tree topology. The Shimodaira-Hasegawa (SH) test with 1,000 resamples is used to assess the clustering confidence. The most recent common ancestor (MRCA) of each phylogenetic group is labeled with a red star and the corresponding bootstrap support value.

**Figure S5. Maximum Likelihood Phylogeny for *S.* Reading.** *S.* Reading is a polyphyletic serovar, comprising three distinct phylogenetic groups (Reading A-C). The maximum likelihood phylogeny was reconstructed based on 23,215 core SNPs identified among isolates representing each of the SNP clusters and singletons belonging to *S.* Reading, as well as 285 unique *Salmonella enterica* subsp. *enterica* serovars and one additional *Salmonella enterica* subspecies (*indica*) as the outgroup. The average pairwise number of nucleotide substitutions per site is used to report branch lengths. *Salmonella enterica* subsp. *indica*, the outgroup, was used for rooting the tree, and the outgroup branch was shortened (1:100) for improved visualization of the tree topology. The Shimodaira-Hasegawa (SH) test with 1,000 resamples is used to assess the clustering confidence. The most recent common ancestor (MRCA) of each phylogenetic group is labeled with a red star and the corresponding bootstrap support value.

**Figure S6.** **Maximum Likelihood Phylogeny for *S.* Cerro.** *S.* Cerro is a polyphyletic serovar, comprising five distinct phylogenetic groups (Cerro A-E) and one singleton (Cerro S1). The maximum likelihood phylogeny was reconstructed based on 19,591 core SNPs identified among isolates representing each of the SNP clusters and singletons belonging to *S.* Cerro, as well as 285 unique *Salmonella enterica* subsp. *enterica* serovars and one additional *Salmonella enterica* subspecies (*indica*) as the outgroup. The average pairwise number of nucleotide substitutions per site is used to report branch lengths. *Salmonella enterica* subsp. *indica*, the outgroup, was used for rooting the tree, and the outgroup branch was shortened (1:100) for improved visualization of the tree topology. The Shimodaira-Hasegawa (SH) test with 1,000 resamples is used to assess the clustering confidence. The most recent common ancestor (MRCA) of each phylogenetic group is labeled with a red star and the corresponding bootstrap support value.

**Figure S7.** **Maximum Likelihood Phylogeny for *S.* Infantis.** *S.* Infantis is a polyphyletic serovar, comprising two distinct phylogenetic groups (Infantis A and B) and one singleton (Infantis S1). The maximum likelihood phylogeny was reconstructed based on 23,107 core SNPs identified among isolates representing each of the SNP clusters and singletons belonging to *S.* Infantis, as well as 285 unique *Salmonella enterica* subsp. *enterica* serovars and one additional *Salmonella enterica* subspecies (*indica*) as the outgroup. The average pairwise number of nucleotide substitutions per site is used to report branch lengths. *Salmonella enterica* subsp. *indica*, the outgroup, was used for rooting the tree, and the outgroup branch was shortened (1:100) for improved visualization of the tree topology. The Shimodaira-Hasegawa (SH) test with 1,000 resamples is used to assess the clustering confidence. The most recent common ancestor (MRCA) of each phylogenetic group is labeled with a red star and the corresponding bootstrap support value.

**Figure S8. Maximum Likelihood Phylogeny for Enteritidis A.** Enteritidis A is a paraphyletic group comprising 10 clades, one of which is associated with chicken-related sources (clade 7). The maximum likelihood phylogeny was reconstructed based on 34,673 core SNPs identified among isolates representing each of the SNP clusters and singletons from Enteritidis A, along with closely related serovars (i.e., *S.* Gallinarum, *S.* Inverness, *S.* Abony, *S.* Stanley, *S.* Schleissheim, *S.* Paratyphi B, *S.* Berta, and *S.* Dublin) and Enteritidis B as the outgroup. The tree was rooted using the outgroup, and the clustering confidence was assessed using the Shimodaira-Hasegawa (SH) test with 1,000 resamples. Branch lengths are reported as the average pairwise number of nucleotide substitutions per site. Color shades for the internal color strip indicate different clades, while black corresponds to the outgroup and closely related serovars. All 25 *S.* Gallinarum isolates available in NCBI Pathogen Detection (accessed 31 August 2021) fell within Enteritidis A and formed a cluster (shaded in grey) between Enteritidis A clades 1 and 2. Red stars followed by bootstrap values at internal nodes are used to describe the most recent common ancestor (MRCA) of the four clades with > 100 isolates. The outer color strip represents the sequence type (ST) of the representative isolates. “Minor STs” represent STs accounting for ≤ 10 representative isolates. “Undetermined” designates isolates that could not be assigned to a ST due to either (i) novel combination of known alleles, (ii) identification of novel alleles, (iii) partial match to known alleles, (iv) missing alleles, or (v) identification of multiple alleles. Stacked bars show the number of human and non-human isolates collected from US (red: human isolates; blue: non-human isolates) or UK (light red: human isolates; light blue: non-human isolates) for each SNP cluster. The pie chart exhibits the distribution of non-human isolates across five isolation source categories (i.e., cattle, swine, chicken, turkey, and other) for the clade with >100 non-human isolates (connected to the MRCA of the respective clade). Source categories that are over- or underrepresented within the clade are indicated in red font.

**Figure S9. Maximum Likelihood Phylogeny for Kentucky A.** Kentucky A is a monophyletic group comprising four clades, one of which is associated with chicken-related sources (clade 1). The maximum likelihood phylogeny was reconstructed based on 25,334 core SNPs identified among isolates representing each of the SNP clusters and singletons from Kentucky A, along with Kentucky S1 and *S.* Agona as the outgroup. The tree was rooted using the outgroup, and the outgroup branch was shortened (1:100) for improved visualization of the tree topology. The clustering confidence was assessed using the Shimodaira-Hasegawa (SH) test with 1,000 resamples. Branch lengths are reported as the average pairwise number of nucleotide substitutions per site. Color shades for the internal color strip indicate different clades, while black corresponds to the outgroup. Red stars followed by bootstrap values at internal nodes are used to describe the most recent common ancestor (MRCA) of the two clades with > 100 isolates. The outer color strip represents the sequence type (ST) of the representative isolates. “Minor STs” represents the group of STs accounting for ≤ 10 representative isolates. “Undetermined” designates isolates that could not be assigned to a ST due to either (i) novel combination of known alleles, (ii) identification of novel alleles, (iii) partial match to known alleles, (iv) missing alleles, or (v) identification of multiple alleles. Stacked bars show the number of human and non-human isolates collected from US (red: human isolates; blue: non-human isolates) or UK (light red: human isolates; light blue: non-human isolates) for each SNP cluster. The pie chart exhibits the distribution of non-human isolates across five isolation source categories (i.e., cattle, swine, chicken, turkey, and other) for the clade with > 100 non-human isolates (connected to the MRCA of the respective clade). Source categories that are over- or underrepresented within the clade are indicated in red font.

**Figure S10.** **Maximum Likelihood Phylogeny for Kentucky B.** Kentucky B is a monophyletic group comprising six clades, one of which is associated with cattle-related sources (clade 2). The maximum likelihood phylogeny was reconstructed based on 27,624 core SNPs identified among isolates representing each of the SNP clusters and singletons from Kentucky B, along with *S.* Corvallis as the outgroup. The tree was rooted using the outgroup, and the outgroup branch was shortened (1:1,000) for improved visualization of the tree topology. The clustering confidence was assessed using the Shimodaira-Hasegawa (SH) test with 1,000 resamples. Branch lengths are reported as the average pairwise number of nucleotide substitutions per site. Color shades for the internal color strip indicate different clades, while black corresponds to the outgroup. Red stars followed by bootstrap values at internal nodes are used to describe the most recent common ancestor (MRCA) of the two clades with > 100 isolates. The outer color strip represents the sequence type (ST) of the representative isolates. “Minor STs” represent STs accounting for ≤ 10 representative isolates. “Undetermined” designates isolates that could not be assigned to a ST due to either (i) novel combination of known alleles, (ii) identification of novel alleles, (iii) partial match to known alleles, (iv) missing alleles, or (v) identification of multiple alleles. Stacked bars show the number of human and non-human isolates collected from US (red: human isolates; blue: non-human isolates) or UK (light red: human isolates; light blue: non-human isolates) for each SNP cluster. The pie chart exhibits the distribution of non-human isolates across five isolation source categories (i.e., cattle, swine, chicken, turkey, and other) for the clade with > 100 non-human isolates (connected to the MRCA of the respective clade). Source categories that are over- or underrepresented within the clade are indicated in red font.

**Figure S11.** **Maximum Likelihood Phylogeny for *S.* Dublin (as a Monophyletic Group).** Dublin is a monophyletic group comprising 16 clades, one of which is associated with cattle-related sources (clade 2-3). The maximum likelihood phylogeny was reconstructed based on 22,059 core SNPs identified among isolates representing each of the SNP clusters and singletons from Dublin, along with a closely related serovar (i.e., *S.* Enteritidis) and *S.* Gallinarum as the outgroup. The tree was rooted using the outgroup, and the clustering confidence was assessed using the Shimodaira-Hasegawa (SH) test with 1,000 resamples. Branch lengths are reported as the average pairwise number of nucleotide substitutions per site. Color shades for the internal color strip indicate different clades, while black corresponds to the outgroup. Red stars followed by bootstrap values at internal nodes are used to describe the most recent common ancestor (MRCA) of the three clades with > 100 isolates. The outer color strip represents the sequence type (ST) of the representative isolates. “Minor STs” represent STs accounting for ≤ 10 representative isolates. “Undetermined” designates isolates that could not be assigned to a ST due to either (i) novel combination of known alleles, (ii) identification of novel alleles, (iii) partial match to known alleles, (iv) missing alleles, or (v) identification of multiple alleles. Stacked bars show the number of human and non-human isolates collected from US (red: human isolates; blue: non-human isolates) or UK (light red: human isolates; light blue: non-human isolates) for each SNP cluster. The pie chart exhibits the distribution of non-human isolates across five isolation source categories (i.e., cattle, swine, chicken, turkey, and other) for the clade with > 100 non-human isolates (connected to the MRCA of the respective clade). Source categories that are over- or underrepresented within the clade are indicated in red font.

**Figure S12. Maximum Likelihood Phylogeny for Reading A.** Reading A is a monophyletic group comprising six clades, one of which is associated with turkey-related sources (clade 1-1-2). The maximum likelihood phylogeny was reconstructed based on 19,578 core SNPs identified among isolates representing each of the SNP clusters and singletons from Reading A, as well as closely related serovars (i.e., *S.* Goettingen, and *S.* Brandenburg) and *S.* Sandiego as the outgroup. The tree was rooted using the outgroup, and the outgroup branch was shortened (1:2) for improved visualization of the tree topology. The clustering confidence was assessed using the Shimodaira-Hasegawa (SH) test with 1,000 resamples. Branch lengths are reported as the average pairwise number of nucleotide substitutions per site. Color shades for the internal color strip indicate different clades, while black corresponds to the outgroup. Red stars followed by bootstrap values at internal nodes are used to describe the most recent common ancestor (MRCA) of the one clade with > 100 isolates. The outer color strip represents the sequence type (ST) of the representative isolates. “Minor STs” represent STs accounting for ≤ 10 representative isolates. “Undetermined” designates isolates that could not be assigned to a ST due to either (i) novel combination of known alleles, (ii) identification of novel alleles, (iii) partial match to known alleles, (iv) missing alleles, or (v) identification of multiple alleles. Stacked bars show the number of human and non-human isolates collected from US (red: human isolates; blue: non-human isolates) or UK (light red: human isolates; light blue: non-human isolates) for each SNP cluster. The pie chart exhibits the distribution of non-human isolates across five isolation source categories (i.e., cattle, swine, chicken, turkey, and other) for the clade with > 100 non-human isolates (connected to the MRCA of the respective clade). Source categories that are over- or underrepresented within the clade are indicated in red font.

**Figure S13. Maximum Likelihood Phylogeny for Reading B.** Reading B is a monophyletic group, comprising 12 clades. The maximum likelihood phylogeny was reconstructed based on 16,735 core SNPs identified among isolates representing each of the SNP clusters and singletons from Reading B, as well as a closely related serovar (i.e., *S.* Eastbourne) and *S.* Chester as the outgroup. The tree was rooted using the outgroup, and the outgroup branch was shortened (1:2) for improved visualization of the tree topology. The clustering confidence was assessed using the Shimodaira-Hasegawa (SH) test with 1,000 resamples. Branch lengths are reported as the average pairwise number of nucleotide substitutions per site. Color shades for the internal color strip indicate different clades, while black corresponds to the outgroup. Red stars followed by bootstrap values at internal nodes are used to describe the most recent common ancestor (MRCA) of the one clade with > 100 isolates. The outer color strip represents the sequence type (ST) of the representative isolates. “Minor STs” represent STs accounting for ≤ 10 representative isolates. “Undetermined” designates isolates that could not be assigned to a ST due to either (i) novel combination of known alleles, (ii) identification of novel alleles, (iii) partial match to known alleles, (iv) missing alleles, or (v) identification of multiple alleles. Stacked bars show the number of human and non-human isolates collected from US (red: human isolates; blue: non-human isolates) or UK (light red: human isolates; light blue: non-human isolates) for each SNP cluster.

**Figure S14.** **Maximum Likelihood Phylogeny for Reading C.** Reading C is a monophyletic group comprising five clades, one of which is associated with swine-related sources (clade 1-1-4). The maximum likelihood phylogeny was reconstructed based on 20,812 core SNPs identified among isolates representing each of the SNP clusters and singletons from Reading C, as well as a closely related serovar (i.e., *S.* I 4,[5],12:i:-) and *S.* Typhimurium as the outgroup. The tree was rooted using the outgroup, and the outgroup branch was shorted (1:2) for improved visualization of the tree topology. The clustering confidence was assessed using the Shimodaira-Hasegawa (SH) test with 1,000 resamples. Branch lengths are reported as the average pairwise number of nucleotide substitutions per site. Color shades for the internal color strip indicate different clades, while black corresponds to the outgroup. Red stars followed by bootstrap values at internal nodes are used to describe the most recent common ancestor (MRCA) of the one clade with > 100 isolates. The outer color strip represents the sequence type (ST) of the representative isolates. “Minor STs” represent STs accounting for ≤ 10 representative isolates. “Undetermined” designates isolates that could not be assigned to a ST due to either (i) novel combination of known alleles, (ii) identification of novel alleles, (iii) partial match to known alleles, (iv) missing alleles, or (v) identification of multiple alleles. Stacked bars show the number of human and non-human isolates collected from US (red: human isolates; blue: non-human isolates) or UK (light red: human isolates; light blue: non-human isolates) for each SNP cluster. The pie chart exhibits the distribution of non-human isolates across five isolation source categories (i.e., cattle, swine, chicken, turkey, and other) for the clade with > 100 non-human isolates (connected to the MRCA of the respective clade). Source categories that are over- or underrepresented within the clade are indicated in red font.

**Figure S15.** **Maximum Likelihood Phylogeny for Cerro A.** Cerro A is a monophyletic group comprising six clades, two of which are associated with cattle-related (clade 2) and swine (clade 3) sources, respectively. The maximum likelihood phylogeny was reconstructed based on 44,680 core SNPs identified among isolates representing each of the SNP clusters and singletons from Cerro A, as well as closely related serovars (i.e., *S.* Berkeley, and *S.* Ahuza) and *S.* Oslo as the outgroup. The tree was rooted using the outgroup, and the clustering confidence was assessed using the Shimodaira-Hasegawa (SH) test with 1,000 resamples. Branch lengths are reported as the average pairwise number of nucleotide substitutions per site. Color shades for the internal color strip indicate different clades, while black corresponds to the outgroup. Red stars followed by bootstrap values at internal nodes are used to describe the most recent common ancestor (MRCA) of the two clades with > 100 isolates. The outer color strip represents the sequence type (ST) of the representative isolates. “Minor STs” represent STs accounting for ≤ 10 representative isolates. “Undetermined” designates isolates that could not be assigned to a ST due to either (i) novel combination of known alleles, (ii) identification of novel alleles, (iii) partial match to known alleles, (iv) missing alleles, or (v) identification of multiple alleles. Stacked bars show the number of human and non-human isolates collected from US (red: human isolates; blue: non-human isolates) or UK (light red: human isolates; light blue: non-human isolates) for each SNP cluster. Pie charts exhibit the distribution of non-human isolates across five isolation source categories (i.e., cattle, swine, chicken, turkey, and other) for each clade with > 100 non-human isolates (connected to the MRCA of the respective clade). Source categories that are over- or underrepresented within a clade are indicated in red font.

**Figure S16.** **Maximum Likelihood Phylogeny for Infantis A.** Infantis A is a paraphyletic group comprising 16 clades, two of which are associated with swine-related (clade 1-2) and chicken-related (clade 1-3) sources, respectively. The maximum likelihood phylogeny was reconstructed based on 45,946 core SNPs identified among isolates representing each of the SNP clusters and singletons from Infantis A, as well as closely related serovars (i.e., *S.* Oritamerin, and *S.* Colindale) and *S.* Virchow as the outgroup. The tree was rooted using the outgroup, and the clustering confidence was assessed using the Shimodaira-Hasegawa (SH) test with 1,000 resamples. Branch lengths are reported as the average pairwise number of nucleotide substitutions per site. Color shades for the internal color strip indicate different clades, while black corresponds to the outgroup. *S.* Oritamerin isolates available in NCBI Pathogen Detection fell within Infantis A and formed a cluster (shaded in gray) between Infantis A clades 4-1 and 4-4. Red stars followed by bootstrap values at internal nodes are used to describe the most recent common ancestor (MRCA) of the four clades with > 100 isolates. The outer color strip represents the sequence type (ST) of the representative isolates. “Minor STs” represent STs accounting for ≤ 10 representative isolates. “Undetermined” designates isolates that could not be assigned to a ST due to either (i) novel combination of known alleles, (ii) identification of novel alleles, (iii) partial match to known alleles, (iv) missing alleles, or (v) identification of multiple alleles. Stacked bars show the number of human and non-human isolates collected from US (red: human isolates; blue: non-human isolates) or UK (light red: human isolates; light blue: non-human isolates) for each SNP cluster. Pie charts exhibit the distribution of non-human isolates across five isolation source categories (i.e., cattle, swine, chicken, turkey, and other) for each clade with > 100 non-human isolates (connected to the MRCA of the respective clade). Source categories that are over- or underrepresented within a clade are indicated in red font.

**Figure S17.** **Time-Scaled Phylogeny (with Bayesian Skyline Plot Insert) for Enteritidis A Clade 7.** The Bayesian time-scaled maximum clade credibility (MCC) phylogeny and Bayesian Skyline plot (BSP) were reconstructed based on core SNPs identified among 100 representative isolates within Enteritidis A clade 7; representative isolates were selected using time-stratified random sampling. The embedded BSP depicts the dynamics of the effective population size over time, with the y-axis representing the product of effective population size (*Ne*) and generation time (*τ*) and x-axis representing time in years. A bold line is used to display the median of *Neτ* across the complete time course, and the shaded area surrounding the line represents the 95% highest posterior density (HPD) interval. The MCC phylogeny next to the BSP represents time in years on the x-axis, and the branch lengths are reported in years. Red stars indicate the most recent common ancestor (MRCA) of sub-clades I-IV, and posterior probabilities of branch support are attached to the MRCAs as well as important subsequent ancestors. Leaf nodes are color-coded to denote the isolation source category of the representative isolates, with the category “other” including isolates not collected from sources related to human, cattle, swine, chicken, and turkey. The following information is displayed adjacent to the leaf nodes: (i) collection date, (ii) geographical location, and (iii) sequence type. The bar plot on the far-right displays, for each representative isolate, the number of antimicrobial resistance (AMR) classes, based on the presence of AMR determinants in the genome. Bars are color-matched with the leaf nodes, and a vertical red dashed line is overlaid to distinguish multidrug-resistant (MDR) isolates (i.e., isolates resistant to at least one agent in at least three drug classes) from the rest. Colored rectangles are overlaid on both the MCC phylogeny and the BSP to highlight important time blocks that involve changes in ***Neτ***. By cross-referencing the MCC phylogeny and the BSP, it appears that the ubiquitous sub-clade exhibits an increase in the occurrence of short branches and the density of coalescent events during the red time block (1970-1980), and an increase in the occurrence of long branches during the green time block (1980-2000), which corresponds well with the decrease and subsequent increase in *Neτ* shown in the BSP, respectively.

**Figure S18.** **Time-Scaled Phylogeny (with Bayesian Skyline Plot Insert) for Infantis A Clade 1-3.** The Bayesian time-scaled maximum clade credibility (MCC) phylogeny and Bayesian Skyline plot (BSP) were reconstructed based on core SNPs identified among 100 representative isolates within Infantis A clade 1-3; representative isolates were selected using time-stratified random sampling. The embedded BSP depicts the dynamics of the effective population size over time, with the y-axis representing the product of effective population size (*Ne*) and generation time (*τ*) and x-axis representing time in years. A bold line is used to display the median of *Neτ* across the complete time course, and the shaded area surrounding the line represents the 95% highest posterior density (HPD) interval. The MCC phylogeny next to the BSP represents time in years on the x-axis, and the branch lengths are reported in years. Red stars indicate the most recent common ancestor (MRCA) of sub-clades I and II, and posterior probabilities of branch support are attached to the MRCAs as well as important subsequent ancestors. Leaf nodes are color-coded to denote the isolation source category of the representative isolates, with the category “other” including isolates not collected from sources related to human, cattle, swine, chicken, and turkey. The following information is displayed adjacent to the leaf nodes: (i) collection date, (ii) geographical location, and (iii) sequence type. The bar plot on the far-right displays, for each representative isolate, the number of antimicrobial resistance (AMR) classes, based on the presence of AMR determinants in the genome. Bars are color-matched with the leaf nodes, and a vertical red dashed line is overlaid to distinguish multidrug-resistant (MDR) isolates (i.e., isolates resistant to at least one agent in at least three drug classes) from the rest. Colored rectangles are overlaid on both the MCC phylogeny and the BSP to highlight important time blocks that involve changes in *Neτ*. Cross-referencing the MCC phylogeny and the BSP suggests a clonal expansion undergone by sub-clade I. Specifically, sub-clade I exhibits an increase in the occurrence of short branches and the density of coalescent events during the red time block (2002-2007), followed by an increase in the occurrence of long branches during the green time block (2007-2010). These changes correspond well with the decrease and the subsequent increase in *Neτ* shown in the BSP, respectively.

**Figure S19.** **Time-Scaled Phylogeny (with Bayesian Skyline Plot Insert) for Cerro A Clade 2.** The Bayesian time-scaled maximum clade credibility (MCC) phylogeny and Bayesian Skyline plot (BSP) were reconstructed based on core SNPs identified among 100 representative isolates within Cerro A clade 2; representative isolates were selected using time-stratified random sampling. The embedded BSP depicts the dynamics of the effective population size over time, with the y-axis representing the product of effective population size (*Ne*) and generation time (*τ*) and x-axis representing time in years. A bold line is used to display the median of *Neτ* across the complete time course, and the shaded area surrounding the line represents the 95% highest posterior density (HPD) interval. The MCC phylogeny next to the BSP represents time in years on the x-axis, and the branch lengths are reported in years. The red star indicates the most recent common ancestor (MRCA) of sub-clade I, with the posterior probability of branch support next to it. Leaf nodes are color-coded to denote the isolation source category of the representative isolates, with the category “other” including isolates not collected from sources related to human, cattle, swine, chicken, and turkey. The following information is displayed adjacent to the leaf nodes: (i) collection date, (ii) geographical location, and (iii) sequence type. The bar plot on the far-right displays, for each representative isolate, the number of antimicrobial resistance (AMR) classes, based on the presence of AMR determinants in the genome. Bars are color-matched with the leaf nodes, and a vertical red dashed line is overlaid to distinguish multidrug-resistant (MDR) isolates (i.e., isolates resistant to at least one agent in at least three drug classes) from the rest. Colored rectangles are overlaid on both the MCC phylogeny and the BSP to highlight important time blocks associated with changes in *Neτ*. Cross-referencing the MCC phylogeny and the BSP suggests a clonal expansion undergone by sub-clade I. During the brown time block (1995-2005), there is an increase in the occurrence of short branches and the density of coalescent events following the MRCA of sub-clade I. These changes, however, may have been counteracted by other parts of the MCC phylogeny and hence not driving changes in *Ne*τ in the BSP. Nevertheless, upon entry into the green time block (2005-2010), there is an increase in the occurrence of long branches within sub-clade I, which corresponds well with the notable increase in *Ne*τ shown in the BSP.

**Figure S20.** **Time-scaled Phylogeny (with Bayesian Skyline Plot Insert) for Reading C Clade 1-1-4.** The Bayesian time-scaled maximum clade credibility (MCC) phylogeny and Bayesian Skyline plot (BSP) were reconstructed based on core SNPs identified among 100 representative isolates within Reading C clade 1-1-4; representative isolates were selected using time-stratified random sampling. The embedded BSP depicts the dynamics of the effective population size over time, where the y-axis represents the product of effective population size (*Ne*) and generation time (*τ*), and x-axis represents time in years. A bold line is used to display the median of *Neτ* across the complete time course, and the shaded area surrounding the line represents the 95% highest posterior density (HPD) interval. The MCC phylogeny next to the BSP represents time in years on the x-axis, and the branch lengths are reported in years. Red stars indicate the most recent common ancestors (MRCAs) of sub-clades I and II, and posterior probabilities of branch support are attached to the MRCA as well as important subsequent ancestors. Leaf nodes are color-coded to denote the isolation source category of the representative isolates, with the category “other” including isolates not collected from sources related to human, cattle, swine, chicken, and turkey. The following information is displayed adjacent to the leaf nodes: (i) collection date, (ii) geographical location, and (iii) sequence type. The bar plot on the far-right displays, for each representative isolate, the number of antimicrobial resistance (AMR) classes, based on the presence of AMR determinants in the genome. Bars are color-matched with the leaf nodes, and a vertical red dashed line is overlaid to distinguish multidrug-resistant (MDR) isolates (i.e., isolates resistant to at least one agent in at least three drug classes) from the rest. Sub-clades I and II show increased association with swine-related sources, with sub-clade II containing isolates mostly from 2019 and before while sub-clade I containing isolates mostly from later years. Colored rectangles are overlaid on both the MCC phylogeny and the BSP to highlight important time blocks associated with the clonal expansion of sub-clades I (2012-2015; light-red) and II (2015-2021; dark-red), respectively. After the beginning of the light-red time block, there is an increase in the occurrence of short branches and coalescent events within sub-clade II, hallmarks of declining genomic diversity that correspond well with the drop in *Neτ* shown within the light-red time block on the BSP. Upon entry into the dark-red time block, there is an increased density of short branches and coalescent events within sub-clade I, suggesting a decline in genomic diversity within this sub-clade, corresponding well with the further reduction in *Neτ* within the dark-red time block on the BSP.

**Figure S21.** **Time-Scaled Phylogeny (with Bayesian Skyline Plot Insert) for Cerro A Clade 3.** The Bayesian time-scaled maximum clade credibility (MCC) phylogeny and Bayesian Skyline plot (BSP) were reconstructed based on core SNPs identified among 100 representative isolates within Cerro A clade 3; representative isolates were selected using time-stratified random sampling. The embedded BSP depicts the dynamics of the effective population size over time, with the y-axis representing the product of effective population size (*Ne*) and generation time (*τ*) and x-axis representing time in years. A bold line is used to display the median of *Neτ* across the complete time course, and the shaded area surrounding the line represents the 95% highest posterior density (HPD) interval. The MCC phylogeny next to the BSP represents time in years on the x-axis, and the branch lengths are reported in years. The red star indicates the most recent common ancestor (MRCA) of sub-clade I, with the posterior probability of branch support next to it. Leaf nodes are color-coded to denote the isolation source category of the representative isolates, with the category “other” including isolates not collected from sources related to human, cattle, swine, chicken, and turkey. The following information is displayed adjacent to the leaf nodes: (i) collection date, (ii) geographical location, and (iii) sequence type. The bar plot on the far-right displays, for each representative isolate, the number of antimicrobial resistance (AMR) classes, based on the presence of AMR determinants in the genome. Bars are color-matched with the leaf nodes, and a vertical red dashed line is overlaid to distinguish multidrug-resistant (MDR) isolates (i.e., isolates resistant to at least one agent in at least three drug classes) from the rest.

**Figure S22. Time-Scaled phylogeny (with Bayesian Skyline Plot Insert) for Montevideo A Clade 10.** The Bayesian time-scaled maximum clade credibility (MCC) phylogeny and Bayesian Skyline plot (BSP) were reconstructed based on core SNPs identified among 100 representative isolates within Montevideo A clade 10; representative isolates were selected using time-stratified random sampling. The embedded BSP depicts the dynamics of the effective population size over time, with the y-axis representing the product of effective population size (*Ne*) and generation time (*τ*) and x-axis representing time in years. A bold line is used to display the median of *Neτ* across the complete time course, and the shaded area surrounding the line represents the 95% highest posterior density (HPD) interval. The MCC phylogeny next to the BSP represents time in years on the x-axis, and the branch lengths are reported in years. Red stars indicate the most recent common ancestor (MRCA) of sub-clades I-III, with the posterior probability of branch support next to them. Leaf nodes are color-coded to denote the isolation source category of the representative isolates, with the category “other” including isolates not collected from sources related to human, cattle, swine, chicken, and turkey. The following information is displayed adjacent to the leaf nodes: (i) collection date, (ii) geographical location, and (iii) sequence type. The bar plot on the far-right displays, for each representative isolate, the number of antimicrobial resistance (AMR) classes, based on the presence of AMR determinants in the genome. Bars are color-matched with the leaf nodes, and a vertical red dashed line is overlaid to distinguish multidrug-resistant (MDR) isolates (i.e., isolates resistant to at least one agent in at least three drug classes) from the rest.

**Figure S23.** **Time-Scaled Phylogeny (with Bayesian Skyline Plot Insert) for Kentucky B Clade 2.** The Bayesian time-scaled maximum clade credibility (MCC) phylogeny and Bayesian Skyline plot (BSP) were reconstructed based on core SNPs identified among 100 representative isolates within Kentucky B clade 2; representative isolates were selected using time-stratified random sampling. The embedded BSP depicts the dynamics of the effective population size over time, with the y-axis representing the product of effective population size (*Ne*) and generation time (*τ*) and x-axis representing time in years. A bold line is used to display the median of *Neτ* across the complete time course, and the shaded area surrounding the line represents the 95% highest posterior density (HPD) interval. The MCC phylogeny next to the BSP represents time in years on the x-axis, and the branch lengths are reported in years. Red stars indicate the most recent common ancestors (MRCA) of sub-clades I and II, with the posterior probability of branch support next to them. Leaf nodes are color-coded to denote the isolation source category of the representative isolates, with the category “other” including isolates not collected from sources related to human, cattle, swine, chicken, and turkey. The following information is displayed adjacent to the leaf nodes: (i) collection date, (ii) geographical location, and (iii) sequence type. The bar plot on the far-right displays, for each representative isolate, the number of antimicrobial resistance (AMR) classes, based on the presence of AMR determinants in the genome. Bars are color-matched with the leaf nodes, and a vertical red dashed line is overlaid to distinguish multidrug-resistant (MDR) isolates (i.e., isolates resistant to at least one agent in at least three drug classes) from the rest. A red rectangle is overlaid on both the MCC phylogeny and the BSP to highlight the time block that involves a notable reduction in *Ne*τ. By cross-referencing the MCC phylogeny and the BSP, it appears that there is an increase in the occurrence of short branches and the density of coalescent events within sub-clade I. However, these changes were likely due to ascertainment biases and thus not reflective of real changes in *Neτ* (see the text for details).

**Figure S24.** **Schematic of the in silico serotyping workflow.** This customized workflow utilizes SISTR, SeqSero2, and additional methods to validate the serotype of candidate SNP clusters and singletons. The workflow consists of five steps: (i) determining the serotype of all assemblies using SISTR; (ii) for the remaining questionable assemblies (i.e., assemblies with ambiguous results) representing SNP clusters, determining the serotype of all assemblies within the corresponding SNP clusters using SISTR; (iii) determining the serotype of the remaining questionable assemblies using SeqSero2 with the genome assembly workflow; (iv) determining the serotype of the remaining questionable assemblies using SeqSero2 with the allele microassembly workflow; and (v) if antigen gene sequences are not sufficient for unambiguously serotyping an assembly, using additional genetic markers (e.g., *flhB* for distinguishing isolates from serovars Enteritidis and Gallinarum) and/or phylogenetic affinity to infer its serotype.

**Figure S25.** **Results of the Date-Randomization Test performed on selected clades.** The 95% highest posterior density (HPD) interval of the evolutionary rate estimated using the real dataset does not overlap with those estimated using the randomized datasets for all selected clades, suggesting sufficient temporal signals contained by the datasets for measuring evolutionary changes.

**REFERENCE**

1. Souvorov A, Agarwala R, Lipman DJ. 2018. SKESA: strategic k-mer extension for scrupulous assemblies. Genome Biol 19:153.

2. Gurevich A, Saveliev V, Vyahhi N, Tesler G. 2013. QUAST: quality assessment tool for genome assemblies. Bioinformatics 29:1072–1075.

3. Yoshida CE, Kruczkiewicz P, Laing CR, Lingohr EJ, Gannon VPJ, Nash JHE, Taboada EN. 2016. The *Salmonella* In Silico Typing Resource (SISTR): an open web-accessible tool for rapidly typing and subtyping draft *Salmonella* genome assemblies. PLoS ONE 11:e0147101.

4. Zhang S, Bakker HC den, Li S, Chen J, Dinsmore BA, Lane C, Lauer AC, Fields PI, Deng X. 2019. SeqSero2: rapid and improved salmonella serotype determination using whole-genome sequencing data. Appl Environ Microbiol 01746-19.

5. Chen R, Cheng RA, Wiedmann M, Orsi RH. 2022. Development of a genomics-based approach to identify putative hypervirulent nontyphoidal *Salmonella* isolates: *Salmonella enterica* serovar Saintpaul as a model. mSphere 7:e00730-21.

6. Cheng RA, Orsi RH, Wiedmann M. 2021. Phylogeographic clustering suggests that distinct clades of *Salmonella enterica* serovar Mississippi are endemic in Australia, the United Kingdom, and the United States. mSphere r:e00485-21.

7. Gardner SN, Slezak T, Hall BG. 2015. kSNP3.0: SNP detection and phylogenetic analysis of genomes without genome alignment or reference genome. Bioinformatics 31:2877–2878.

8. Price MN, Dehal PS, Arkin AP. 2010. FastTree 2 – Approximately Maximum-Likelihood Trees for Large Alignments. PLoS ONE 5:e9490.

9. Miura RM (ed). 1986. Some mathematical questions in biology: DNA sequence analysis. American Mathematical Society, Providence, RI.

10. Yang Z. 1994. Maximum likelihood phylogenetic estimation from DNA sequences with variable rates over sites: approximate methods. J Mol Evol 39:39–306.

11. Shimodaira H, Hasegawa M. 1999. Multiple comparisons of log-likelihoods with applications to phylogenetic inference. Mol Biol Evol 16:1114.

12. Letunic I, Bork P. 2007. Interactive Tree Of Life (iTOL): an online tool for phylogenetic tree display and annotation. Bioinformatics 23:127–128.

13. Tonkin-Hill G, Lees JA, Bentley SD, Frost SDW, Corander J. 2019. Fast hierarchical Bayesian analysis of population structure. Nucleic Acids Res 47:5539–5549.

14. R Core Team. 2022. R: a language and environment for statistical computing. R Foundation for Statistical Computing, Vienna, Austria. URL https://www.R-project.org/.

15. Jolley KA, Maiden MC. 2010. BIGSdb: Scalable analysis of bacterial genome variation at the population level. BMC Bioinform 11:595.

16. Jia B, Raphenya AR, Alcock B, Waglechner N, Guo P, Tsang KK, Lago BA, Dave BM, Pereira S, Sharma AN, Doshi S, Courtot M, Lo R, Williams LE, Frye JG, Elsayegh T, Sardar D, Westman EL, Pawlowski AC, Johnson TA, Brinkman FSL, Wright GD, McArthur AG. 2017. CARD 2017: expansion and model-centric curation of the comprehensive antibiotic resistance database. Nucleic Acids Res 45:D566–D573.

17. Chen S, Zhou Y, Chen Y, Gu J. 2018. fastp: an ultra-fast all-in-one FASTQ preprocessor. Bioinformatics 34:i884–i890.

18. Croucher NJ, Page AJ, Connor TR, Delaney AJ, Keane JA, Bentley SD, Parkhill J, Harris SR. 2015. Rapid phylogenetic analysis of large samples of recombinant bacterial whole genome sequences using Gubbins. Nucleic Acids Res 43:e15–e15.

19. Page AJ, Taylor B, Delaney AJ, Soares J, Seemann T, Keane JA, Harris SR. 2016. SNP-sites: rapid efficient extraction of SNPs from multi-FASTA alignments. Microb Genom 2:e000056.

20. Duchêne S, Duchêne D, Holmes EC, Ho SYW. 2015. The performance of the date-randomization test in phylogenetic analyses of time-structured virus data. Mol Biol Evol 32:1895–1906.

21. Bouckaert R, Drummond A, Rambaut A, Suchard M. 2019. BEAUti - Bayesian Evolutionary Analysis Utility (2.5.2). https://github.com/beast-dev/beast-mcmc. Retrieved 1 September 2021.

22. Hawkey J, Le Hello S, Doublet B, Granier SA, Hendriksen RS, Fricke WF, Ceyssens P-J, Gomart C, Billman-Jacobe H, Holt KE, Weill F-X. 2019. Global phylogenomics of multidrug-resistant *Salmonella* *enterica* serotype Kentucky ST198. Microb Genom 5.

23. Deng X, Desai PT, den Bakker HC, Mikoleit M, Tolar B, Trees E, Hendriksen RS, Frye JG, Porwollik S, Weimer BC, Wiedmann M, Weinstock GM, Fields PI, McClelland M. 2014. Genomic epidemiology of *Salmonella enterica* serotype Enteritidis based on population structure of prevalent lineages. Emerg Infect Dis 20:1481–1489.

24. Kovac J, Cummings KJ, Rodriguez-Rivera LD, Carroll LM, Thachil A, Wiedmann M. 2017. Temporal genomic phylogeny reconstruction indicates a geospatial transmission path of *Salmonella* Cerro in the United States and a clade-specific loss of hydrogen sulfide production. Front Microbiol 8:737.

25. Miller EA, Elnekave E, Flores-Figueroa C, Johnson A, Kearney A, Munoz-Aguayo J, Tagg KA, Tschetter L, Weber BP, Nadon CA, Boxrud D, Singer RS, Folster JP, Johnson TJ. 2020. Emergence of a novel *Salmonella enterica* serotype Reading clonal group is linked to its expansion in commercial turkey production, resulting in unanticipated human illness in North America. mSphere.00056-20.

26. Rieux A, Khatchikian CE. 2017. tipdatingbeast: an r package to assist the implementation of phylogenetic tip-dating tests using beast. Mol Ecol Resour 17:608–613.

27. Bouckaert R, Vaughan TG, Barido-Sottani J, Duchêne S, Fourment M, Gavryushkina A, Heled J, Jones G, Kühnert D, Maio ND, Matschiner M, Mendes FK, Müller NF, Ogilvie HA, Plessis L du, Popinga A, Rambaut A, Rasmussen D, Siveroni I, Suchard MA, Wu C-H, Xie D, Zhang C, Stadler T, Drummond AJ. 2019. BEAST 2.5: an advanced software platform for Bayesian evolutionary analysis. PLoS Comput Biol 15:e1006650.

28. Bouckaert RR, Drummond AJ. 2017. bModelTest: Bayesian phylogenetic site model averaging and model comparison. BMC Evol Biol 17:42.

29. Xie W, Lewis PO, Fan Y, Kuo L, Chen M-H. 2011. Improving marginal likelihood estimation for Bayesian phylogenetic model selection. Syst Biol 60:150–160.

30. Rambaut A, Drummond A. 2019. LogCombiner (2.5.2). http://beast2.cs.auckland.ac.nz/. Retrieved 1 September 2021.

31. Rambaut A, Drummond AJ, Xie D, Baele G, Suchard MA. 2018. Posterior summarization in Bayesian phylogenetics using Tracer 1.7. Syst Biol 67:901–904.

32. Heled J, Bouckaert RR. 2013. Looking for trees in the forest: summary tree from posterior samples. BMC Evol Biol 13:221.

33. Drummond A. 2018. FigTree (1.4.4). http://tree.bio.ed.ac.uk/. Retrieved 1 September 2021.

34. Yu G, Lam TT-Y, Zhu H, Guan Y. 2018. Two methods for mapping and visualizing associated data on phylogeny using ggtree. Mol Biol Evol 35:3041–3043.

35. Yu G, Smith DK, Zhu H, Guan Y, Lam TT-Y. 2017. ggtree: an r package for visualization and annotation of phylogenetic trees with their covariates and other associated data. Methods Ecol Evol 8:28–36.

36. Yu G. 2022. Data Integration, Manipulation and Visualization of Phylogenetic Trees, 1st ed. Chapman and Hall/CRC, Boca Raton, FL.

37. Wang L-G, Lam TT-Y, Xu S, Dai Z, Zhou L, Feng T, Guo P, Dunn CW, Jones BR, Bradley T, Zhu H, Guan Y, Jiang Y, Yu G. 2020. Treeio: an R package for phylogenetic tree input and output with richly annotated and associated data. Mol Biol Evol 37:599–603.

38. Lycett S, Hodcroft E. 2020. Cluster Picker (1.2.5). https://github.com/emmahodcroft/cluster-picker-and-cluster-matcher. Retrieved 1 September 2021.

39. Hodcroft E. 2020. Cluster Matcher (1.2.7). https://github.com/emmahodcroft/cluster-picker-and-cluster-matcher. Retrieved 1 September 2021.
