# Supplementary material for: Salmonella associated with agricultural animals exhibit diverse evolutionary rates and show evidence of recent clonal expansion
Source: mBio. 2024 Sep 17;15(10):e01913-24. doi: 10.1128/mbio.01913-24 (PMC11492988; doi:10.1128/mbio.01913-24)
Supplement: Supplemental Tables — Tables S1 to S3. [file mbio.01913-24-s0005.docx]

**Table S1** Summary statistics of the source association analysis performed for clades with > 100 non-human isolates.

| Host | Number of non-human Isolates | Number of Isolates from Host | | |  | Number of Isolates not from Host | | | Odds Ratio | Odd Ratio after removing outliers^a^ | BH-corrected *P*-value^b^ | Association^c^ |  |  |  |
| --- | --- | --- | --- | --- | --- | --- | --- | --- | --- | --- | --- | --- | --- | --- | --- |
|  |  | In Clade | Not in Clade | |  | In Clade | Not in Clade | |  |  |  |  | |  |  |
| **Cerro A Clade 2** | | | |  | | | |  | | | | |  |  |  |
| Cattle | 1,341 | 1,042 | 11,123 | |  | 299 | 83,283 | | 26.093 | 25.142 | < 0.001 | Overrepresentation | |  |  |
| Swine | 1,341 | 34 | 12,616 | |  | 1,307 | 81,790 | | 0.169 | NC | < 0.001 | Underrepresentation | |  |  |
| Chicken | 1,341 | 21 | 26,475 | |  | 1,320 | 67,931 | | 0.041 | NC | < 0.001 | Underrepresentation | |  |  |
| Turkey | 1,341 | 2 | 7,188 | |  | 1,339 | 87,218 | | 0.018 | NC | < 0.001 | Underrepresentation | |  |  |
| Other | 1,341 | 242 | 37,004 | |  | 1,099 | 57,402 | | 0.342 | NC | < 0.001 | Neutral | |  |  |
| **Cerro A Clade 3** | | | |  | | | |  | | | | |  | | |
| Cattle | 97 | 4 | 12,161 | |  | 93 | 83,489 | | 0.295 | NC | 0.004 | Neutral | |  |  |
| Swine^d^ | 97 | 71 | 12,579 | |  | 26 | 83,071 | | 18.000 | 17.526 | < 0.001 | Overrepresentation | |  |  |
| Chicken | 97 | 6 | 26,490 | |  | 91 | 69,160 | | 0.172 | NC | < 0.001 | Underrepresentation | |  |  |
| Turkey | 97 | 0 | 7,190 | |  | 97 | 88,460 | | 0.000 | NC | < 0.001 | Underrepresentation | |  |  |
| Other | 97 | 16 | 37,230 | |  | 81 | 58,420 | | 0.310 | NC | < 0.001 | Neutral | |  |  |
| **Dublin Clade 2-3** | | | |  | | | |  | | | | |  | | |
| Cattle | 1,701 | 1,586 | 10,579 | |  | 115 | 83,467 | | 108.812 | 102.637 | < 0.001 | Overrepresentation | |  |  |
| Swine | 1,701 | 5 | 12,645 | |  | 1,696 | 81,401 | | 0.019 | NC | < 0.001 | Underrepresentation | |  |  |
| Chicken | 1,701 | 5 | 26,491 | |  | 1,696 | 67,555 | | 0.008 | NC | < 0.001 | Underrepresentation | |  |  |
| Turkey | 1,701 | 3 | 7,187 | |  | 1,698 | 86,859 | | 0.021 | NC | < 0.001 | Underrepresentation | |  |  |
| Other | 1,701 | 102 | 37,144 | |  | 1,599 | 56,902 | | 0.098 | NC | < 0.001 | Underrepresentation | |  |  |
| **Enteritidis A Clade 7** | | | |  | | | |  | | | | |  | |  |
| Cattle | 7,801 | 77 | 12,088 | |  | 7,724 | 75,858 | | 0.063 | NC | < 0.001 | Underrepresentation | |  |  |
| Swine | 7,801 | 80 | 12,570 | |  | 7,721 | 75,376 | | 0.062 | NC | < 0.001 | Underrepresentation | |  |  |
| Chicken^e^ | 7,801 | 4,775 | 21,721 | |  | 3,026 | 66,225 | | 4.811 | 4.760 | < 0.001 | Overrepresentation | |  |  |
| Turkey | 7,801 | 56 | 7,134 | |  | 7,745 | 80,812 | | 0.082 | NC | < 0.001 | Underrepresentation | |  |  |
| Other | 7,801 | 2,813 | 34,433 | |  | 4,988 | 53,513 | | 0.876 | NC | < 0.001 | Neutral | |  |  |
| **Infantis A Clade 1-2** | | | |  | | | |  | | | | |  | | |
| Cattle | 1,569 | 168 | 11,997 | |  | 1,401 | 82,181 | | 0.821 | NC | 0.009 | Neutral | |  |  |
| Swine^f^ | 1,569 | 649 | 12,001 | |  | 920 | 82,177 | | 4.830 | 4.756 | < 0.001 | Overrepresentation | |  |  |
| Chicken | 1,569 | 336 | 26,160 | |  | 1,233 | 68,018 | | 0.709 | NC | < 0.001 | Neutral | |  |  |
| Turkey | 1,569 | 35 | 7,155 | |  | 1,534 | 87,023 | | 0.278 | NC | < 0.001 | Neutral | |  |  |
| Other | 1,569 | 381 | 36,865 | |  | 1,188 | 57,313 | | 0.499 | NC | < 0.001 | Neutral | |  |  |
| **Infantis A Clade 1-3** | | | |  | | | |  | | | | |  | | |
| Cattle | 6,035 | 67 | 12,098 | |  | 5,968 | 77,614 | | 0.072 | NC | < 0.001 | Underrepresentation | |  |  |
| Swine | 6,035 | 226 | 12,424 | |  | 5,809 | 77,288 | | 0.242 | NC | < 0.001 | Neutral | |  |  |
| Chicken | 6,035 | 4,599 | 21,897 | |  | 1,436 | 67,815 | | 9.919 | 9.919 | < 0.001 | Overrepresentation | |  |  |
| Turkey | 6,035 | 237 | 6,953 | |  | 5,798 | 82,759 | | 0.487 | NC | < 0.001 | Neutral | |  |  |
| Other | 6,035 | 906 | 36,340 | |  | 5,129 | 53,372 | | 0.259 | NC | < 0.001 | Neutral | |  |  |
| **Infantis A Clade 1-5** | | | |  | | | |  | | | | |  | | |
| Cattle | 175 | 1 | 12,164 | |  | 174 | 83,408 | | 0.039 | NC | < 0.001 | Underrepresentation | |  |  |
| Swine | 175 | 32 | 12,618 | |  | 143 | 82,954 | | 1.471 | NC | 0.035 | Neutral | |  |  |
| Chicken | 175 | 34 | 26,462 | |  | 141 | 69,110 | | 0.630 | NC | 0.009 | Neutral | |  |  |
| Turkey | 175 | 0 | 7,190 | |  | 175 | 88,382 | | 0.000 | NC | < 0.001 | Underrepresentation | |  |  |
| Other | 175 | 108 | 37,138 | |  | 67 | 58,434 | | 2.536 | NC | < 0.001 | Neutral | |  |  |
| **Kentucky A Clade 1** | | | |  | | | |  | | | | |  | | |
| Cattle | 7,710 | 199 | 11,966 | |  | 7,511 | 76,071 | | 0.168 | NC | < 0.001 | Underrepresentation | |  |  |
| Swine | 7,710 | 44 | 12,606 | |  | 7,666 | 75,431 | | 0.034 | NC | < 0.001 | Underrepresentation | |  |  |
| Chicken | 7,710 | 7,027 | 19,469 | |  | 683 | 68,568 | | 36.235 | 35.750 | < 0.001 | Overrepresentation | |  |  |
| Turkey | 7,710 | 36 | 7,154 | |  | 7,674 | 80,883 | | 0.053 | NC | < 0.001 | Underrepresentation | |  |  |
| Other | 7,710 | 404 | 36,842 | |  | 7,306 | 51,195 | | 0.077 | NC | < 0.001 | Underrepresentation | |  |  |
| **Kentucky A Clade 3** | | | |  | | | |  | | | | |  | | |
| Cattle | 135 | 10 | 12,155 | |  | 125 | 83,457 | | 0.549 | NC | 0.045 | Neutral | |  |  |
| Swine | 135 | 12 | 12,638 | |  | 123 | 82,974 | | 0.641 | NC | 0.091 | Neutral | | | |
| Chicken | 135 | 32 | 26,464 | |  | 103 | 69,148 | | 0.812 | NC | 0.175 | Neutral | | | |
| Turkey | 135 | 0 | 7,190 | |  | 135 | 88,422 | | 0.000 | NC | < 0.001 | Underrepresentation | | | |
| Other | 135 | 81 | 37,165 | |  | 54 | 58,447 | | 2.359 | NC | < 0.001 | Neutral | | | |
| **Kentucky B Clade 2** | | | |  | | | |  | | | | |  | | |
| Cattle | 178 | 100 | 12,065 | |  | 78 | 83,504 | | 8.873 | 8.607 | < 0.001 | Overrepresentation | | | |
| Swine | 178 | 0 | 12,650 | |  | 178 | 82,919 | | 0.000 | NC | < 0.001 | Underrepresentation | | | |
| Chicken | 178 | 8 | 26,488 | |  | 170 | 69,081 | | 0.123 | NC | < 0.001 | Underrepresentation | | | |
| Turkey | 178 | 21 | 7,169 | |  | 157 | 88,400 | | 1.649 | NC | 0.030 | Neutral | | | |
| Other | 178 | 49 | 37,197 | |  | 129 | 58,372 | | 0.596 | NC | 0.001 | Neutral | | | |
| **Kentucky B Clade 4** | | | |  | | | |  | | | | |  | | |
| Cattle | 218 | 9 | 12,156 | |  | 209 | 83,373 | | 0.295 | NC | < 0.001 | Neutral | | | |
| Swine | 218 | 38 | 12,612 | |  | 180 | 82,917 | | 1.388 | NC | 0.045 | Neutral | | | |
| Chicken | 218 | 47 | 26,449 | |  | 171 | 69,080 | | 0.718 | NC | 0.030 | Neutral | | | |
| Turkey | 218 | 0 | 7,190 | |  | 218 | 88,339 | | 0.000 | NC | < 0.001 | Underrepresentation | | | |
| Other | 218 | 124 | 37,122 | |  | 94 | 58,407 | | 2.076 | NC | < 0.001 | Neutral | | | |
| **Montevideo A Clade 1** | | | |  | | | |  | | | | |  | |  |
| Cattle | 375 | 13 | 12,152 | |  | 362 | 83,220 | | 0.246 | NC | < 0.001 | Neutral | | | |
| Swine | 375 | 27 | 12,623 | |  | 348 | 82,749 | | 0.509 | NC | < 0.001 | Neutral | | | |
| Chicken | 375 | 80 | 26,416 | |  | 295 | 68,956 | | 0.708 | NC | 0.003 | Neutral | | | |
| Turkey | 375 | 16 | 7,174 | |  | 359 | 88,198 | | 0.548 | NC | 0.007 | Neutral | | | |
| Other | 375 | 239 | 37,007 | |  | 136 | 58,365 | | 2.772 | NC | < 0.001 | Neutral | | | |
| **Montevideo A Clade 6** | | | |  | | | |  | | | | |  | |  |
| Cattle | 291 | 16 | 12,149 | |  | 275 | 83,307 | | 0.399 | NC | < 0.001 | Neutral | | | |
| Swine | 291 | 20 | 12,630 | |  | 271 | 82,826 | | 0.484 | NC | < 0.001 | Neutral | | | |
| Chicken | 291 | 53 | 26,443 | |  | 238 | 69,013 | | 0.581 | NC | < 0.001 | Neutral | | | |
| Turkey | 291 | 51 | 7,139 | |  | 240 | 88,317 | | 2.629 | NC | < 0.001 | Neutral | | | |
| Other | 291 | 151 | 37,095 | |  | 140 | 58,361 | | 1.697 | NC | < 0.001 | Neutral | | | |
| **Montevideo A Clade 7** | | | |  | | | |  | | | | |  | |  |
| Cattle | 206 | 3 | 12,162 | |  | 203 | 83,379 | | 0.101 | NC | < 0.001 | Underrepresentation | | | |
| Swine | 206 | 2 | 12,648 | |  | 204 | 82,893 | | 0.064 | NC | < 0.001 | Underrepresentation | | | |
| Chicken | 206 | 1 | 26,495 | |  | 205 | 69,046 | | 0.013 | NC | < 0.001 | Underrepresentation | | | |
| Turkey | 206 | 0 | 7,190 | |  | 206 | 88,351 | | 0.000 | NC | < 0.001 | Underrepresentation | | | |
| Other | 206 | 200 | 37,046 | |  | 6 | 58,495 | | 52.633 | 36.580 | < 0.001 | Overrepresentation | | | |
| **Montevideo A Clade 10** | | | |  | | | |  | | | | |  | |  |
| Cattle | 2,220 | 1,722 | 10,443 | |  | 498 | 83,084 | | 27.510 | 26.472 | < 0.001 | Overrepresentation | | | |
| Swine | 2,220 | 38 | 12,612 | |  | 2,182 | 80,915 | | 0.112 | NC | < 0.001 | Underrepresentation | | | |
| Chicken | 2,220 | 50 | 26,446 | |  | 2,170 | 67,081 | | 0.058 | NC | < 0.001 | Underrepresentation | | | |
| Turkey | 2,220 | 13 | 7,177 | |  | 2,207 | 86,350 | | 0.071 | NC | < 0.001 | Underrepresentation | | | |
| Other | 2,220 | 397 | 36,849 | |  | 1,823 | 56,678 | | 0.335 | NC | < 0.001 | Neutral | | | |
| **Reading A Clade 1-1-2** | | | |  | | | |  | | | | |  | |  |
| Cattle | 1,096 | 7 | 12,158 | |  | 1,089 | 82,493 | | 0.044 | NC | < 0.001 | Underrepresentation | | | |
| Swine | 1,096 | 4 | 12,646 | |  | 1,092 | 82,005 | | 0.024 | NC | < 0.001 | Underrepresentation | | | |
| Chicken | 1,096 | 31 | 26,465 | |  | 1,065 | 68,186 | | 0.075 | NC | < 0.001 | Underrepresentation | | | |
| Turkey | 1,096 | 1,009 | 6,181 | |  | 87 | 88,470 | | 166.000 | 139.019 | < 0.001 | Overrepresentation | | | |
| Other | 1,096 | 45 | 37,201 | |  | 1,051 | 57,450 | | 0.066 | NC | < 0.001 | Underrepresentation | | | |
| **Reading C Clade 1-1-4** | | | |  | | | |  | | | | |  | |  |
| Cattle | 225 | 83 | 12,082 | |  | 142 | 83,440 | | 4.037 | NC | < 0.001 | Neutral | | | |
| Swine^g^ | 225 | 97 | 12,553 | |  | 128 | 82,969 | | 5.009 | 4.854 | < 0.001 | Overrepresentation | | | |
| Chicken | 225 | 1 | 26,495 | |  | 224 | 69,027 | | 0.012 | NC | < 0.001 | Underrepresentation | | | |
| Turkey | 225 | 1 | 7,189 | |  | 224 | 88,333 | | 0.055 | NC | < 0.001 | Underrepresentation | | | |
| Other | 225 | 43 | 37,203 | |  | 182 | 58,319 | | 0.370 | NC | < 0.001 | Neutral | | | |

^a^ Outliers possibly representing strains obtained in response to an outbreak investigation or in a research project were removed and new OR were calculated as described in Materials and Methods. All adjusted *P*-values are < 0.01.

^b^ Fisher’s exact test *P*-value after correction for multiple comparisons using the Benjamini-Hochberg (BH) method.

^c^ The association of each clade with each source category. Unless otherwise specified, overrepresentation was indicated by odds ratio > 5 and BH-corrected *P*-value < 0.05, underrepresentation was indicated by odds ratio < 0.2 and BH-corrected *P*-value < 0.05, and neutral is indicated by 0.2 ≤ odds ratio ≤ 5 or BH-corrected *P*-value ≥ 0.05. The overrepresentation of a source category among isolates within a given clade suggests source association.

^d^ The swine source category was considered overrepresented among isolates within Cerro A clade 3, even though the clade contained a total of 97 non-human isolates, which was three isolates short of our criteria of 100 isolates. This decision was supported by the high swine association odds ratio (27.567; BH-corrected *P*-value < 0.001) obtained based on isolates from the US, the dominant country for this clade, which suggests a strong association without introduction of potential ascertainment biases.

^e^ Although Enteritidis A Clade 7 displayed a chicken association odds ratio after outlier removal of 4.760 (BH-corrected *P*-value < 0.001), which was slightly lower than the cutoff of 5, it was considered a chicken-associated clade due to the fact that (i) it constituted 98% of the isolates from *S.* Enteritidis, the serovar most frequently isolated from chicken with clinical symptoms (Morningstar et al., 2018), and (ii) the odds ratio for chicken association calculated based on isolates from the US was 10.126 (BH-corrected *P*-value < 0.001), suggestion a strong association without introduction of potential ascertainment biases.

^f^ The swine source category was considered overrepresented among isolates within Infantis A Clade 1-2, even though the clade showed a swine association odds ratio after outlier removal of 4.756 (BH-corrected *P-*value < 0.001), which was slightly lower than the cutoff of 5. This decision was supported by a previous study that reported the association of *S.* Infantis with pork between 1998 to 2008 (Jackson et al., 2013).

^g^ The swine source category was considered overrepresented among isolates within Reading C Clade 1-1-4, even though the clade showed a swine association odds ratio after outlier removal of 4.854 (BH-corrected *P-*value < 0.001), which was slightly lower than the cutoff of 5. This decision was supported by a previous study that reported the association of a *S.* Reading clade with swine (Miller et al., 2020).

**Table S2** Summary of the bModelTest analysis performed for selected clades^a^.

| Phylogenetic Group | Clade | Best Substitution Model | Grouping of Substitution Rates^b^ | | | | | | Proportion of Time with Gamma Rate Heterogeneity | Evolutionary Rate Coefficient of Variation (95% HPD Interval)^c^ |
| --- | --- | --- | --- | --- | --- | --- | --- | --- | --- | --- |
|  |  |  | ΑC | AG | AT | CG | CT | GT |  |  |
| **Enteritidis** |  |  |  |  |  |  |  |  |  |  |
| Enteritidis A | 7 | 23 | 1 | 2 | 3 | 4 | 2 | 1 | 0.992 | 0.303 (0.227-0.388) |
| **Kentucky** |  |  |  |  |  |  |  |  |  |  |
| Kentucky A | 1 | 23 | 1 | 2 | 3 | 4 | 2 | 1 | 0.995 | 0.362 (0.270-0.458) |
| Kentucky B | 2 | 23 | 1 | 2 | 3 | 4 | 2 | 1 | 0.804 | 0.606 (0.405-0.829) |
| **Montevideo** |  |  |  |  |  |  |  |  |  |  |
| Montevideo A | 7 | 23 | 1 | 2 | 3 | 4 | 2 | 1 | 0.983 | 0.289 (0.190-0.393) |
| Montevideo A | 10 | 27 | 1 | 2 | 3 | 4 | 5 | 1 | 0.976 | 0.554 (0.425-0.705) |

^a^ Five clades from three different serovars were selected as representatives for conducting the bModelTest analysis to determine (i) the best substitution model, (ii) whether to include gamma rate heterogeneity, and (iii) whether to use relaxed instead of strict molecular clock.

^b^ The way substitution rates are groups specifies the substitution model. Substitution rates with identical Arabic numerals are represented using a single parameter in the model.

^c^ Mean and 95% highest posterior density (HPD) interval of the coefficient of variation of evolutionary rate were inferred from the rate.coefficientOfVariation parameter estimates reported by Tracer. A mean value > 0.1 indicates the preference of a relaxed molecular clock over a strict clock.

**Table S3** Summary of the stepping-stone sampling analysis performed for selected clades^a^.

| Phylogenetic Group | Clade | Median Log-Transformed Marginal Likelihood | | |  | Pairwise Bayes Factor^b^ | | | Best Population Model |
| --- | --- | --- | --- | --- | --- | --- | --- | --- | --- |
|  |  | Constant Size (CS) | Exponential Growth (EG) | Bayesian Skyline (BS) |  | EG/CS | BS/CS | BS/EG |  |
| **Enteritidis** |  |  |  |  |  |  |  |  |  |
| Enteritidis A | 7 | -6610728.61 | -6610731.29 | -6610710.30 |  | 0.068 | 1.299 × 10^9^ | 8.89 × 10^7^ | BS |
| **Kentucky** |  |  |  |  |  |  |  |  |  |
| Kentucky A | 1 | -6851660.94 | -6851649.21 | -6851643.49 |  | 1.239 × 10^5^ | 3.803 × 10^7^ | 307.047 | BS |
| Kentucky B | 2 | -6650547.38 | -6650547.79 | -6650528.47 |  | 0.66 | 1.633 × 10^8^ | 2.475 × 10^8^ | BS |
| **Montevideo** |  |  |  |  |  |  |  |  |  |
| Montevideo A | 7 | -6714512.63 | -6714496.16 | -6714450.51 |  | 1.420 × 10^7^ | 9.476 × 10^26^ | 6.672 × 10^19^ | BS |
| Montevideo A | 10 | -6387177.66 | -6387167.78 | -6387161.43 |  | 1.952 × 10^4^ | 1.113 × 10^7^ | 570.207 | BS |

^a^ Five clades from three different serovars were selected as representatives for conducting the stepping-stone sampling analysis to identify the optimal population model among three candidate models: (i) coalescent constant size (CS), (ii) coalescent exponential growth (EG), and (iii) coalescent Baysian skyline (BS).

^b^ Pairwise comparisons across population models were performed based on the Bayes factor. For each comparison, a Bayes factor > 1 supports the preference of the model as the numerator, while a Bayes factor < 1 supports the preference of the model as the denominator.
